# Supplementary material for: Gene-vegetarianism interactions in calcium, estimated glomerular filtration rate, and testosterone identified in genome-wide analysis across 30 biomarkers
Source: PLoS Genet. 2024 Jul 11;20(7):e1011288. doi: 10.1371/journal.pgen.1011288 (PMC11239071; doi:10.1371/journal.pgen.1011288)
Supplement: S1 Fig — Starting from the entire UK Biobank, visualizing the the number of participants who were excluded in each quality control step, to identify the 2,328 strict European (EUR) vegetarians used in this study. 24HR, 24-hour recall survey, QC, quality control. (PDF) [file pgen.1011288.s011.pdf]

S1

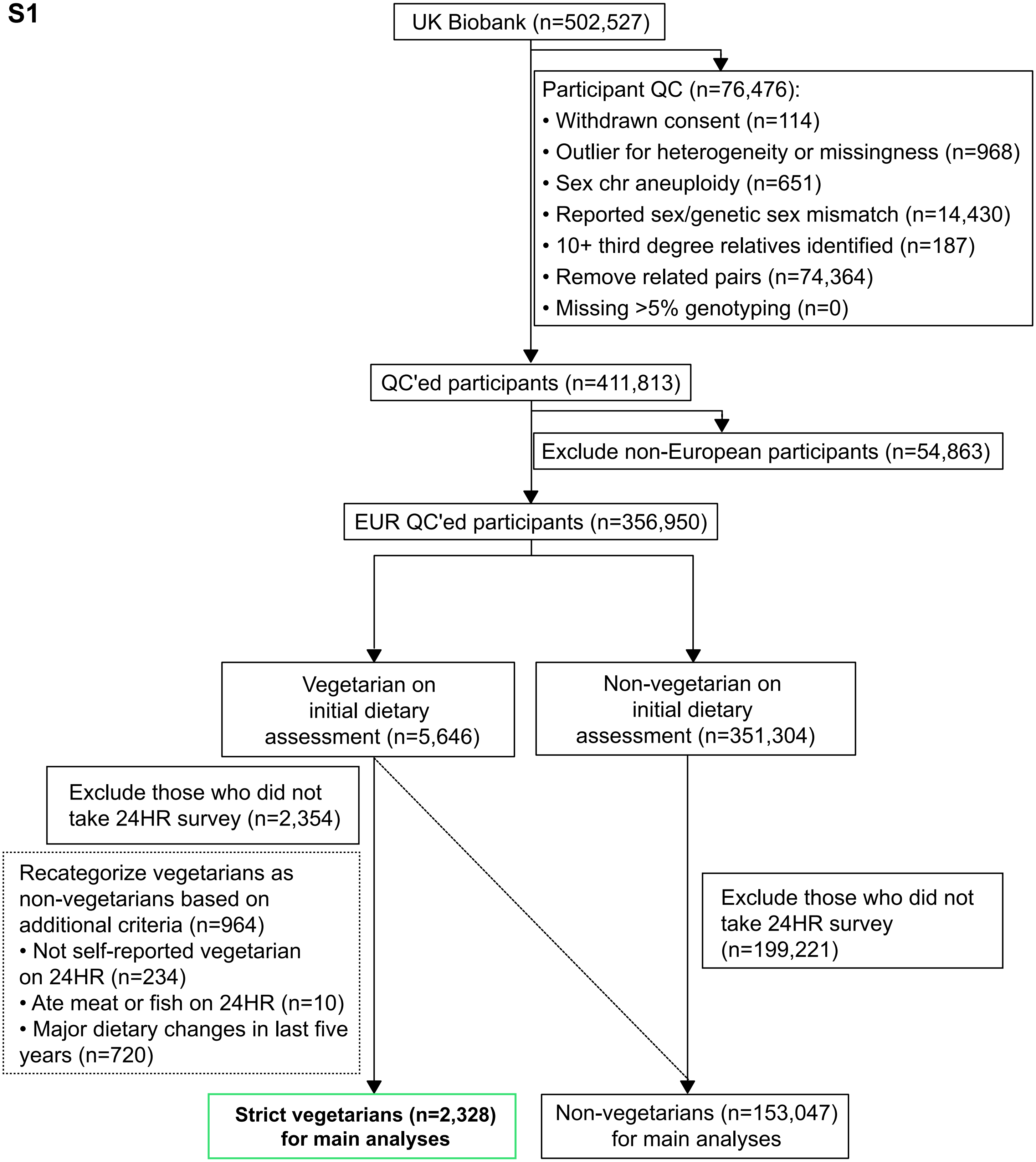

S1 Fig. Participant flow chart. Starting from the entire UK Biobank, visualizing the the number of participants who were excluded in each quality control step, to identify the 2,328 strict European (EUR) vegetarians used in this study. 24HR, 24 hour recall survey, QC, quality control.
