# Supplementary material for: Gene-vegetarianism interactions in calcium, estimated glomerular filtration rate, and testosterone identified in genome-wide analysis across 30 biomarkers
Source: PLoS Genet. 2024 Jul 11;20(7):e1011288. doi: 10.1371/journal.pgen.1011288 (PMC11239071; doi:10.1371/journal.pgen.1011288)
Supplement: S2 Fig — Comparing raw values of European strict vegetarians and nonvegetarians across 30 biomarker traits. Boxplots show first decile, first quartile, median, third quartile, and last decile. Units of each biomarker can be found in S2 Table. (a) Combined male and female cohort. Dot and label refer to mean. (b) Stratified by sex. (PDF) [file pgen.1011288.s012.pdf]

## S2a

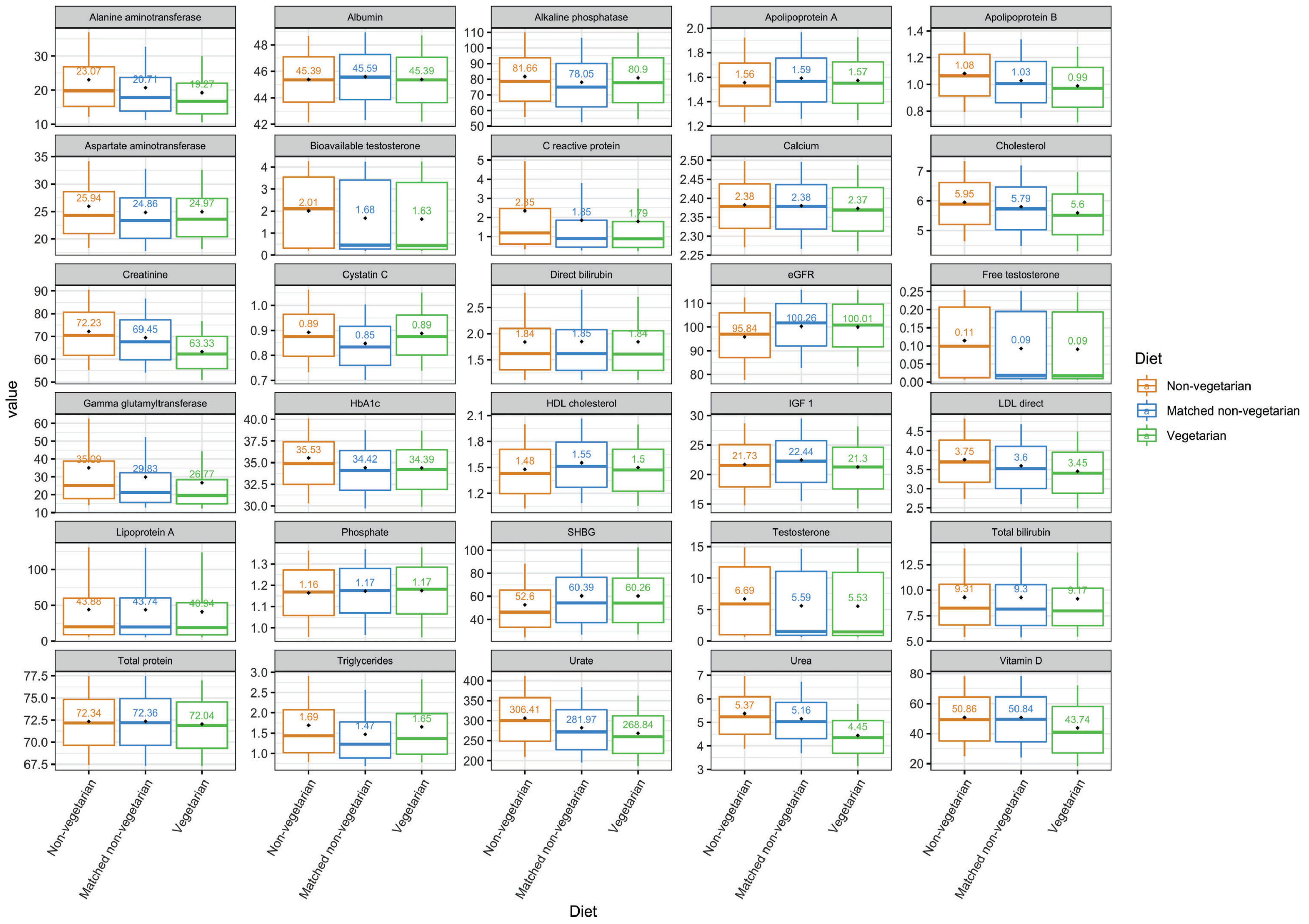
Alanine aminotransferase
23.07
20.71
Albumin
45.39
45.59
45.39
Alkaline phosphatase
81.66
78.05
80.9
Apolipoprotein A
1.56
1.59
1.57
Apolipoprotein B
1.08
1.03
0.99
Aspartate aminotransferase
25.94
24.86
24.97
Bioavailable testosterone
2.01
1.68
1.63
C reactive protein
Calcium
2.38
2.38
2.37
Cholesterol
5.95
5.79
5.6
Creatinine
72.23
69.45
63.33
Cystatin C
0.89
0.85
0.89
Direct bilirubin
eGFR
Free testosterone
0.11
0.09
0.09
Gamma glutamyltransferase
HbA1c
35.53
34.42
34.39
HDL cholesterol
1.48
1.55
1.5
IGF 1
21.73
22.44
21.3
LDL direct
3.75
3.6
3.45
Lipoprotein A
Phosphate
1.16
1.17
1.17
SHBG
52.6
60.39
60.26
Testosterone
6.69
5.59
5.53
Total bilirubin
Total protein
72.34
72.36
72.04
Triglycerides
1.69
Urate
306.41
281.97
268.84
Urea
5.37
5.16
4.45
Vitamin D
50.86
50.84
43.74
value
Diet
Non-vegetarian
Matched non-vegetarian
Vegetarian
Diet
Non-vegetarian
Matched non-vegetarian
Vegetarian

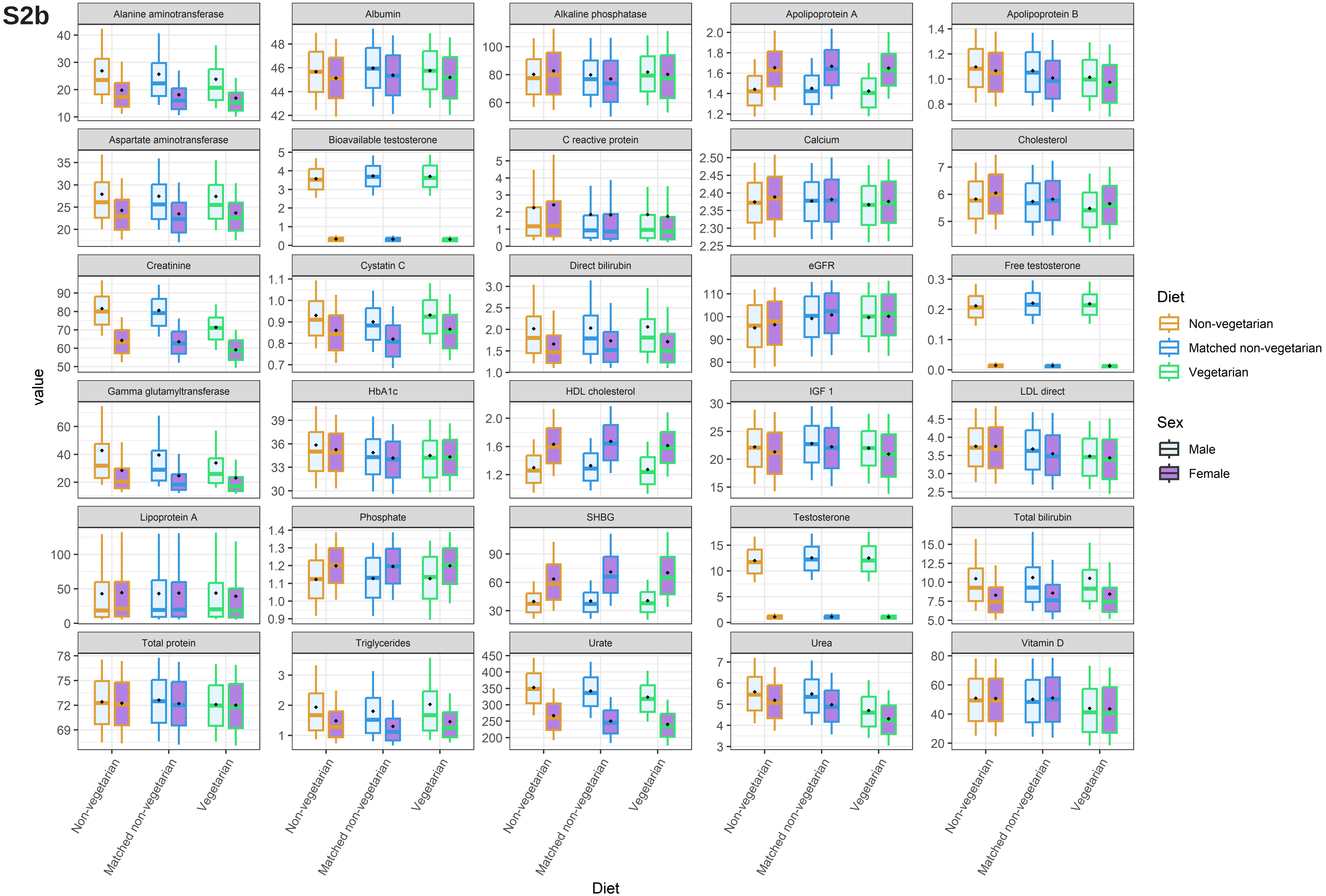


**S2 Fig. Boxplots of unadjusted biomarker levels.** Comparing raw values of European strict vegetarians and nonvegetarians across 30 biomarker traits. Boxplots show first decile, first quartile, median, third quartile, and last decile. Units of each biomarker can be found in S2 Table. (**a**) Combined male and female cohort. Dot and label refer to mean. (**b**) Stratified by sex.
