# Supplementary material for: Gene-vegetarianism interactions in calcium, estimated glomerular filtration rate, and testosterone identified in genome-wide analysis across 30 biomarkers
Source: PLoS Genet. 2024 Jul 11;20(7):e1011288. doi: 10.1371/journal.pgen.1011288 (PMC11239071; doi:10.1371/journal.pgen.1011288)
Supplement: S3 Fig — Plot shows the absolute standardized mean difference of model covariates in nonvegetarians before and after matching with vegetarians for effects estimation. After matching, the ASMD in all model covariates were <0.05 standardized units. BMI, body mass index; AlcoholFreq, frequency of alcohol usage (<3 drinks/week or ≥ 3 drinks/week); zTownsend, standardized Townsend deprivation index; PCA, genetic principal component; distance, matching distance between partici-pants as calculated by general linearized model. (PDF) [file pgen.1011288.s013.pdf]

S3

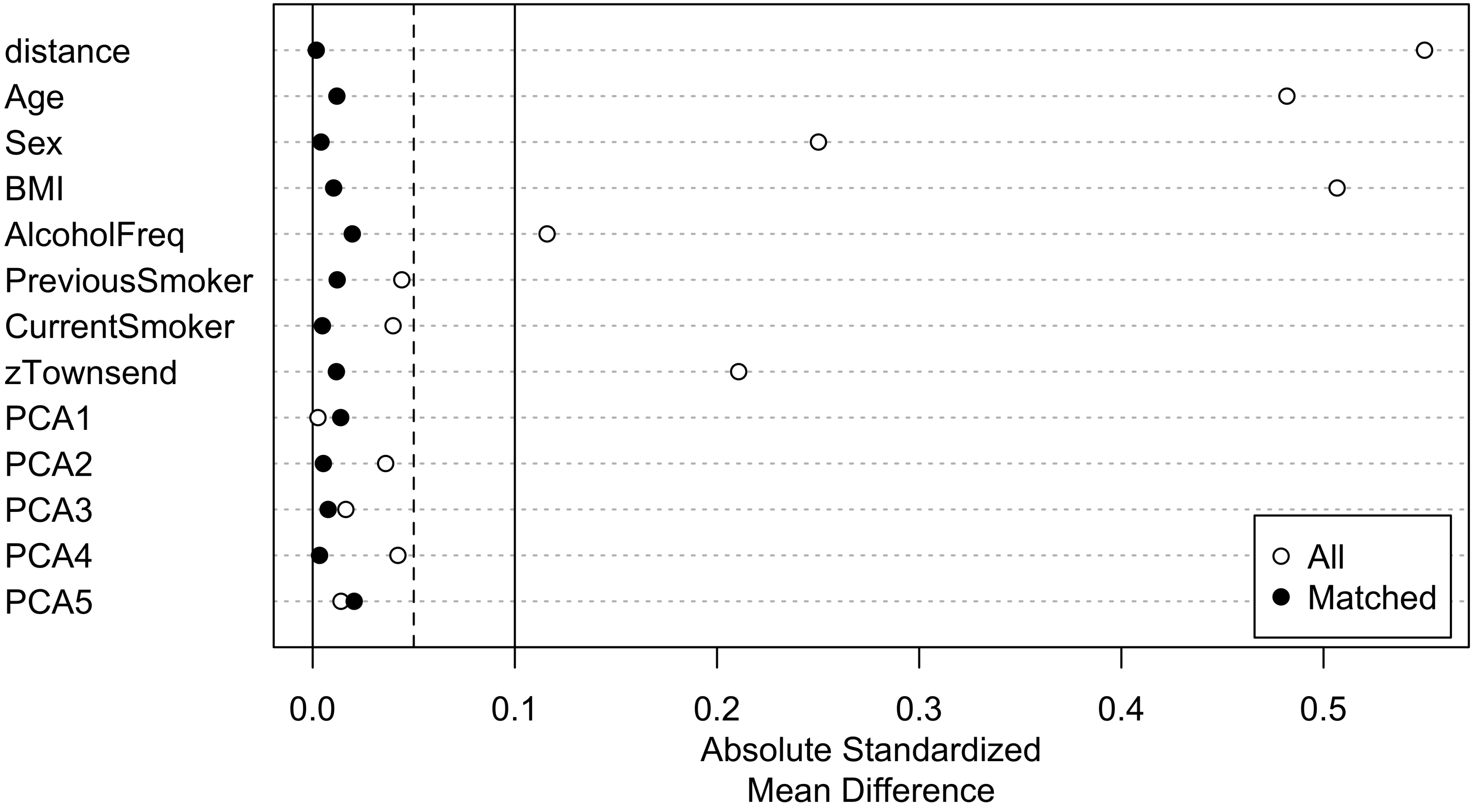

S3 Fig. Love plot of covariates before and after matching. Plot shows the absolute standardized mean difference of model covariates in nonvegetarians before and after matching with vegetarians for effects estimation. After matching, the ASMD in all model covariates were <0.05 standardized units. BMI, body mass index; AlcoholFreq, frequency of alcohol usage (<3 drinks/week or ≥ 3 drinks/week); zTownsend, standardized Townsend deprivation index; PCA, genetic principal component; distance, matching distance between partici-pants as calculated by general linearized model.
