# Supplementary material for: Gene-vegetarianism interactions in calcium, estimated glomerular filtration rate, and testosterone identified in genome-wide analysis across 30 biomarkers
Source: PLoS Genet. 2024 Jul 11;20(7):e1011288. doi: 10.1371/journal.pgen.1011288 (PMC11239071; doi:10.1371/journal.pgen.1011288)
Supplement: S4 Fig — Effects estimation for vegetarianism in sex-stratified BMI-adjusted model. Error bars indicate 95% confidence interval. Bonferroni-corrected significance threshold at α = 0.05/30 = 0.0017. (PDF) [file pgen.1011288.s014.pdf]

S4

Category	Trait	P (male)	P (female)
Blood lipids	Apolipoprotein A	3.6e-04	0.045
	Apolipoprotein B	4.6e-07	4.9e-10
	HDL cholesterol	2.5e-09	7.0e-08
	LDL direct	1.5e-08	6.8e-09
	Lipoprotein A	0.484	5.5e-04
	Cholesterol	4.7e-09	3.2e-10
	Triglycerides	8.0e-09	5.1e-20
Hormones	IGF 1	3.3e-05	5.3e-17
	Vitamin D	9.2e-16	1.5e-35
	Bioavailable testosterone	0.275	0.042
	Free testosterone	0.311	0.293
	Testosterone	0.280	0.034
	SHBG	0.264	0.389
Liver markers	Alanine aminotransferase	1.3e-04	2.0e-08
	Albumin	0.040	0.032
	Alkaline phosphatase	0.005	5.8e-08
	Aspartate aminotransferase	0.994	0.030
	C reactive protein	0.602	0.002
	Gamma glutamyltransferase	3.5e-06	5.0e-08
	Direct bilirubin	0.756	0.618
	Total bilirubin	0.716	0.264
	Total protein	0.005	0.154
Kidney markers	Creatinine	1.2e-86	3.0e-61
	Cystatin C	1.7e-19	5.2e-49
	eGFR	0.953	0.094
	HbA1c	0.309	0.065
	Urate	8.3e-10	1.7e-12
	Urea	3.4e-58	9.4e-99
Minerals	Calcium	0.007	0.058
	Phosphate	0.847	0.471

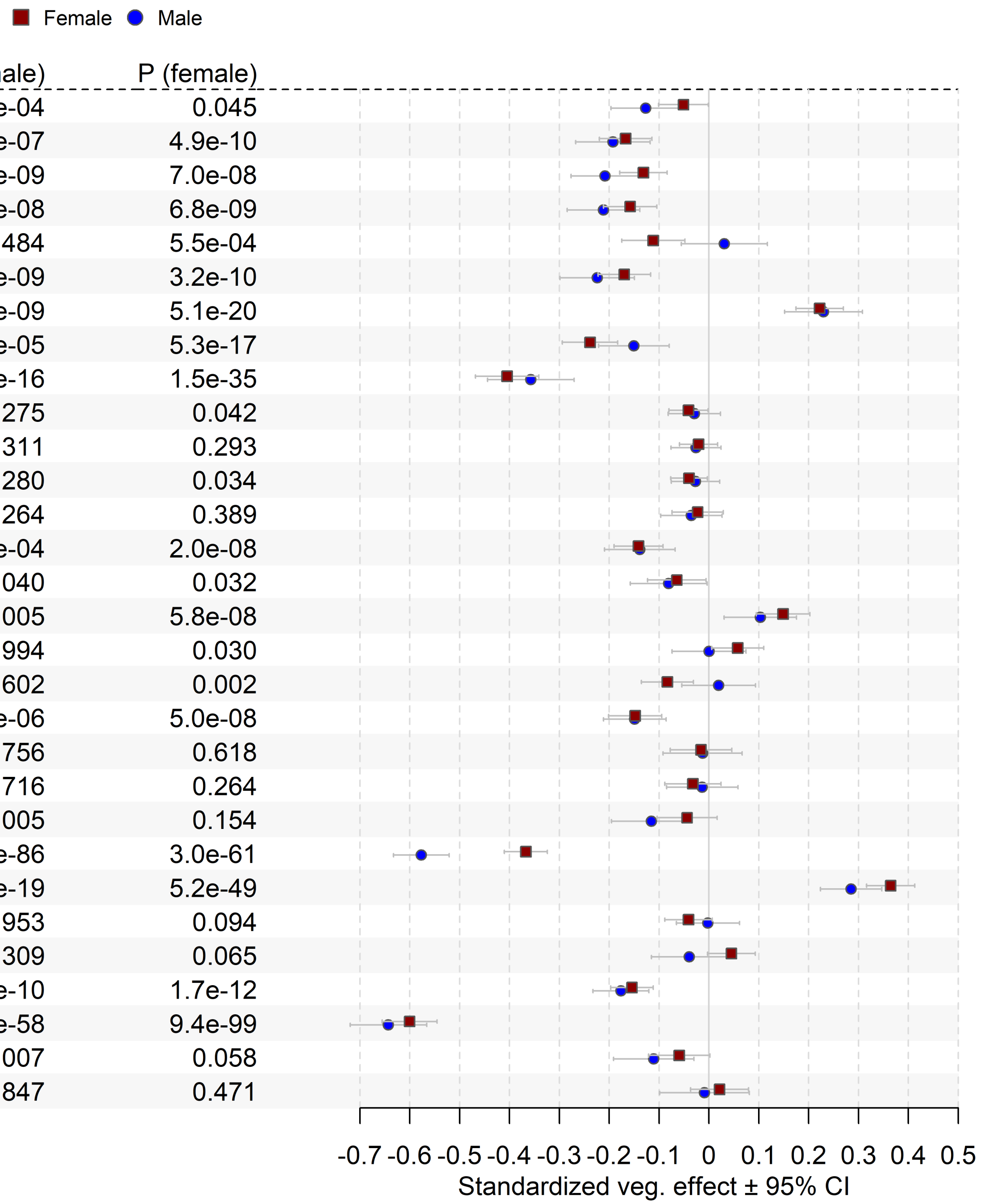

S4 Fig. Sex-stratified forest plot. Effects estimation for vegetarianism in sex-stratified BMI-adjusted model. Error bars indicate 95% confidence interval. Bonferroni-corrected significance threshold at α=0.05/30=0.0017.
