# Supplementary material for: Gene-vegetarianism interactions in calcium, estimated glomerular filtration rate, and testosterone identified in genome-wide analysis across 30 biomarkers
Source: PLoS Genet. 2024 Jul 11;20(7):e1011288. doi: 10.1371/journal.pgen.1011288 (PMC11239071; doi:10.1371/journal.pgen.1011288)
Supplement: S5 Fig — Scatterplot of vegetarian behavior GWAS -log10(P) comparing BMI-adjusted versus. BMI-unadjusted (“standard”) models. Each point represents one variant. Spearman’s Rho (R) and correlation p-value shown. (PDF) [file pgen.1011288.s015.pdf]

S5

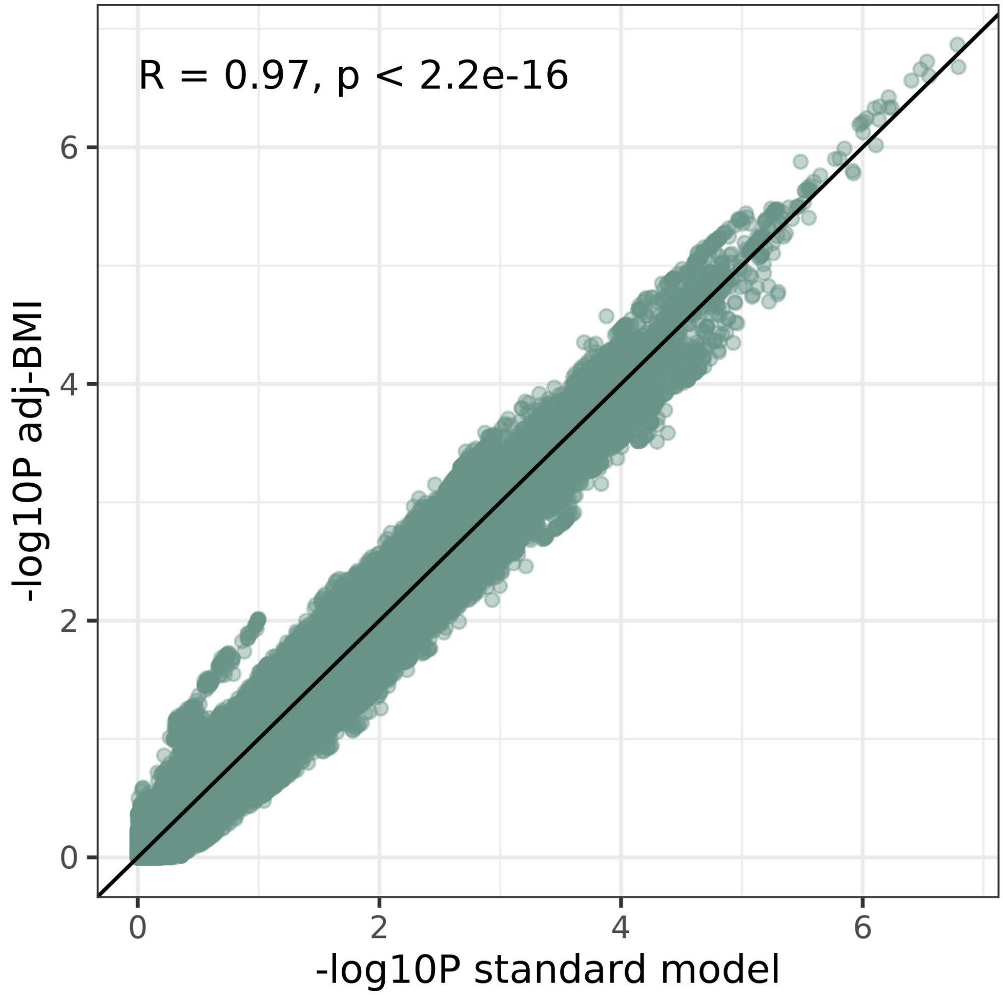

S5 Fig. Correlation plot comparing P-values of BMI-adjusted GWAS model. Scatterplot of vegetarian behavior GWAS -log10(*P*) comparing BMI-adjusted versus BMI-unadjusted (”standard”) models. Each point represents one variant. Spearman’s Rho (R) and correlation *p*-value shown.
