# Supplementary material for: Gene-vegetarianism interactions in calcium, estimated glomerular filtration rate, and testosterone identified in genome-wide analysis across 30 biomarkers
Source: PLoS Genet. 2024 Jul 11;20(7):e1011288. doi: 10.1371/journal.pgen.1011288 (PMC11239071; doi:10.1371/journal.pgen.1011288)
Supplement: S6 Fig — Manhattan plots and quantile-quantile (QQ) plots showing -log10(P) of genetic effects with vegetarianism behavior. Genomic control (λ) for each model is shown in QQ plots. Plots correspond to: (a) Variant-level GWAS and (b) gene-level GWAS, where P-values were aggregated by MAGMA. Top variants with P<1×10^−6^ are annotated. Top genes (P<1×10^−4^) in a 5 Mb window were annotated. No variants or genes for vegetarianism behavior were genome-wide significant. All plots shown with and without inclusion of BMI as a covariate. (PDF) [file pgen.1011288.s016.pdf]

S6a

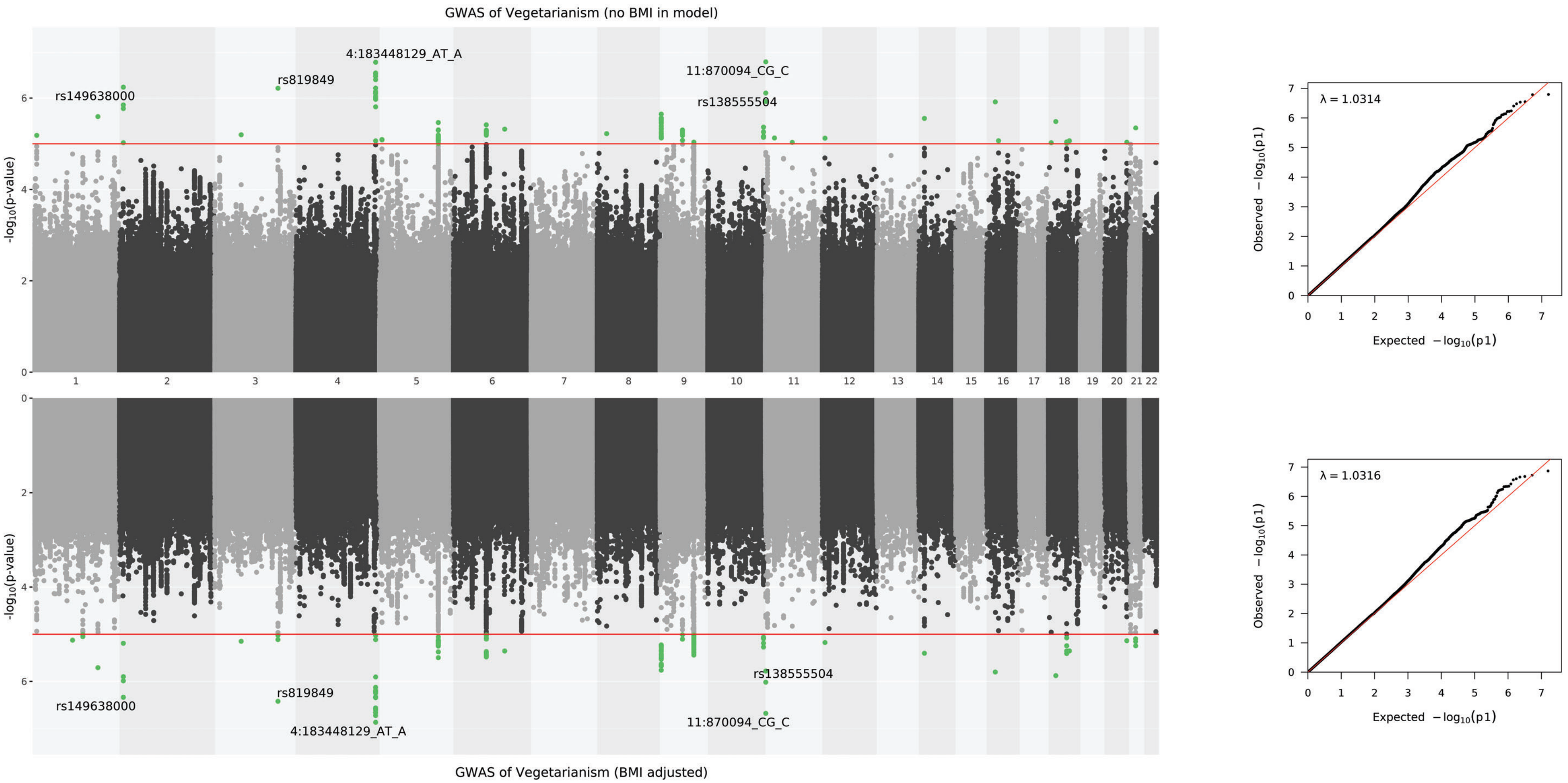

GWAS of Vegetarianism (no BMI in model)
-log10(p-value)
rs149638000
rs819849
4:183448129_AT_A
11:870094_CG_C
rs138555504
λ = 1.0314
Observed −log10(p1)
Expected −log10(p1)
GWAS of Vegetarianism (BMI adjusted)
λ = 1.0316

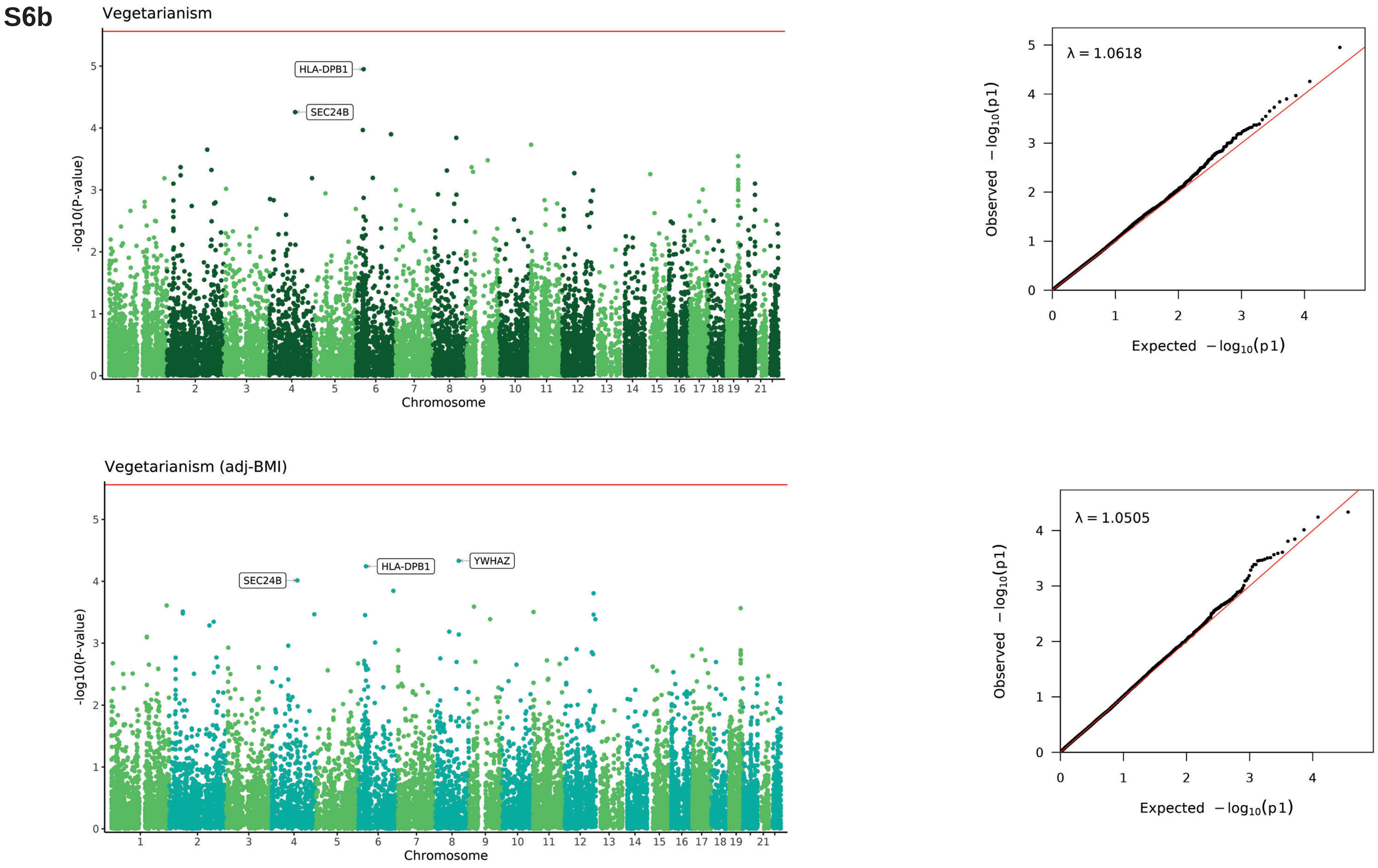

S6 Fig. Vegetarianism genome-wide association Manhattan and QQ plots. Manhattan plots and quantile-quantile (QQ) plots showing −log10(*P*) of genetic effects with vegetarianism behavior. Genomic control (λ) for each model is shown in QQ plots. Plots correspond to **(a)** Variant-level GWAS and **(b)** gene-level GWAS, where *P*-values were aggregated by MAGMA. Top variants with $P<1\times10^{-6}$ are annotated. Top genes ($P<1\times10^{-4}$) in a 5 Mb window were annotated. No variants or genes for vegetarianism behavior were genome-wide significant. All plots shown with and without inclusion of BMI as a covariate.
