# Supplementary material for: Gene-vegetarianism interactions in calcium, estimated glomerular filtration rate, and testosterone identified in genome-wide analysis across 30 biomarkers
Source: PLoS Genet. 2024 Jul 11;20(7):e1011288. doi: 10.1371/journal.pgen.1011288 (PMC11239071; doi:10.1371/journal.pgen.1011288)
Supplement: S7 Fig — Manhattan plots and QQ plots showing the variant-level −log10(P) of genome-wide gene-vegetarianism interaction effects in thirty serum biomarker traits. The blue line corresponds to the genome-wide suggestive threshold (P<1×10^–5^). In the standard interaction model (a), one trait, calcium, had a significant variant above the genome-wide significance threshold (P<5×10^–8^; red line). (b) No variants were significant in the BMI-adjusted model. (PDF) [file pgen.1011288.s017.pdf]

S7a

Alanine aminotransferase

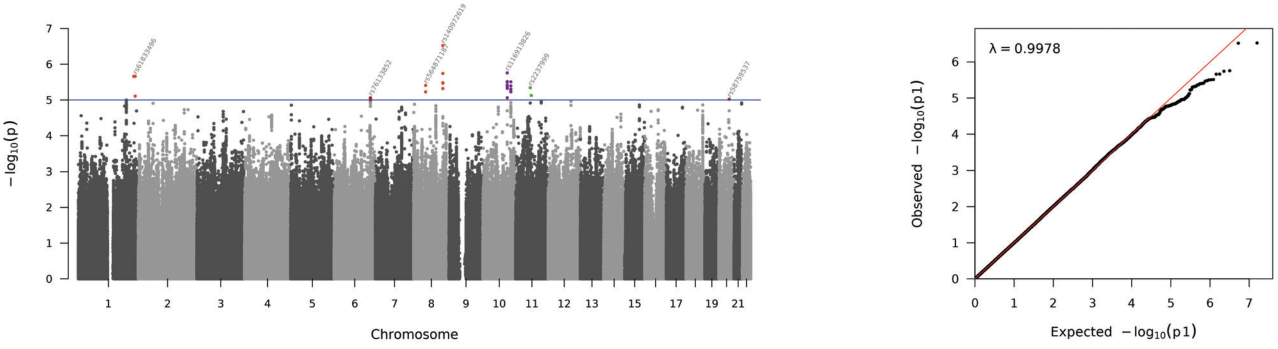

Albumin

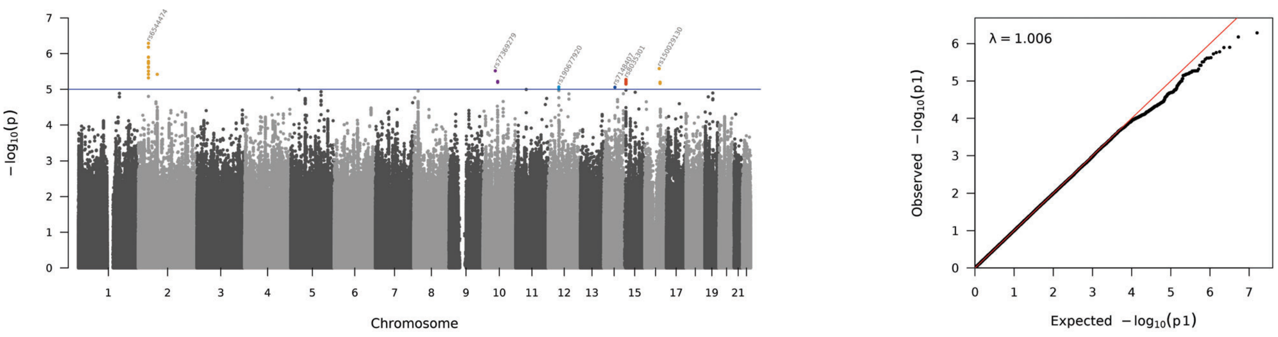

Alkaline phosphatase

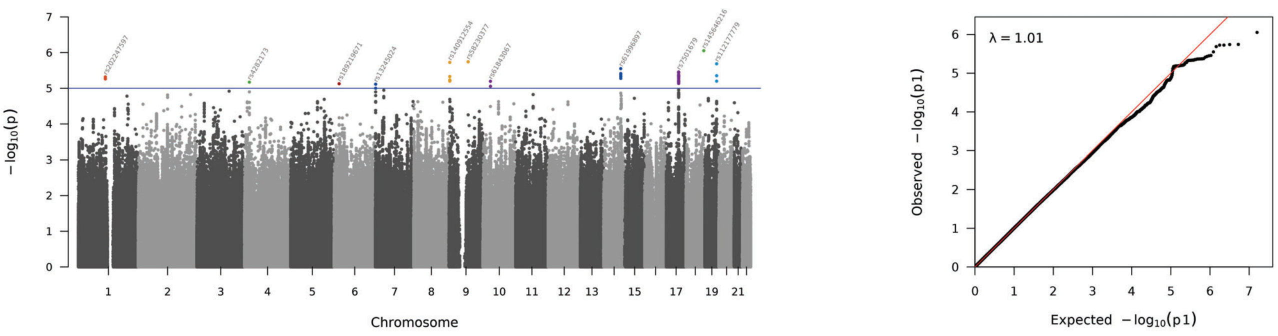

Apolipoprotein A

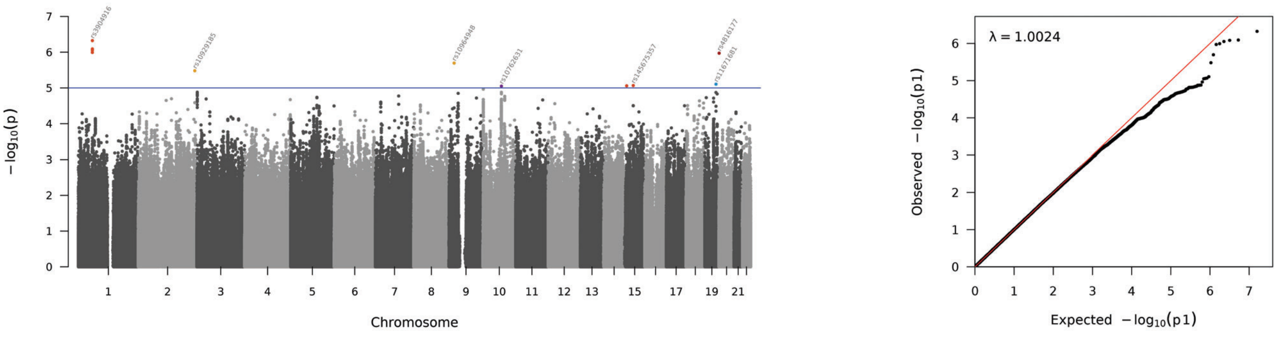

S7a

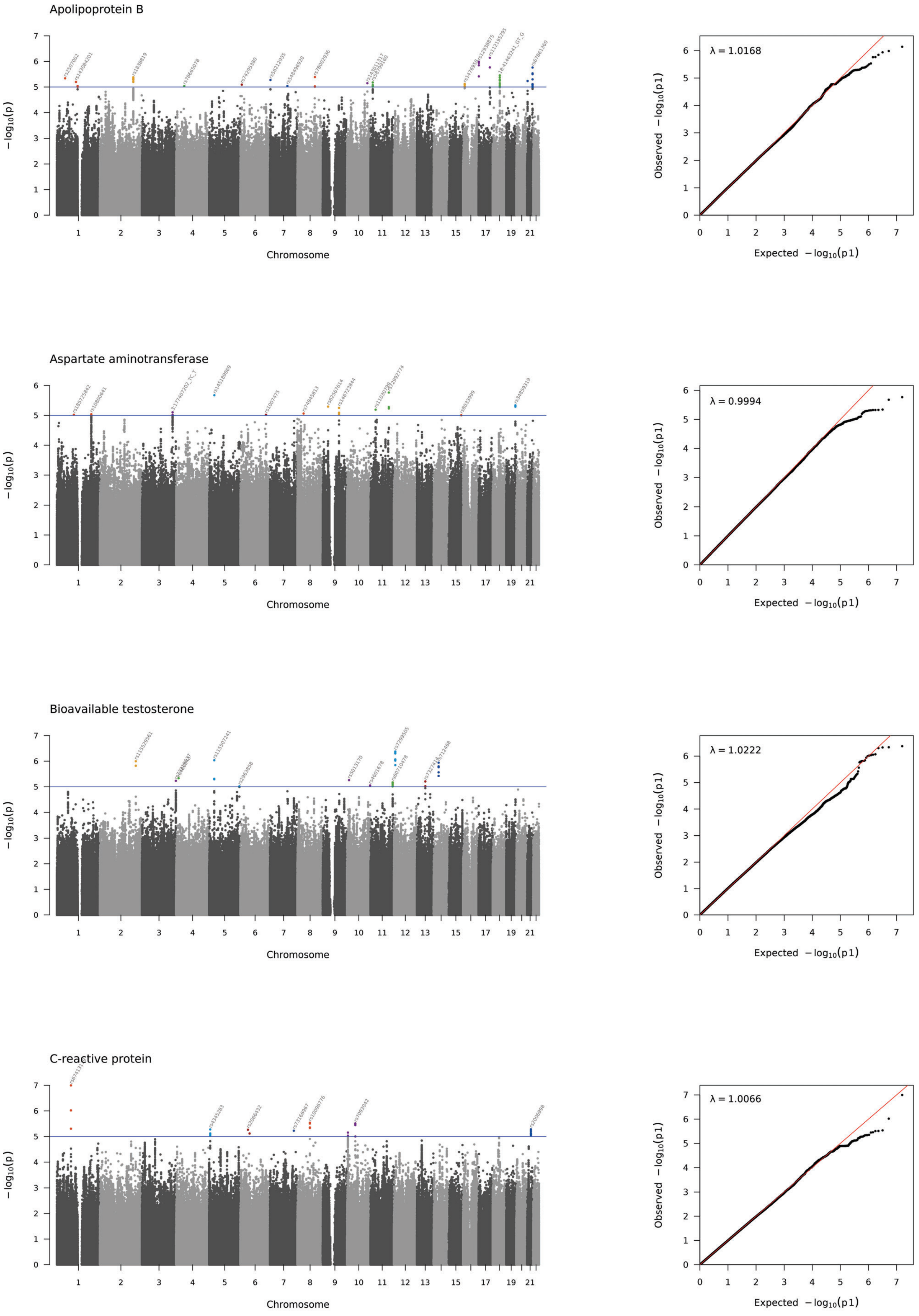

Apolipoprotein B
λ = 1.0168
Aspartate aminotransferase
λ = 0.9994
Bioavailable testosterone
λ = 1.0222
C-reactive protein
λ = 1.0066
Chromosome
−log10(p)
Expected −log10(p1)
Observed −log10(p1)

S7a

Calcium

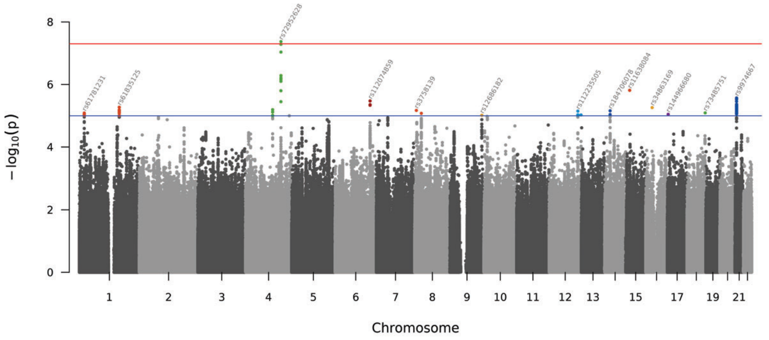

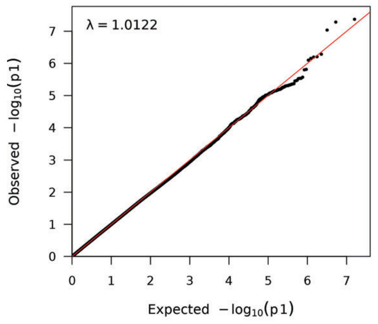

Cholesterol

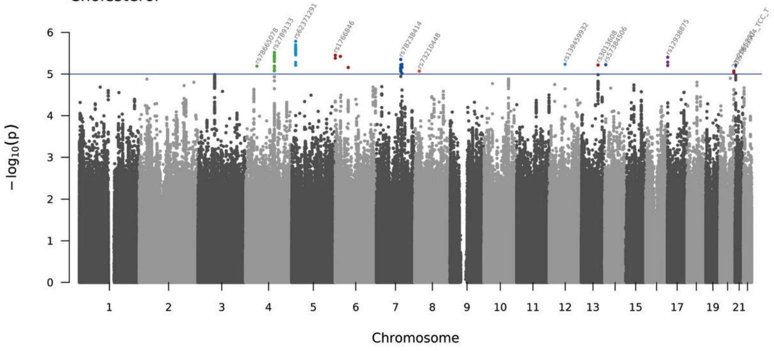

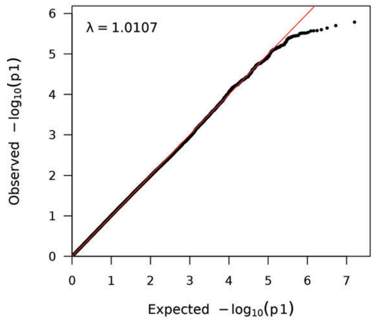

Creatinine

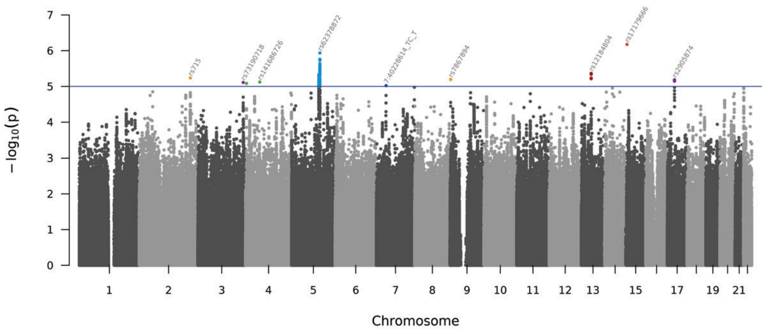

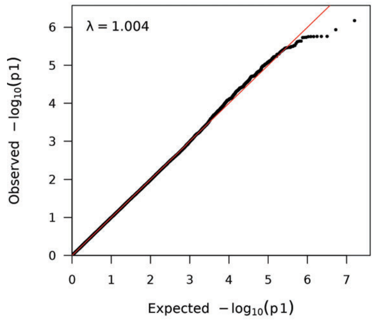

Cystatin C

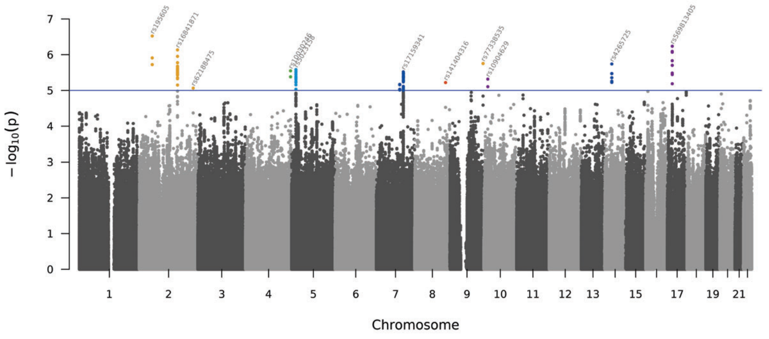

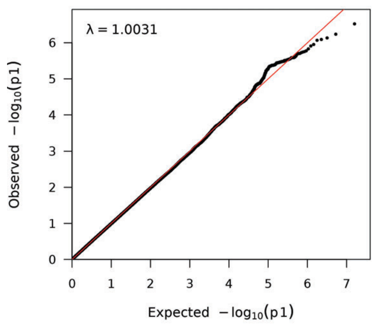

S7a

Direct bilirubin

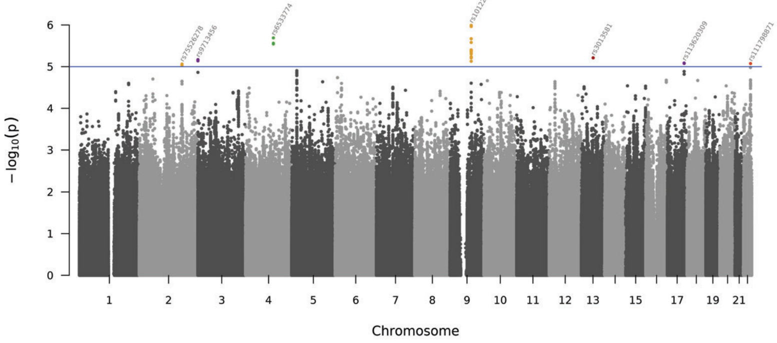

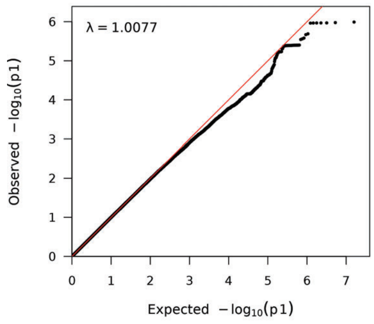

eGFR

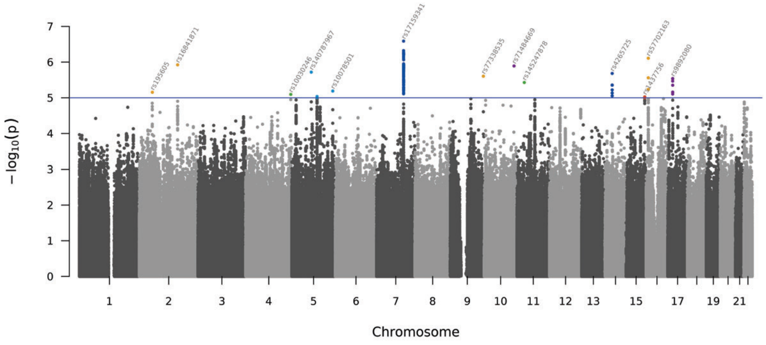

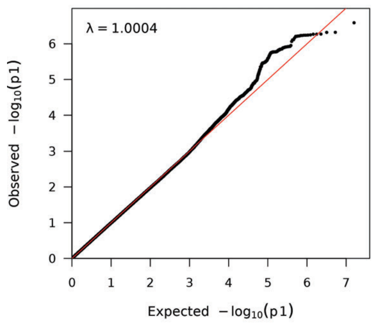

Free testosterone

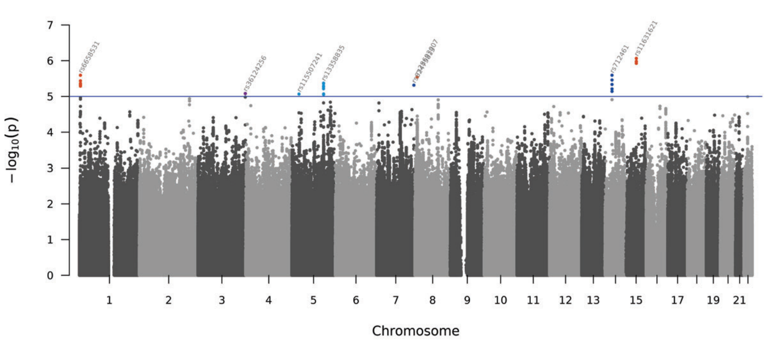

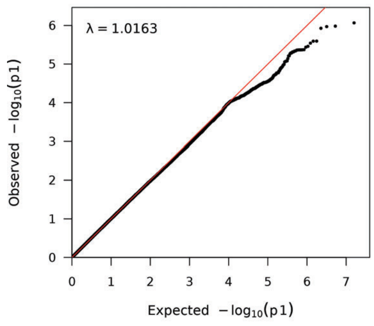

Gamma glutamyltransferase

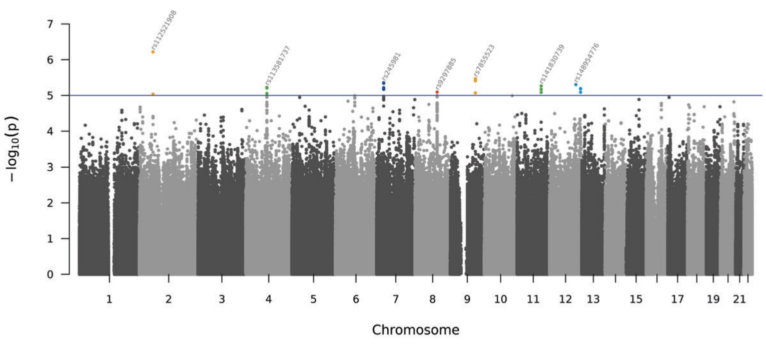

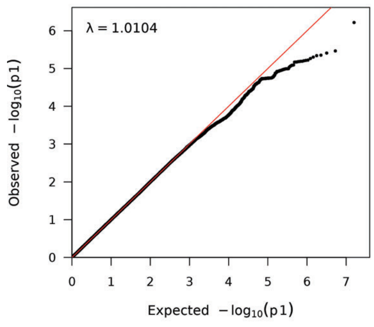

S7a

HbA1c

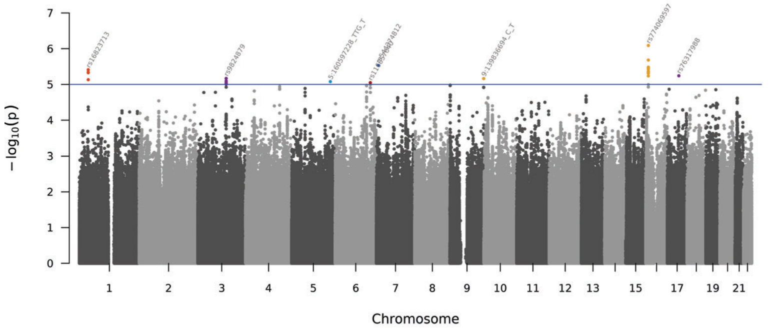

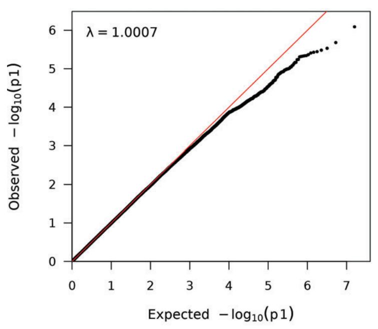

HDL cholesterol

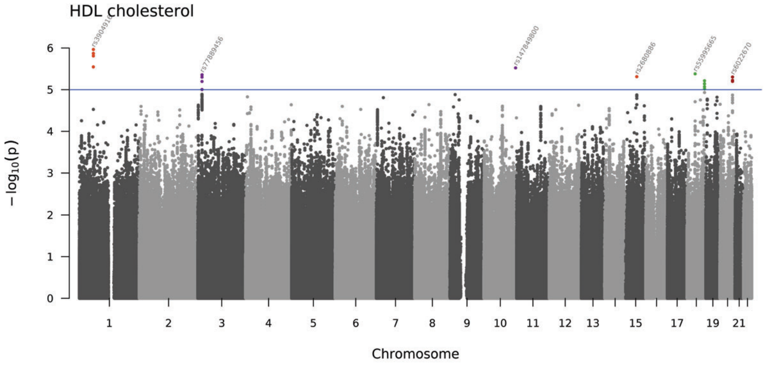

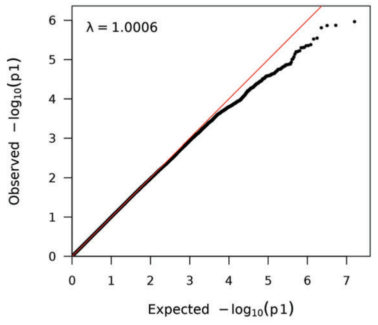

IGF 1

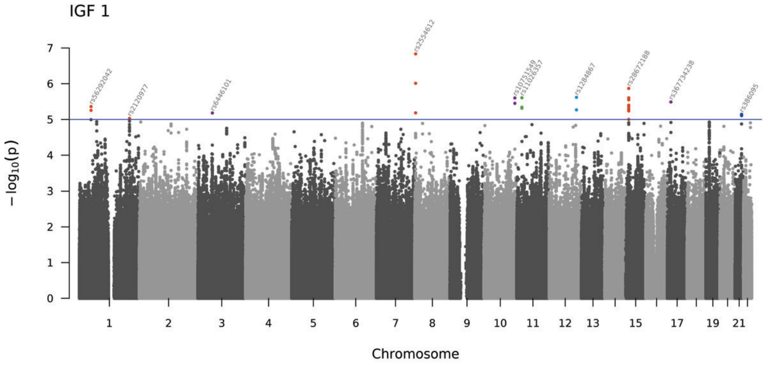

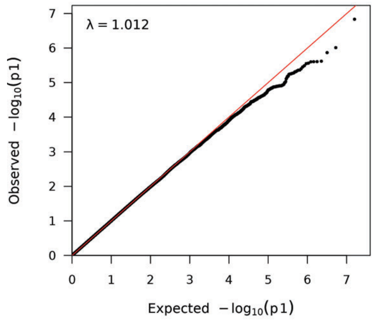

LDL direct

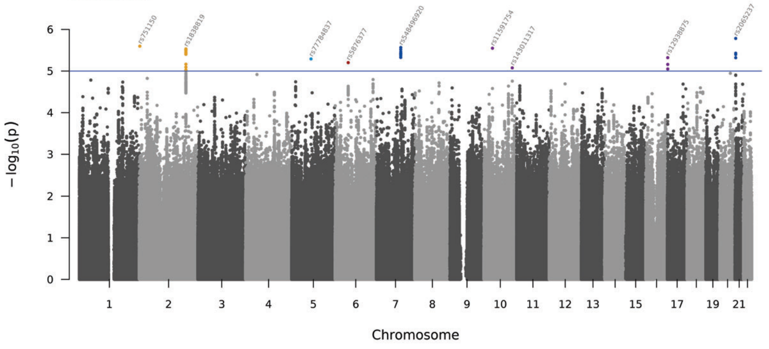

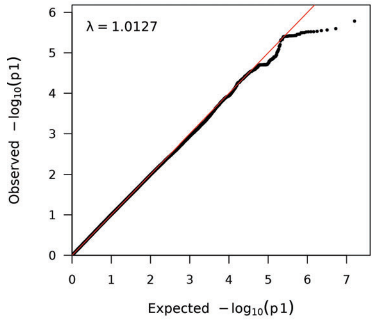

S7a

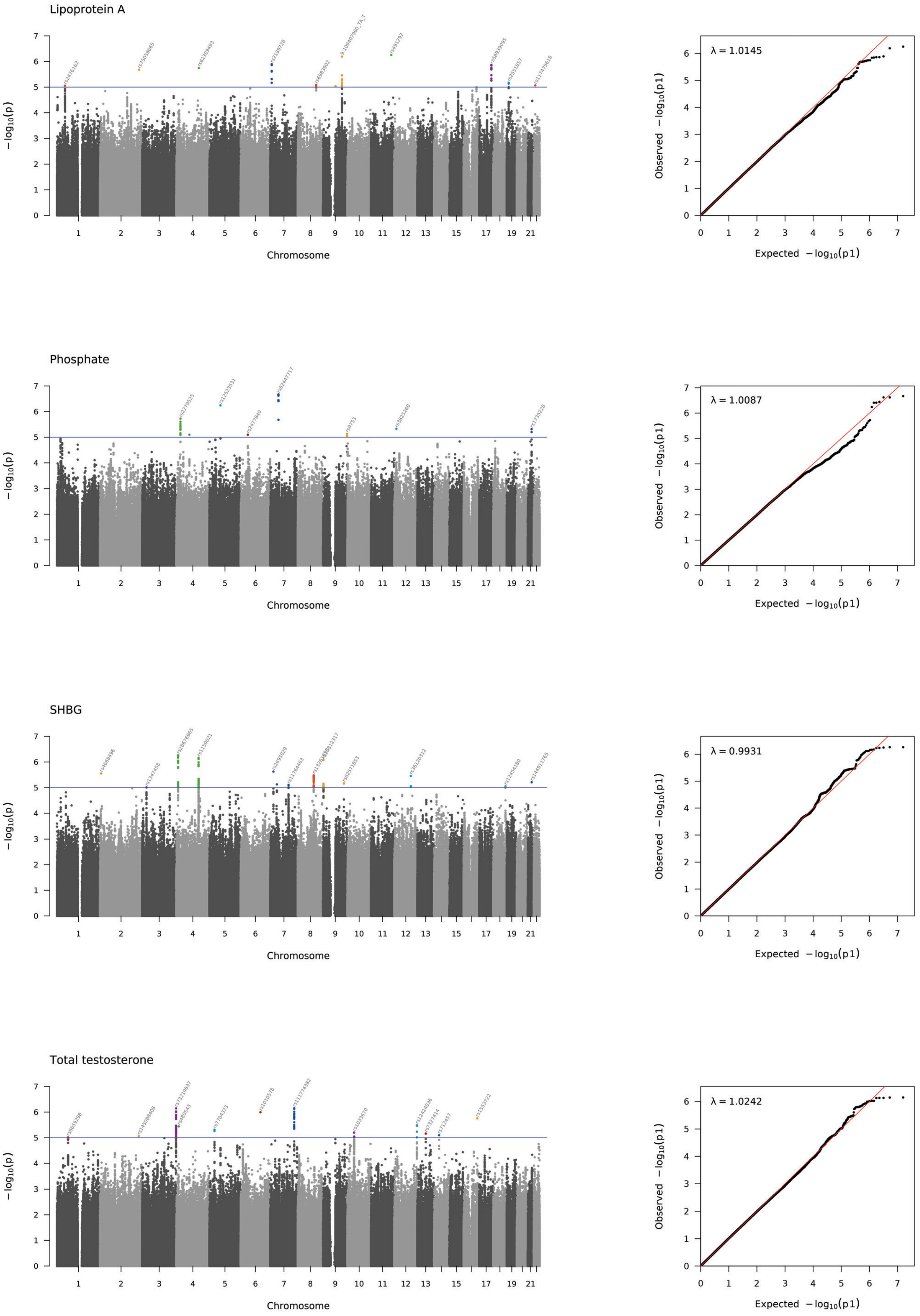

Lipoprotein A
Phosphate
SHBG
Total testosterone
Chromosome
−log10(p)
λ = 1.0145
λ = 1.0087
λ = 0.9931
λ = 1.0242
Observed −log10(p1)
Expected −log10(p1)

S7a

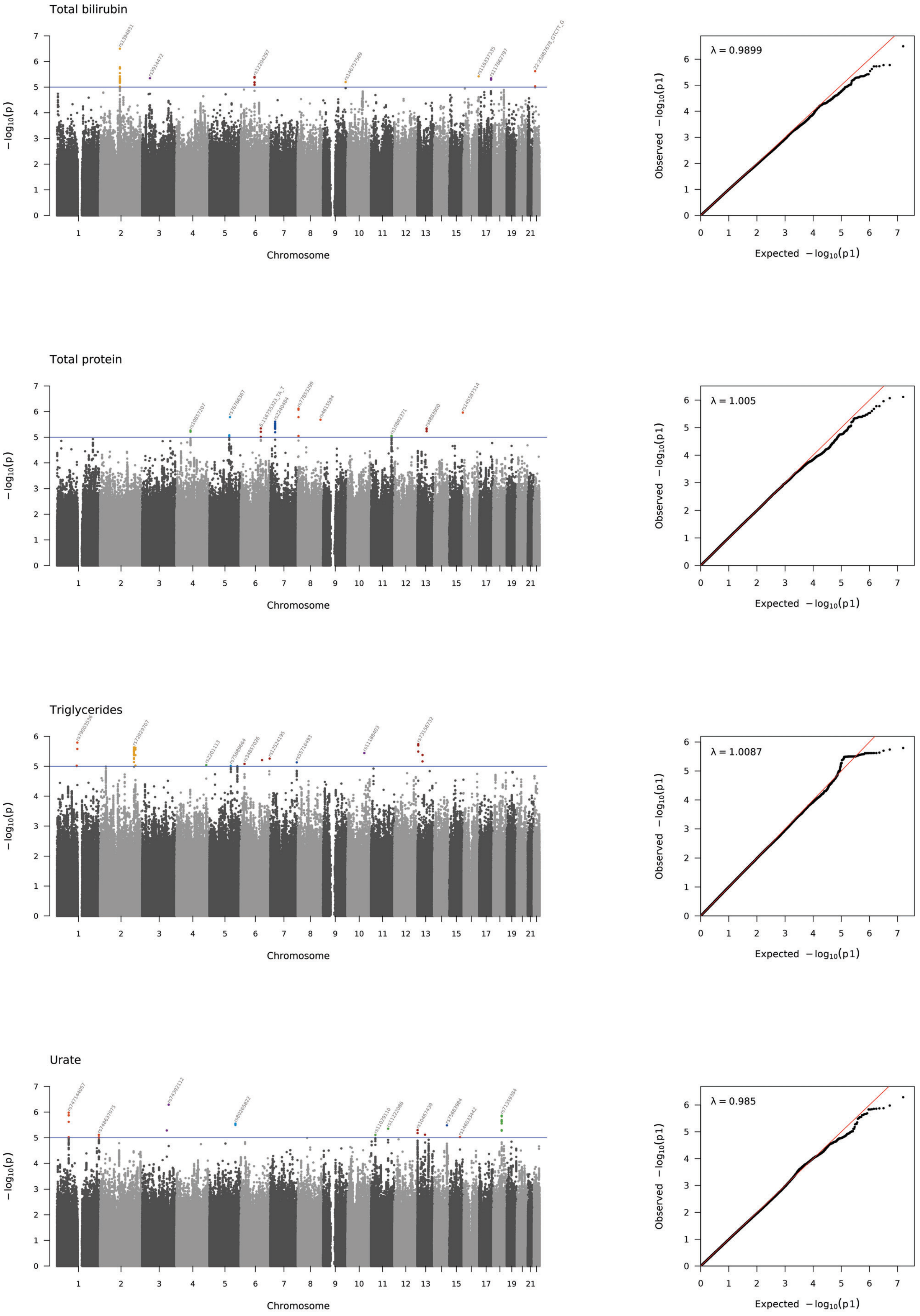
Total bilirubin
−log10(p)
Chromosome
λ = 0.9899
Observed −log10(p1)
Expected −log10(p1)
Total protein
−log10(p)
Chromosome
λ = 1.005
Observed −log10(p1)
Expected −log10(p1)
Triglycerides
−log10(p)
Chromosome
λ = 1.0087
Observed −log10(p1)
Expected −log10(p1)
Urate
−log10(p)
Chromosome
λ = 0.985
Observed −log10(p1)
Expected −log10(p1)

S7a

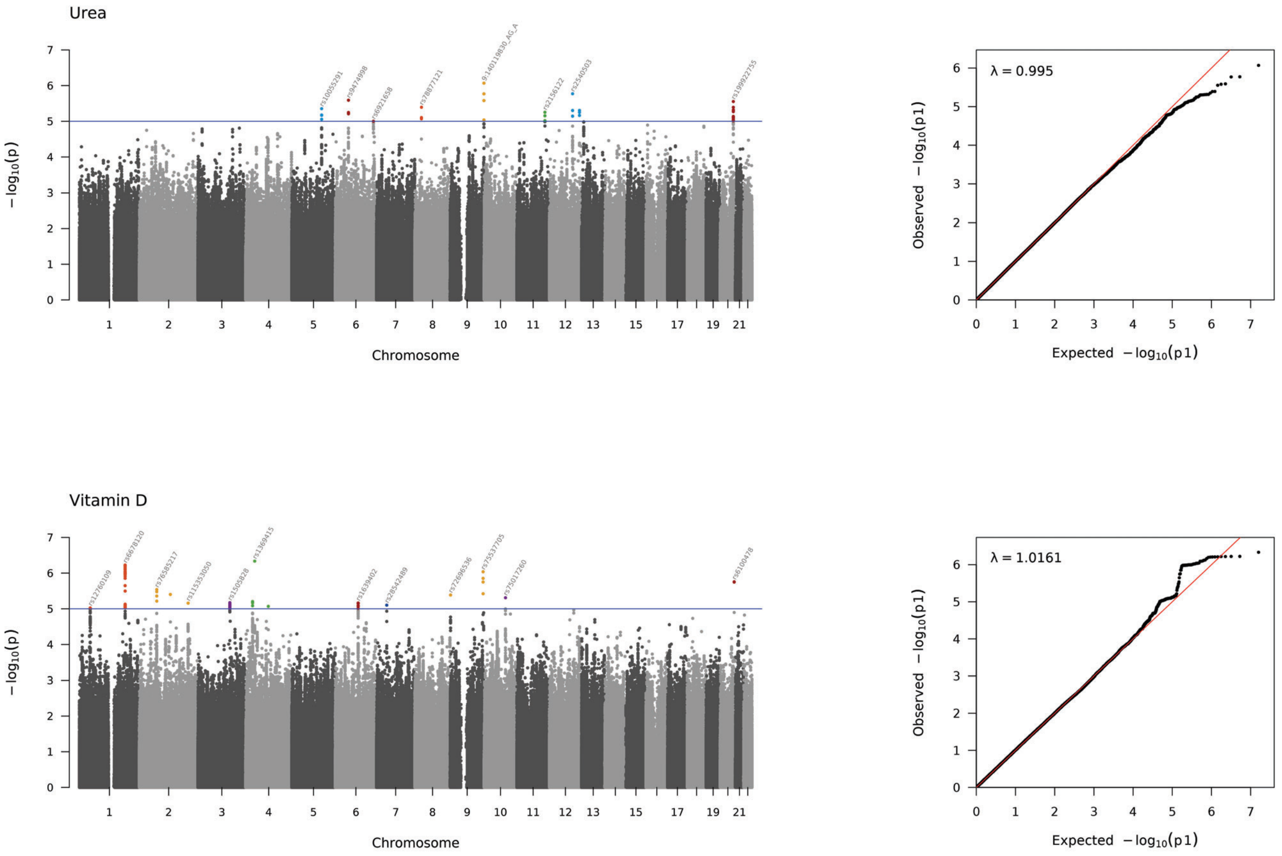
Urea
−log10(p)
Chromosome
λ = 0.995
Observed −log10(p1)
Expected −log10(p1)
Vitamin D
−log10(p)
Chromosome
λ = 1.0161
Observed −log10(p1)
Expected −log10(p1)

S7b

Alanine aminotransferase (adj-BMI)

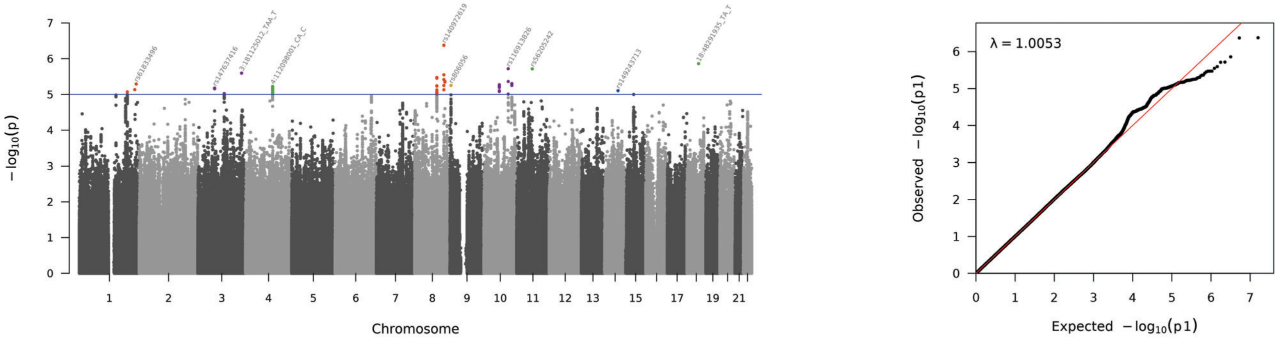

Albumin (adj-BMI)

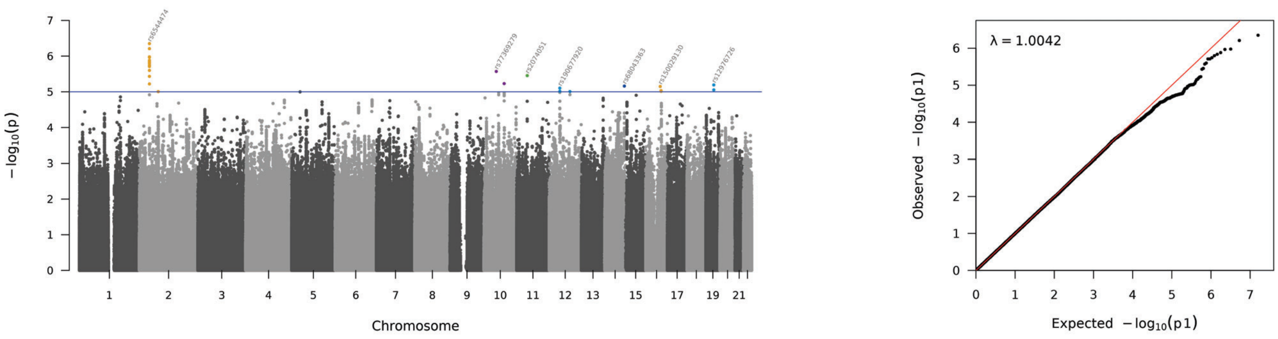

Alkaline phosphatase (adj-BMI)

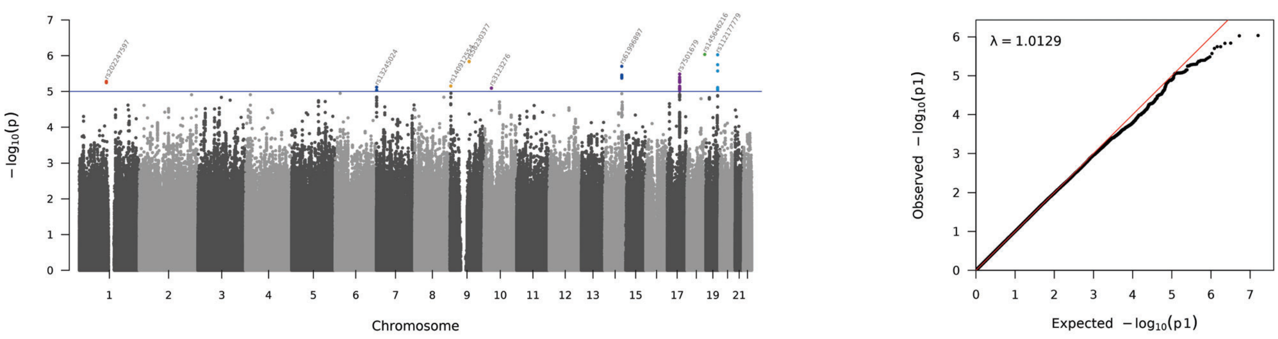

Apolipoprotein A (adj-BMI)

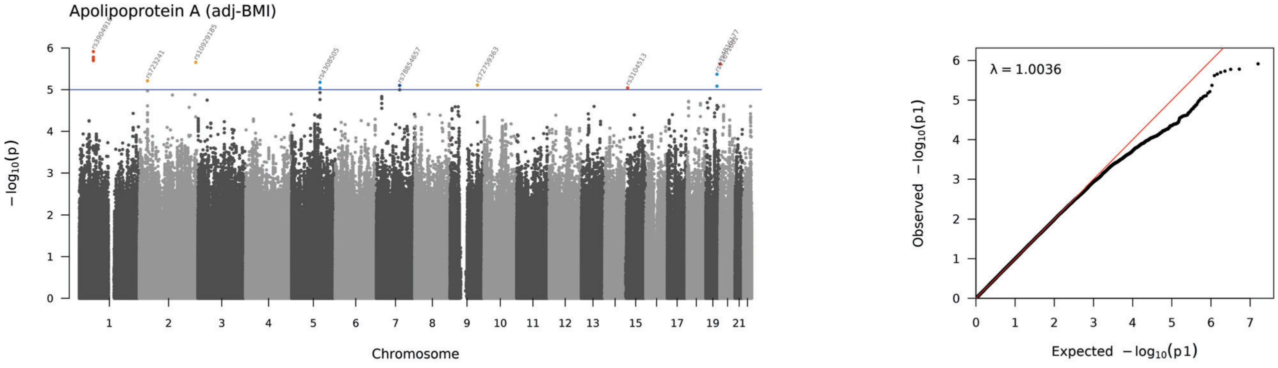

S7b

Apolipoprotein B (adj-BMI)

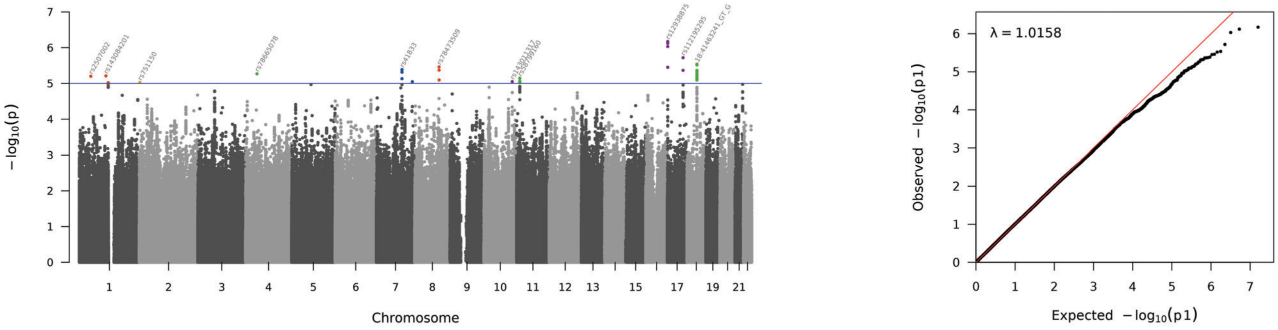

Aspartate aminotransferase (adj-BMI)

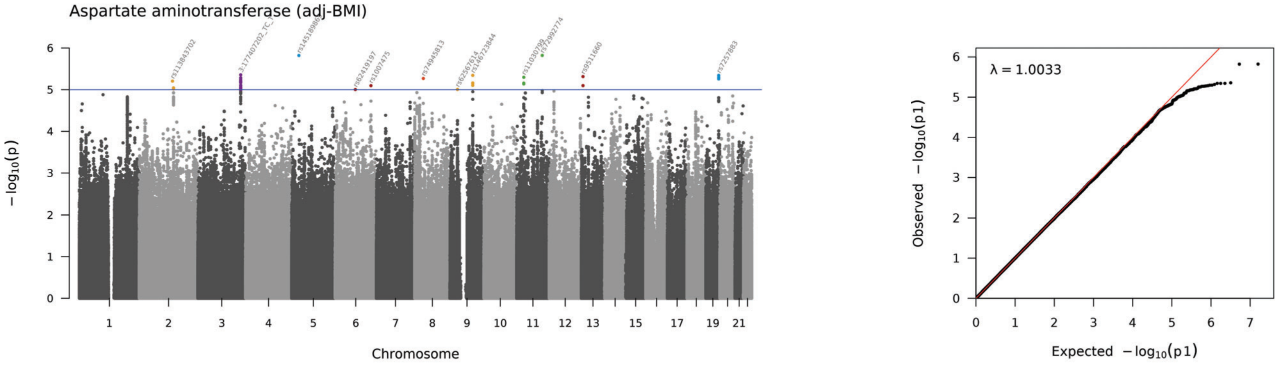

Bioavailable testosterone (adj-BMI)

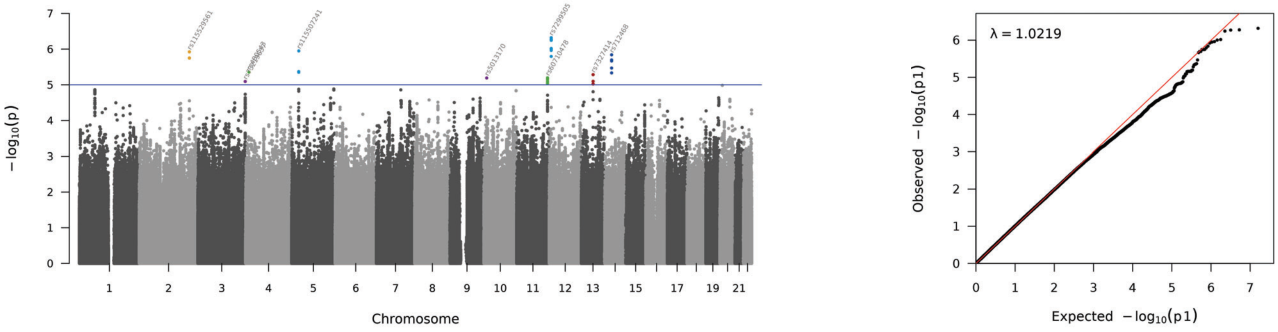

C-reactive protein (adj-BMI)

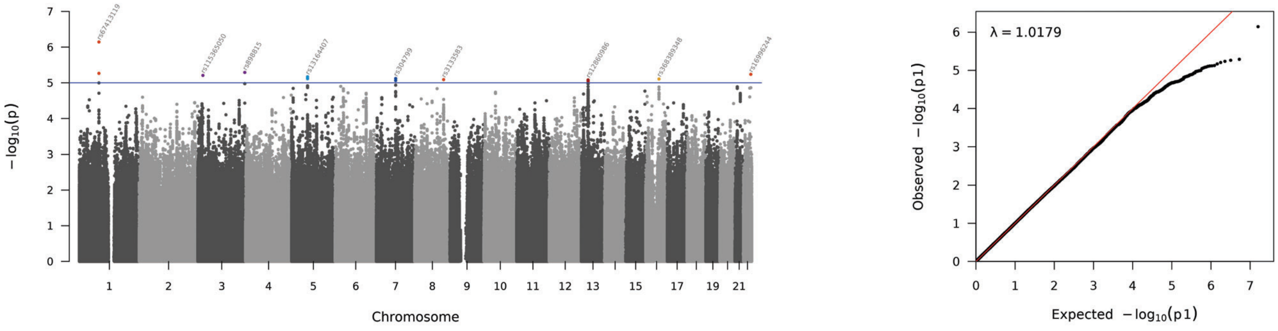

S7b

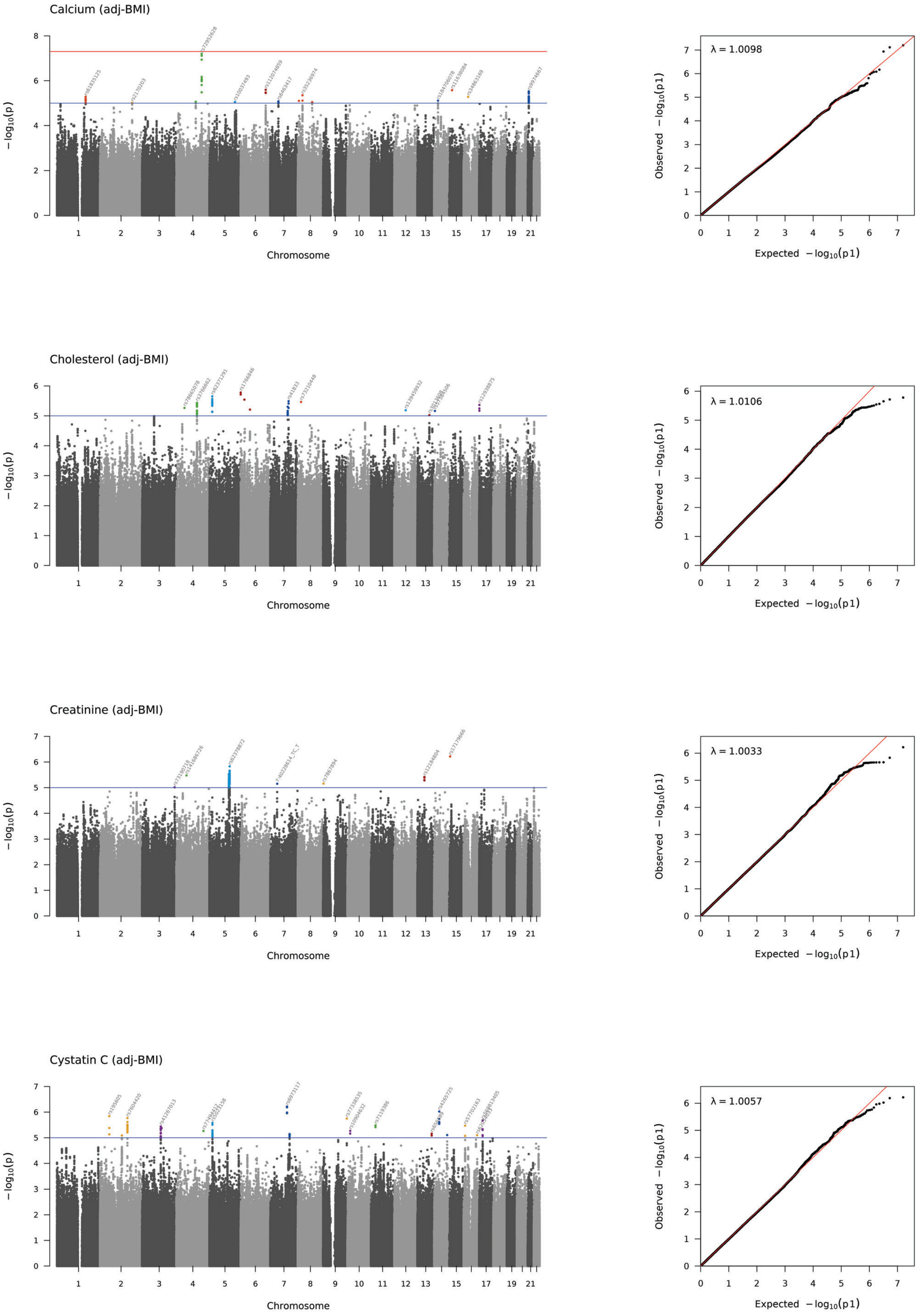
Calcium (adj-BMI)
λ = 1.0098
Cholesterol (adj-BMI)
λ = 1.0106
Creatinine (adj-BMI)
λ = 1.0033
Cystatin C (adj-BMI)
λ = 1.0057
− log10(p)
Chromosome
Observed − log10(p1)
Expected − log10(p1)

S7b

Direct bilirubin (adj-BMI)

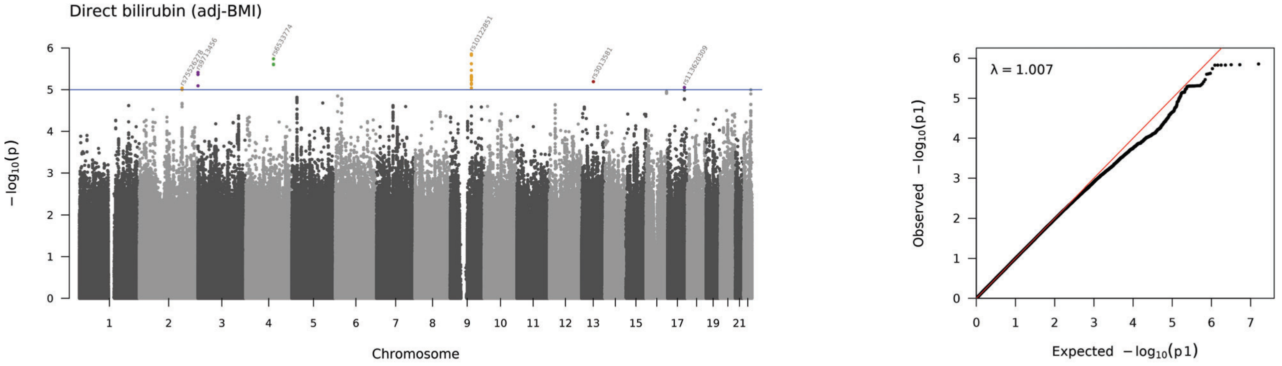

eGFR (adj-BMI)

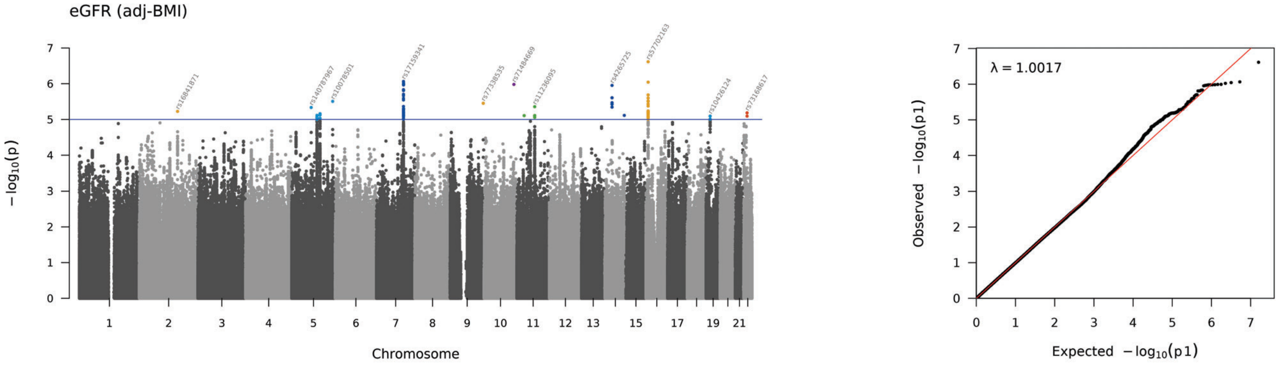

Free testosterone (adj-BMI)

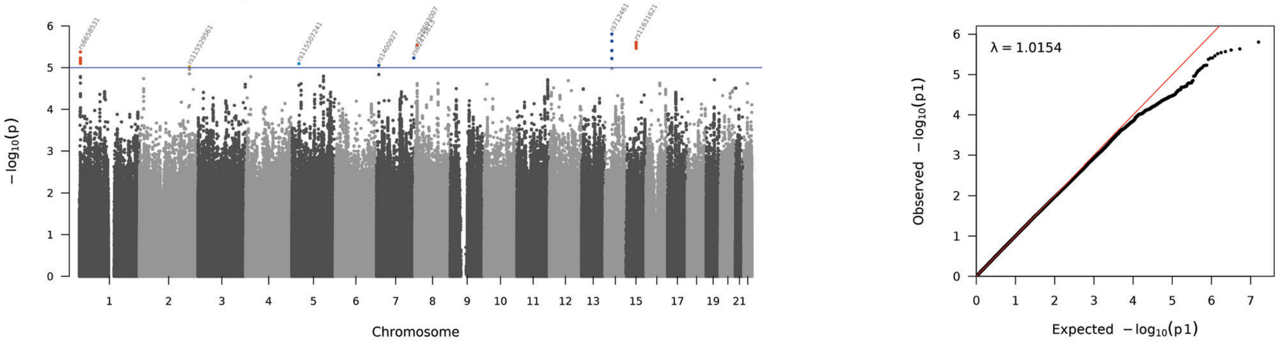

Gamma glutamyltransferase (adj-BMI)

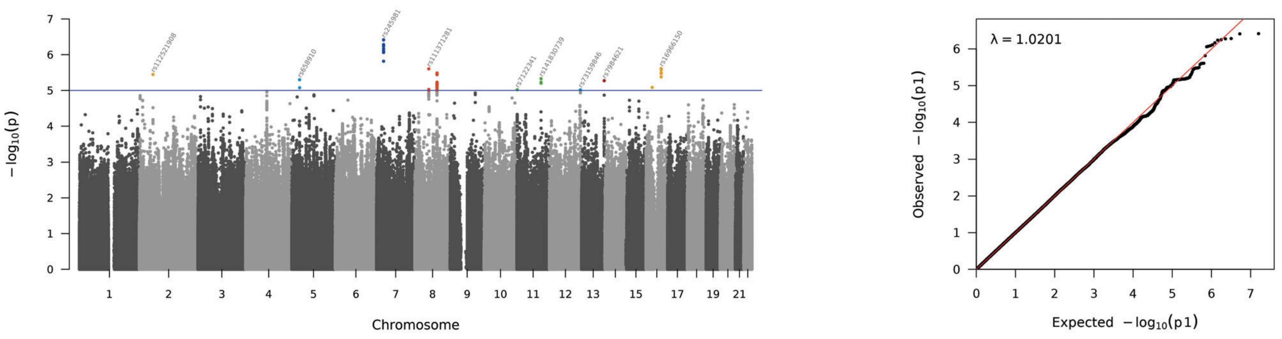

S7b

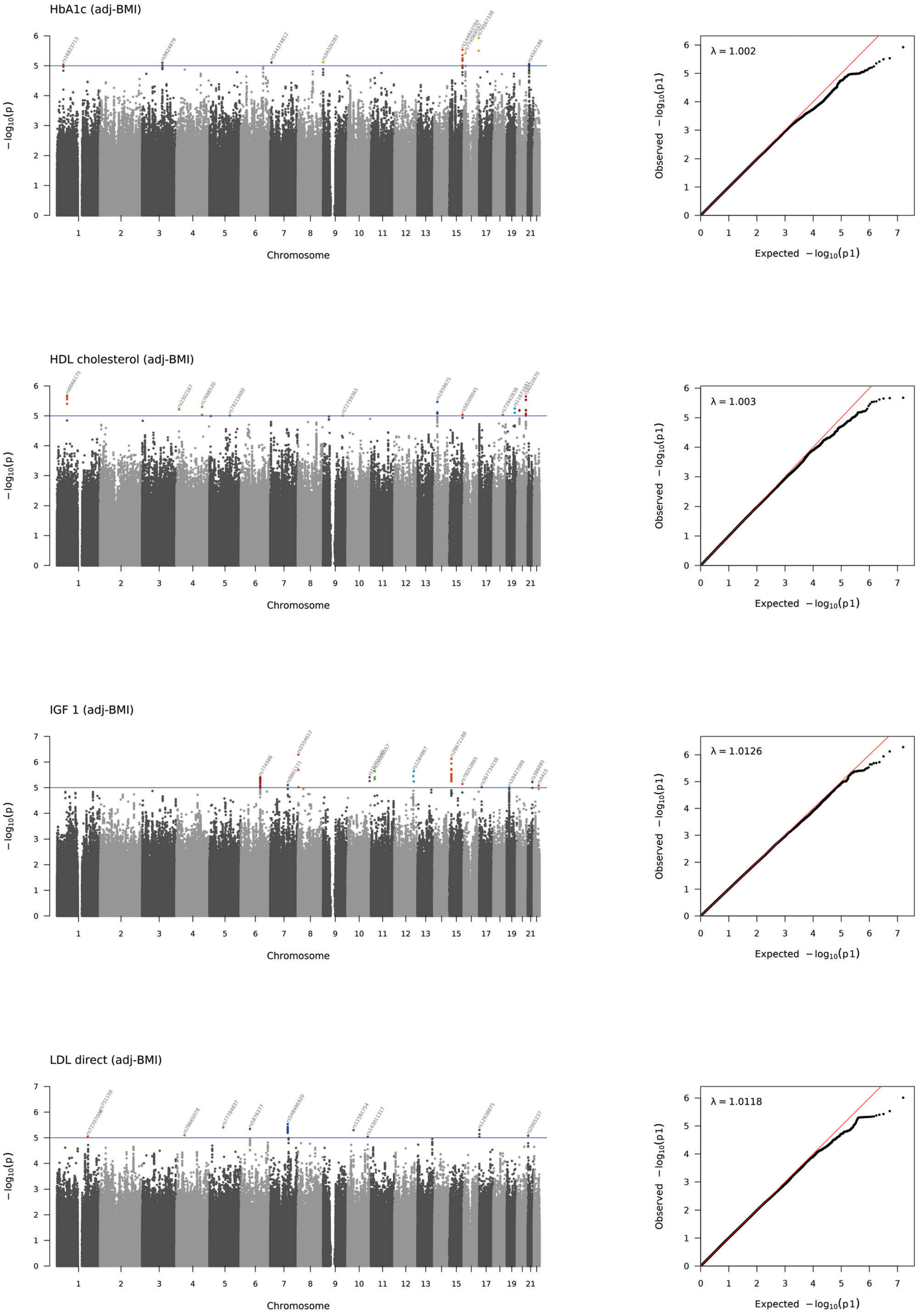
HbA1c (adj-BMI)
λ = 1.002
HDL cholesterol (adj-BMI)
λ = 1.003
IGF 1 (adj-BMI)
λ = 1.0126
LDL direct (adj-BMI)
λ = 1.0118
− log10(p)
Chromosome
Observed − log10(p1)
Expected − log10(p1)

S7b

Lipoprotein A (adj-BMI)

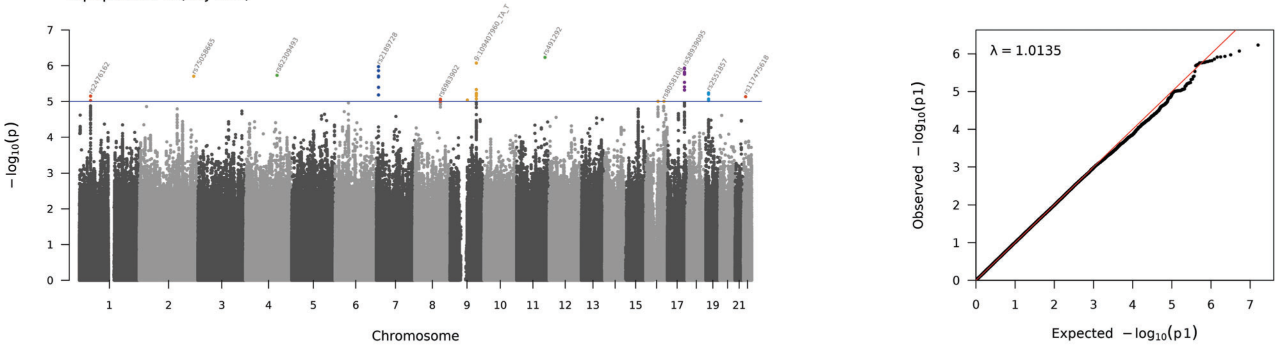

Phosphate (adj-BMI)

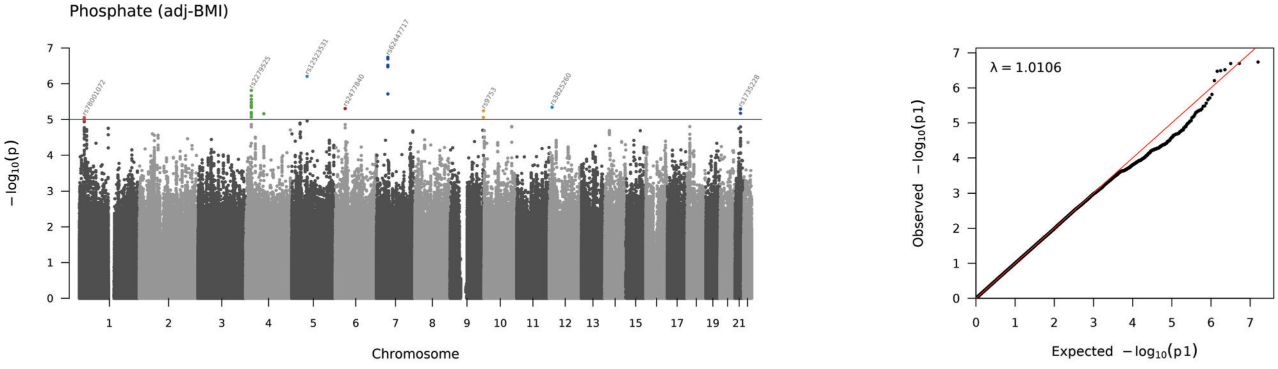

SHBG (adj-BMI)

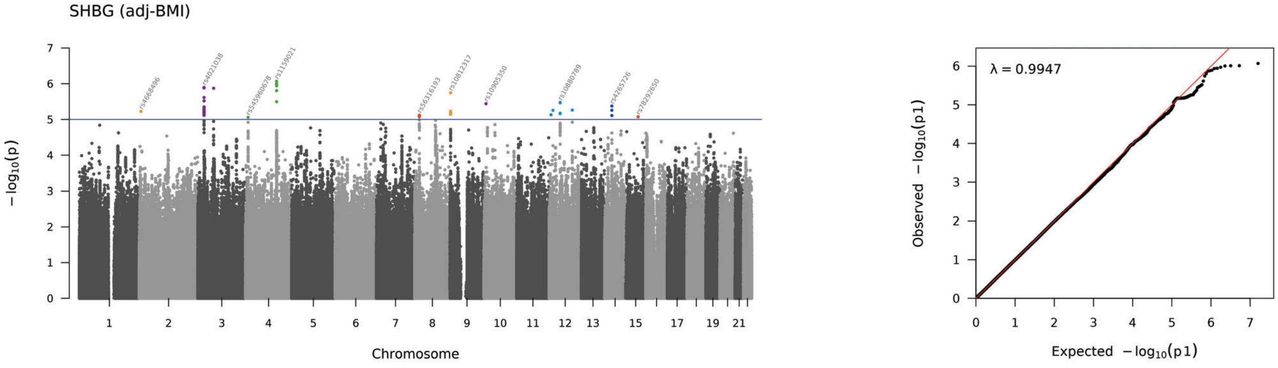

Total testosterone (adj-BMI)

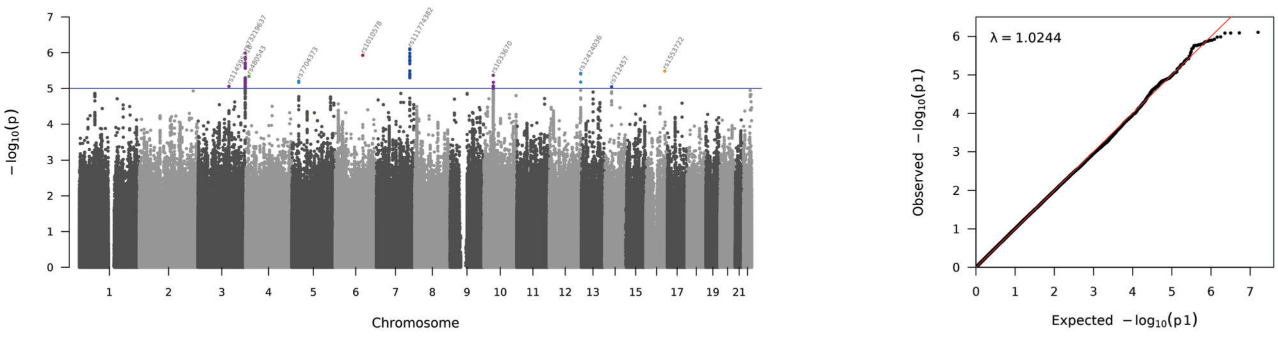

S7b

Total bilirubin (adj-BMI)

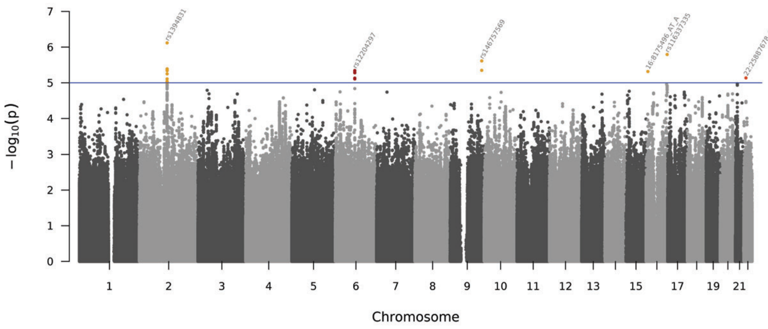

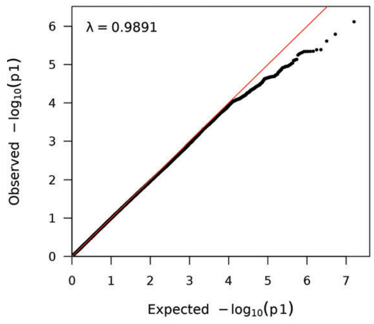

Total protein (adj-BMI)

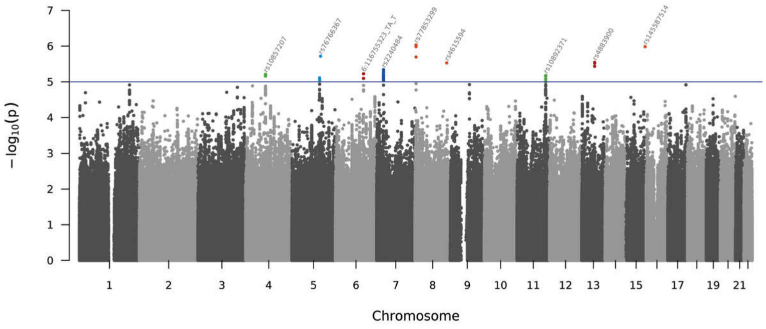

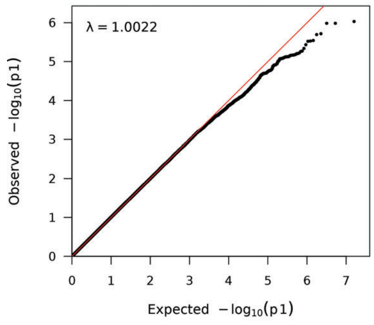

Triglycerides (adj-BMI)

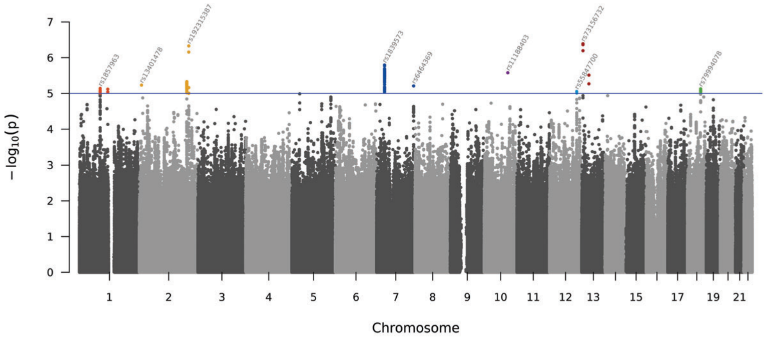

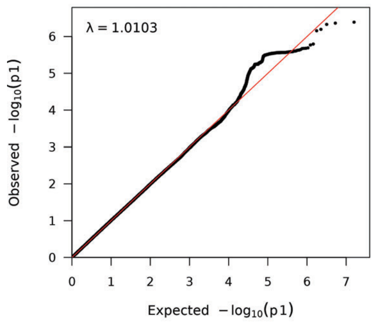

Urate (adj-BMI)

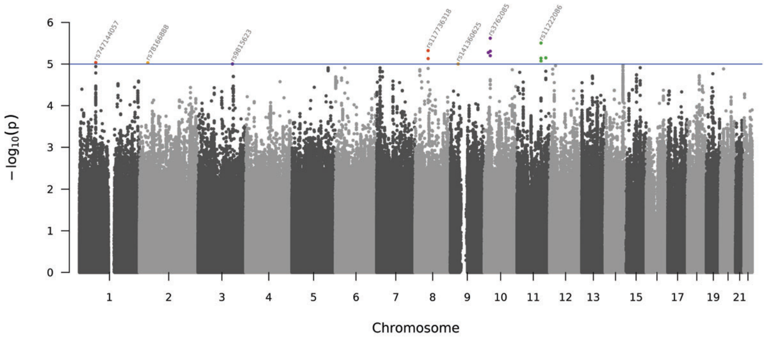

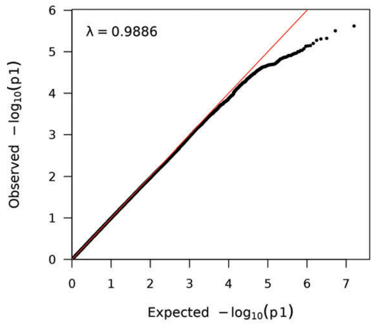

S7b

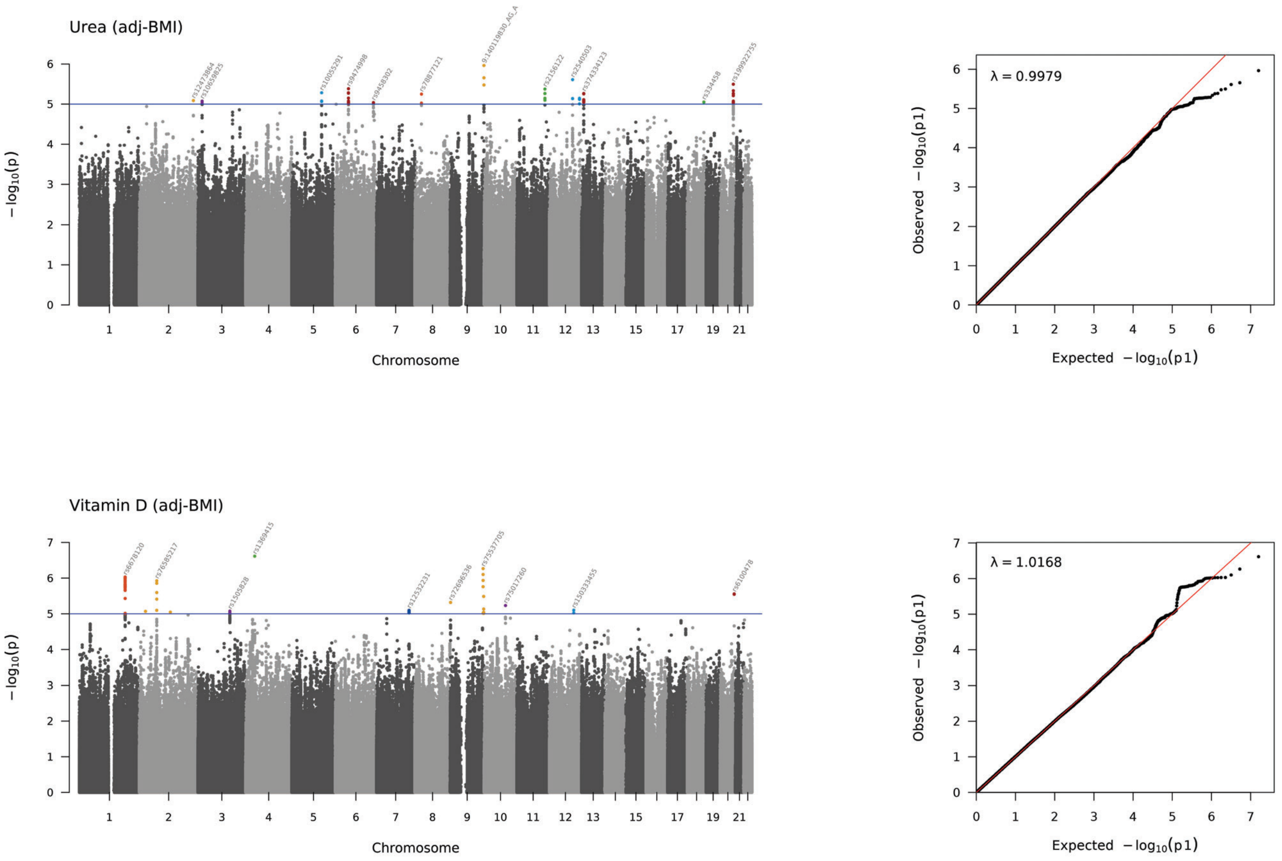

S7 Fig. Variant-level gene-vegetarianism interaction Manhattan plots. Manhattan plots and QQ plots showing the variant-level −log10(*P*) of genome-wide gene-vegetarianism interaction effects in thirty serum biomarker traits. The blue line corresponds to the genome-wide suggestive threshold ($P<1\times10^{-5}$). In the standard interaction model **(a)**, one trait, calcium, had a significant variant above the genome-wide significance threshold ($P<5\times10^{-8}$; red line). **(b)** No variants were significant in the BMI-adjusted model.
