# Supplementary material for: Gene-vegetarianism interactions in calcium, estimated glomerular filtration rate, and testosterone identified in genome-wide analysis across 30 biomarkers
Source: PLoS Genet. 2024 Jul 11;20(7):e1011288. doi: 10.1371/journal.pgen.1011288 (PMC11239071; doi:10.1371/journal.pgen.1011288)
Supplement: S8 Fig — Bars show bin counts of interaction P-values from males and females for all interactions reaching suggestive significance in the full cohort genome-wide interaction analysis. Sex-stratified models were also run with and without BMI adjustment, as in the full cohort. (PDF) [file pgen.1011288.s018.pdf]

S8

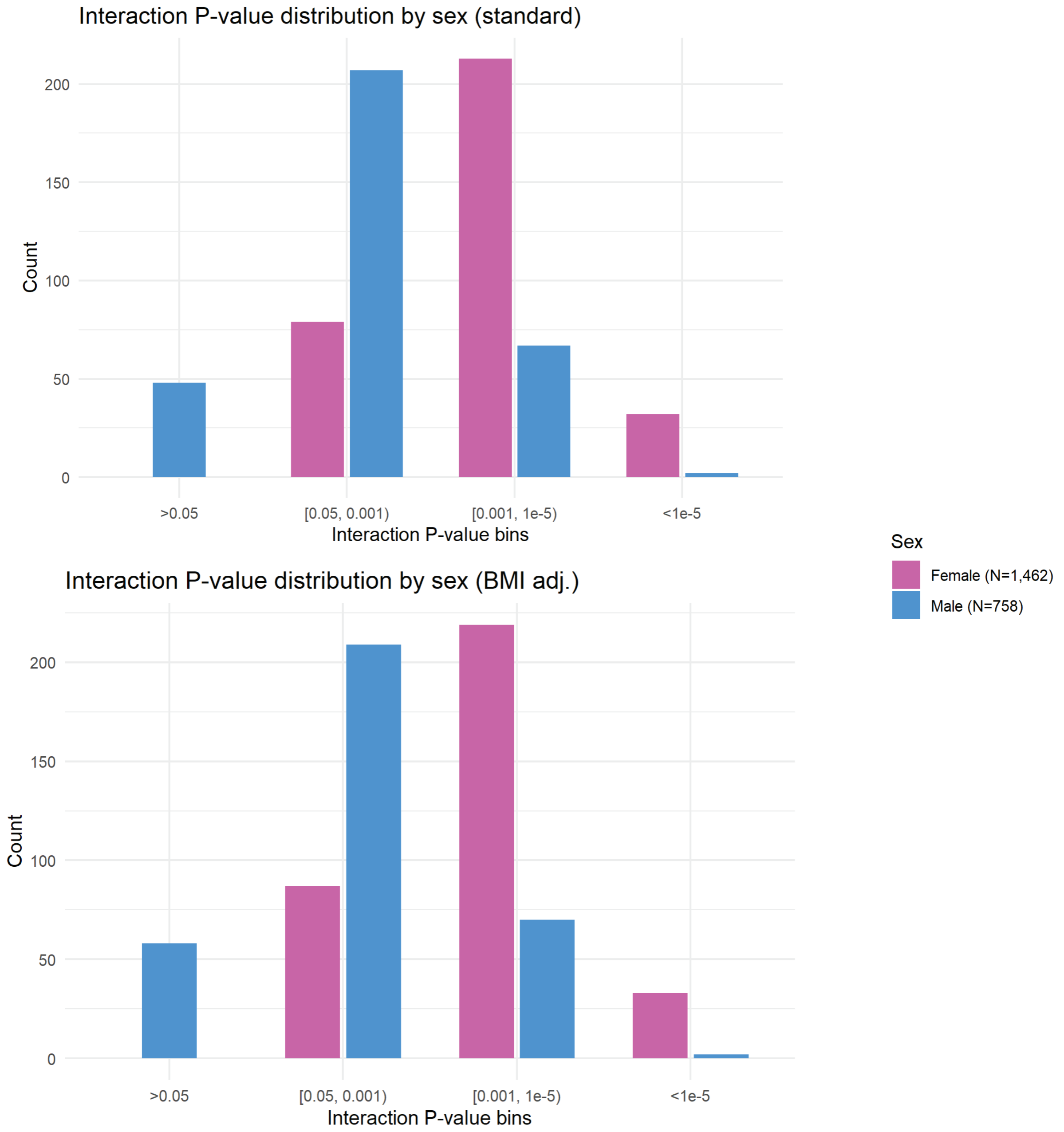

S8 Fig. Summary of interaction *P*-values in sex-stratified analysis. Bars show bin counts of interaction *P*-values from males and females for all interactions reaching suggestive significance in the full cohort genome-wide interaction analysis. Sex-stratified models were also run with and without BMI adjustment, as in the full cohort.
