# Supplementary material for: Gene-vegetarianism interactions in calcium, estimated glomerular filtration rate, and testosterone identified in genome-wide analysis across 30 biomarkers
Source: PLoS Genet. 2024 Jul 11;20(7):e1011288. doi: 10.1371/journal.pgen.1011288 (PMC11239071; doi:10.1371/journal.pgen.1011288)
Supplement: S9 Fig — Violin plots showing expression quantitative trait loci (eQTLs) in four tissues which were significant at the GTEx multiple testing threshold (adipose: subcutaneous, colon: sigmoid, muscle: skeletal, and cells: cultured fibroblasts) plus liver tissue, which nearly reached significance. In all five of these tissues, the heterozygote (CT) shows higher median normalized expression. (PDF) [file pgen.1011288.s019.pdf]

S9

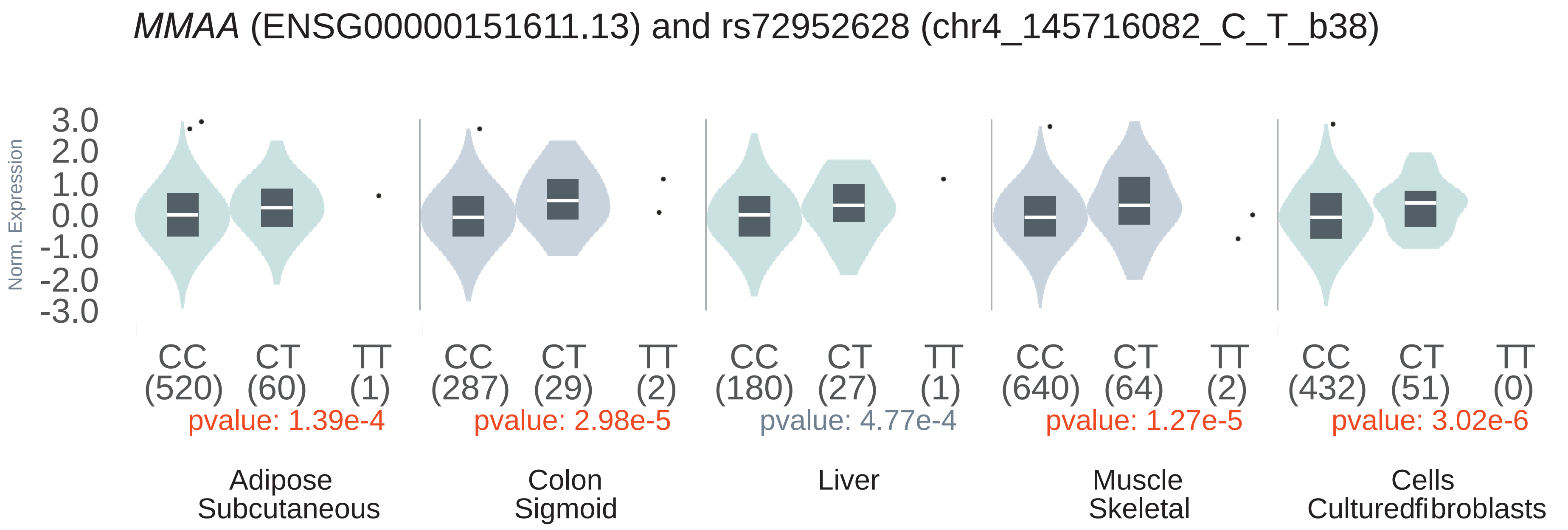

S9 Fig. eQTLs for *MMAA* and rs72952628. Violin plots showing expression quantitative trait loci (eQTLs) in four tissues which were significant at the GTEx multiple testing threshold (adipose: subcutaneous, colon: sigmoid, muscle: skeletal, and cells: cultured fibroblasts) plus liver tissue, which nearly reached significance. In all five of these tissues, the heterozygote (CT) shows higher median normalized expression.
