# Supplementary material for: Gene-vegetarianism interactions in calcium, estimated glomerular filtration rate, and testosterone identified in genome-wide analysis across 30 biomarkers
Source: PLoS Genet. 2024 Jul 11;20(7):e1011288. doi: 10.1371/journal.pgen.1011288 (PMC11239071; doi:10.1371/journal.pgen.1011288)
Supplement: S10 Fig — Candidate genes for significant interactions with vegetarianism in variant-level and gene-level analyses. Transcripts per million (TPM) shown in tissues ranked from low to high for the genes (a) MMAA, (b) RNF168, and (c) DOCK5. (PDF) [file pgen.1011288.s020.pdf]

S10a

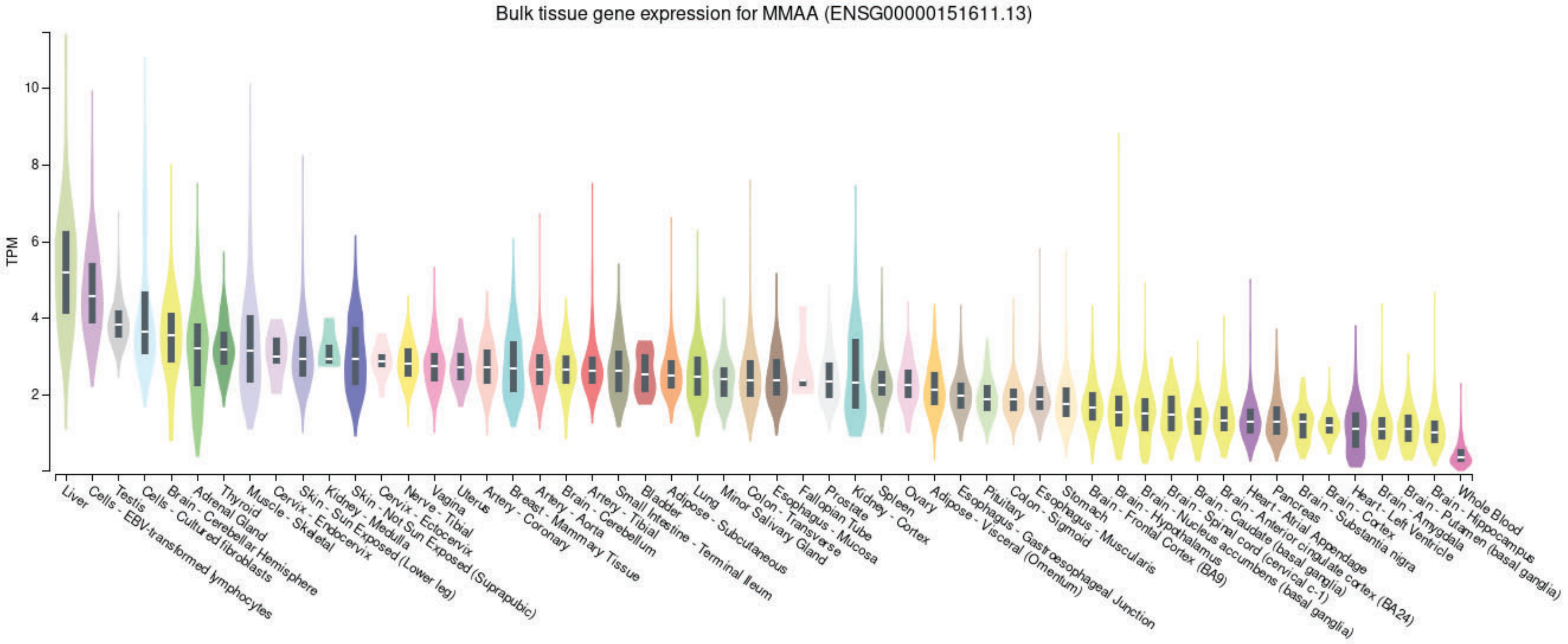
Bulk tissue gene expression for MMAA (ENSG00000151611.13)
TPM
10
8
6
4
2
Liver
Cells - EBV-transformed lymphocytes
Testis
Cells - Cultured fibroblasts
Brain - Cerebellar Hemisphere
Adrenal Gland
Thyroid
Muscle - Skeletal
Cervix - Endocervix
Skin - Sun Exposed (Lower leg)
Kidney - Medulla
Skin - Not Sun Exposed (Suprapubic)
Cervix - Ectocervix
Nerve - Tibial
Vagina
Uterus
Artery - Coronary
Breast - Mammary Tissue
Artery - Aorta
Brain - Cerebellum
Artery - Tibial
Small Intestine - Terminal Ileum
Bladder
Adipose - Subcutaneous
Lung
Minor Salivary Gland
Colon - Transverse
Esophagus - Mucosa
Fallopian Tube
Prostate
Kidney - Cortex
Spleen
Ovary
Adipose - Visceral (Omentum)
Esophagus - Gastroesophageal Junction
Pituitary
Colon - Sigmoid
Esophagus - Muscularis
Stomach
Brain - Frontal Cortex (BA9)
Brain - Hypothalamus
Brain - Nucleus accumbens (basal ganglia)
Brain - Spinal cord (cervical c-1)
Brain - Caudate (basal ganglia)
Brain - Anterior cingulate cortex (BA24)
Heart - Atrial Appendage
Pancreas
Brain - Substantia nigra
Brain - Cortex
Heart - Left Ventricle
Brain - Amygdala
Brain - Putamen (basal ganglia)
Brain - Hippocampus
Whole Blood

b

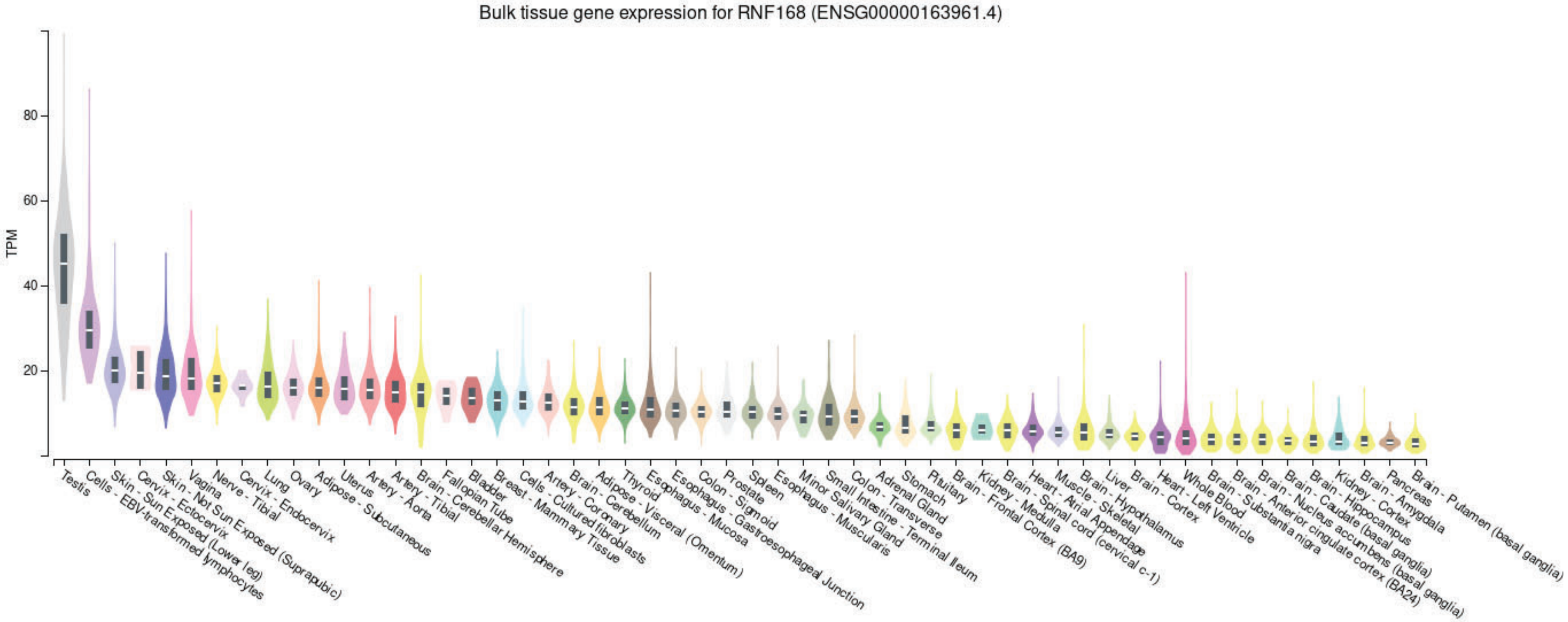
Bulk tissue gene expression for RNF168 (ENSG00000163961.4)
TPM
80
60
40
20
Testis
Cells - EBV-transformed lymphocytes
Skin - Sun Exposed (Lower leg)
Cervix - Ectocervix
Skin - Not Sun Exposed (Suprapubic)
Vagina
Nerve - Tibial
Cervix - Endocervix
Lung
Ovary
Adipose - Subcutaneous
Uterus
Artery - Aorta
Artery - Tibial
Brain - Cerebellar Hemisphere
Fallopian Tube
Bladder
Breast - Mammary Tissue
Cells - Cultured fibroblasts
Artery - Coronary
Brain - Cerebellum
Adipose - Visceral (Omentum)
Thyroid
Esophagus - Mucosa
Colon - Sigmoid
Esophagus - Gastroesophageal Junction
Prostate
Spleen
Esophagus - Muscularis
Minor Salivary Gland
Small Intestine - Terminal Ileum
Colon - Transverse
Adrenal Gland
Stomach
Pituitary
Brain - Frontal Cortex (BA9)
Kidney - Medulla
Brain - Spinal cord (cervical c-1)
Heart - Atrial Appendage
Muscle - Skeletal
Brain - Hypothalamus
Liver
Brain - Cortex
Heart - Left Ventricle
Whole Blood
Brain - Substantia nigra
Brain - Anterior cingulate cortex (BA24)
Brain - Nucleus accumbens (basal ganglia)
Brain - Caudate (basal ganglia)
Brain - Hippocampus
Kidney - Cortex
Brain - Amygdala
Pancreas
Brain - Putamen (basal ganglia)

S10c

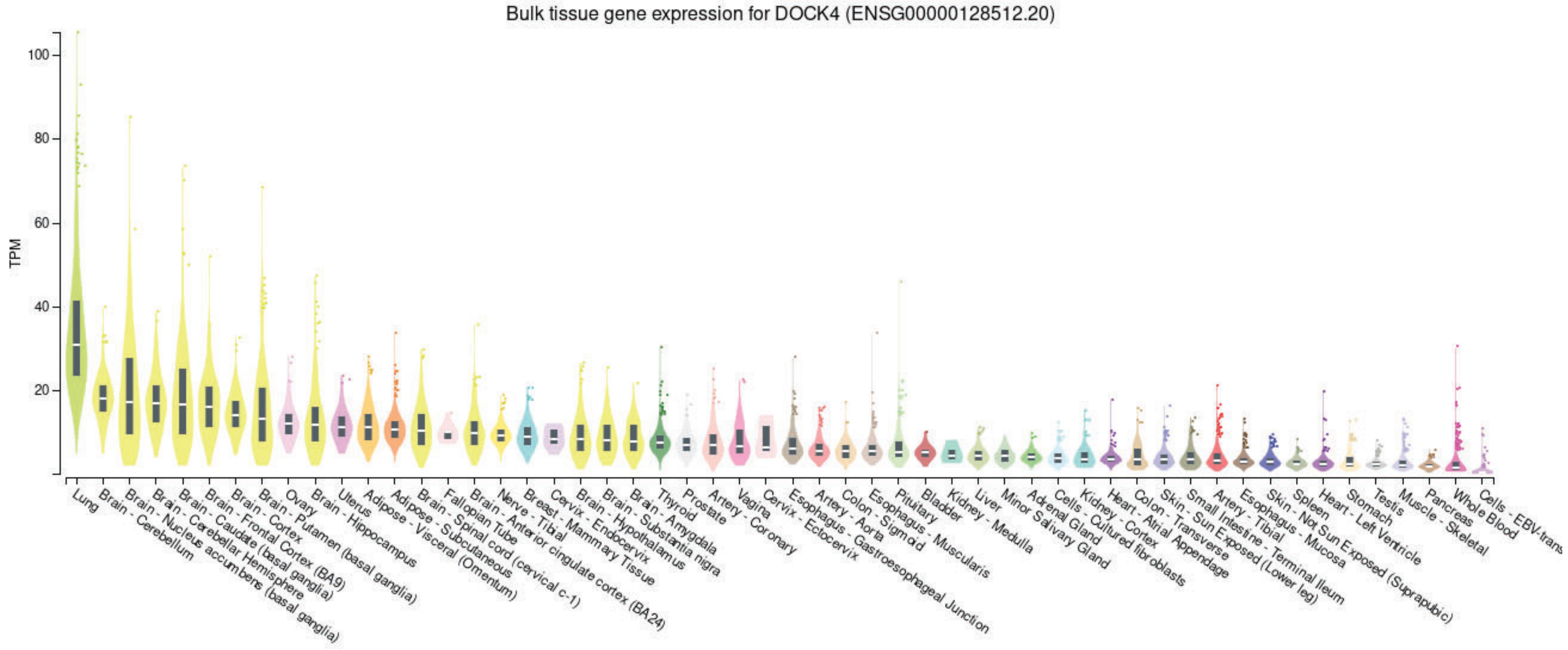

S10 Fig. Bulk tissue gene expression for interaction genes. Candidate genes for significant interactions with vegetarianism in variant-level and gene-level analyses. Transcripts per million (TPM) shown in tissues ranked from low to high for the genes **(a)** *MMAA*, **(b)** *RNF168*, and **(c)** *DOCK5*.
