# Supplementary material for: Gene-vegetarianism interactions in calcium, estimated glomerular filtration rate, and testosterone identified in genome-wide analysis across 30 biomarkers
Source: PLoS Genet. 2024 Jul 11;20(7):e1011288. doi: 10.1371/journal.pgen.1011288 (PMC11239071; doi:10.1371/journal.pgen.1011288)
Supplement: S11 Fig — Manhattan plots and quantile-quantile (QQ) plots showing the gene-level −log10(P) of genome-wide gene-vegetarianism interaction effects in thirty serum biomarker traits. The red line corresponds to the genome-wide significance threshold (P<2.75×10^−6^; red line). In the standard interaction model (a) two traits, estimated glomerular filtration rate (eGFR) and testosterone, had a significant gene. (b) Testosterone had one significant gene in the BMI-adjusted model. (PDF) [file pgen.1011288.s021.pdf]

S11a

Alanine aminotransferase

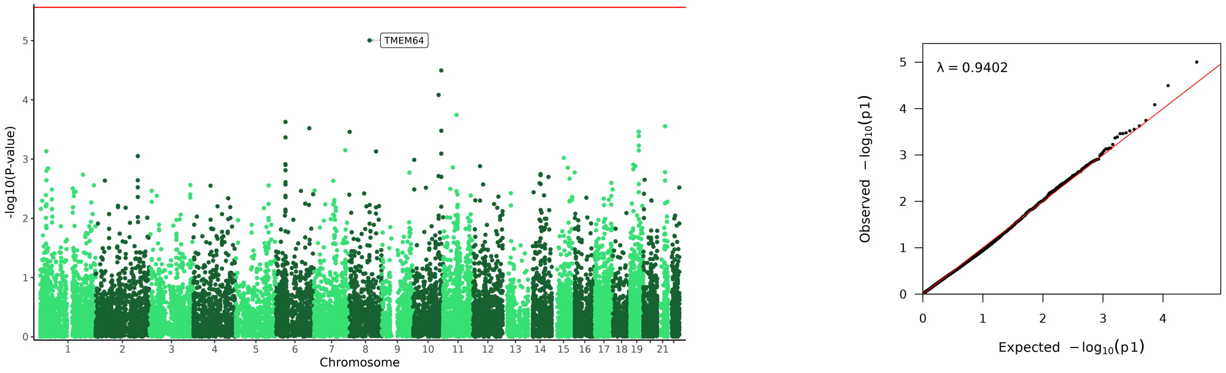

Albumin

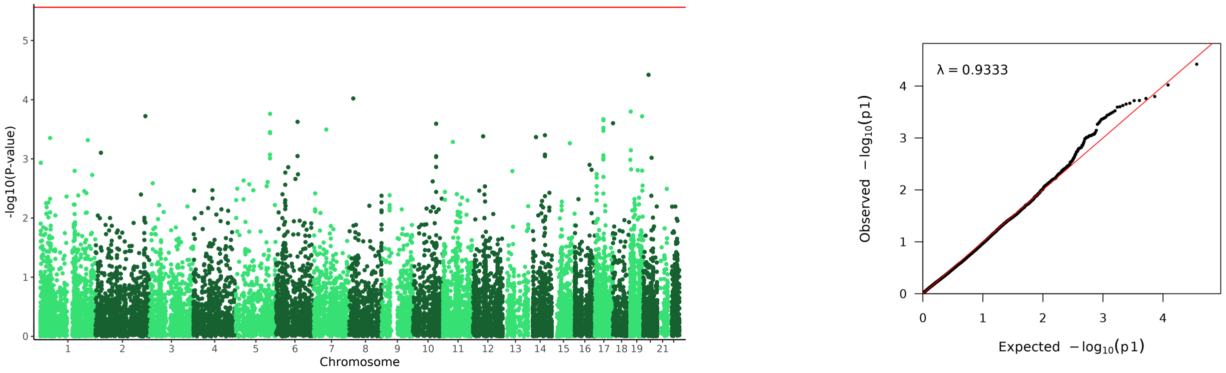

Alkaline phosphatase

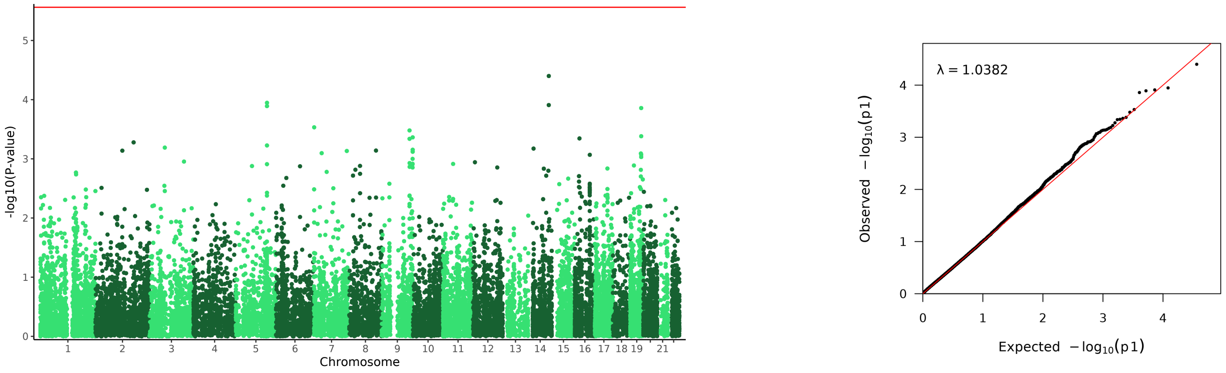

Apolipoprotein A

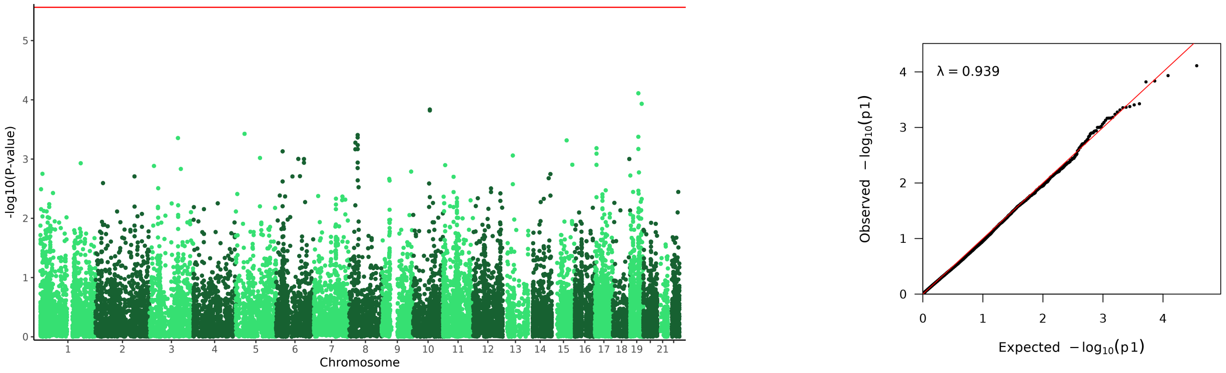

S11a

Apolipoprotein B

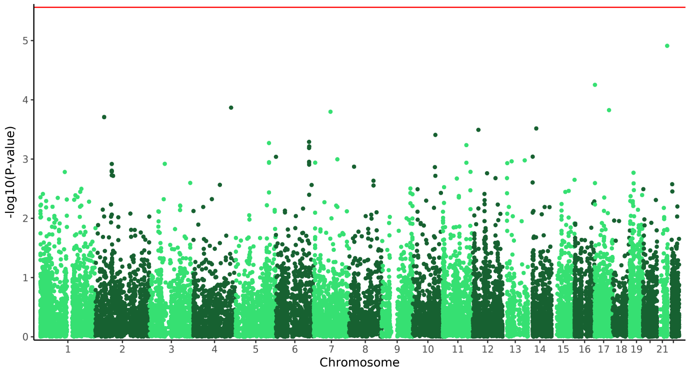

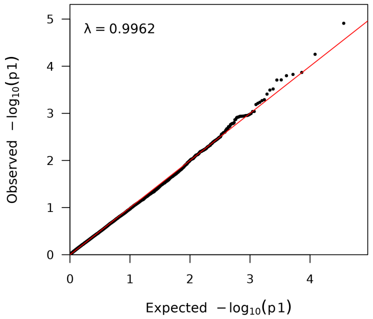

Aspartate aminotransferase

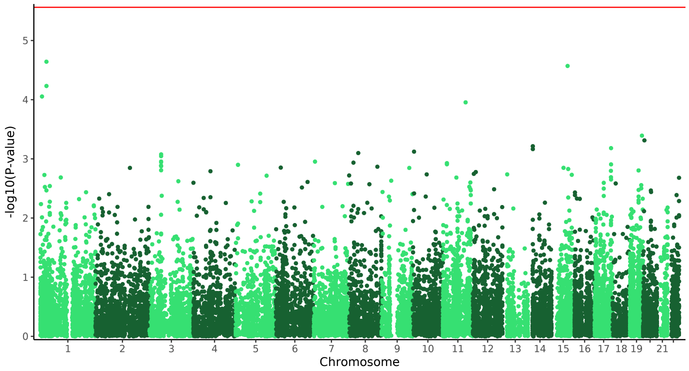

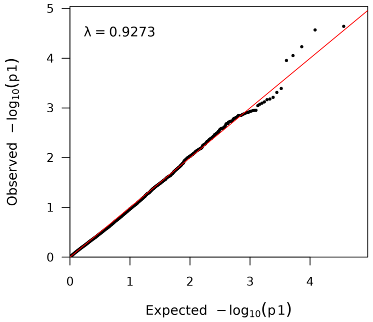

Bioavailable testosterone

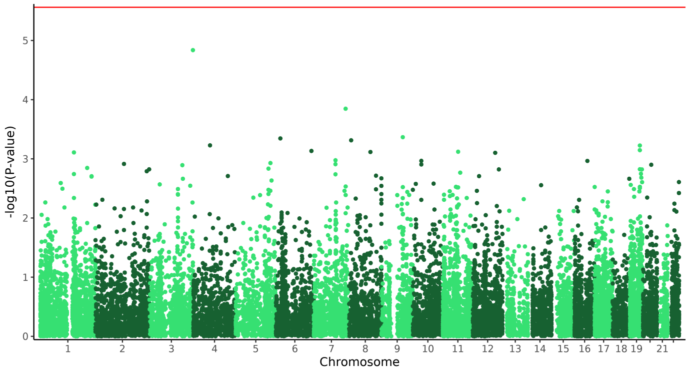

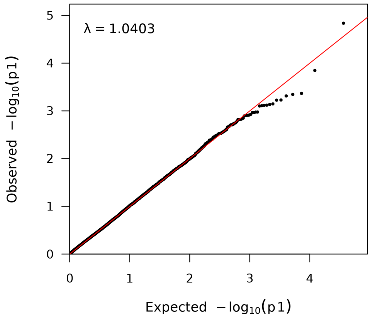

C-reactive protein

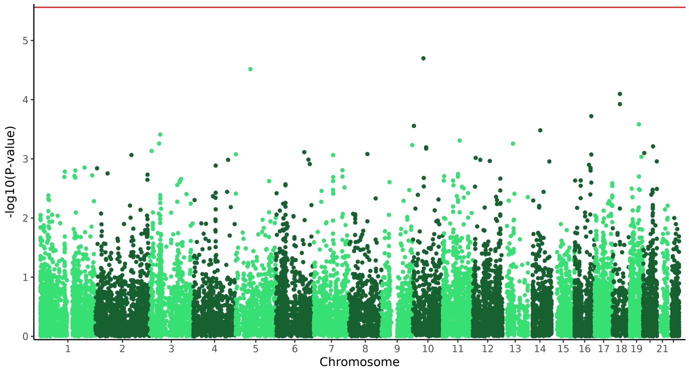

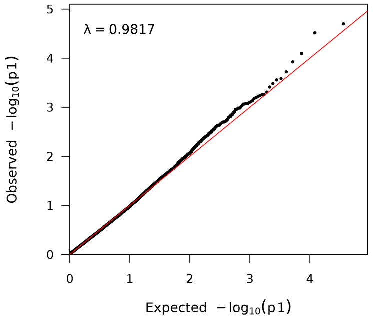

S11a

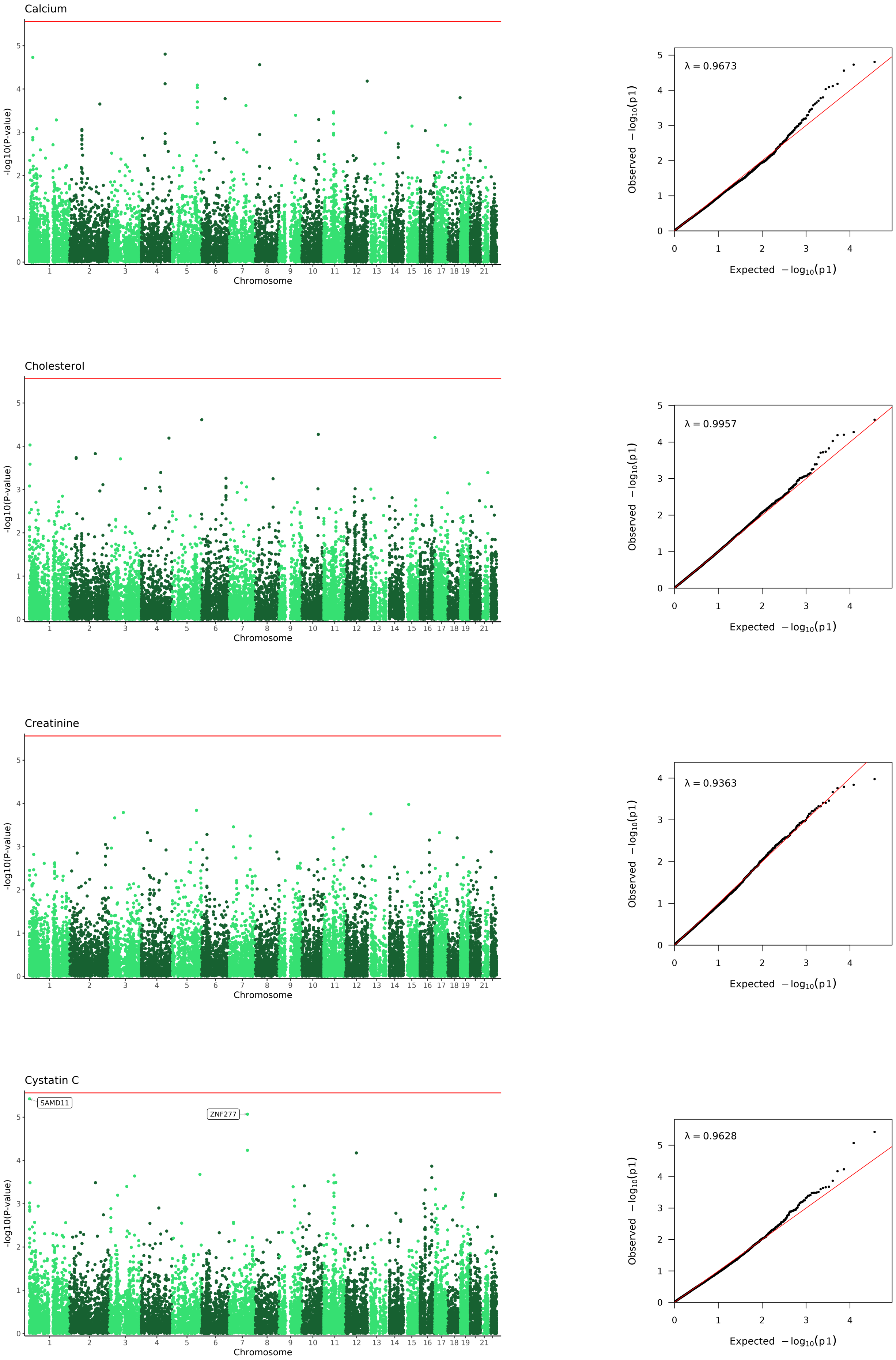
Calcium
Cholesterol
Creatinine
Cystatin C
SAMD11
ZNF277
-log10(P-value)
Chromosome
λ = 0.9673
λ = 0.9957
λ = 0.9363
λ = 0.9628
Observed $-\log_{10}(p1)$
Expected $-\log_{10}(p1)$

S11a

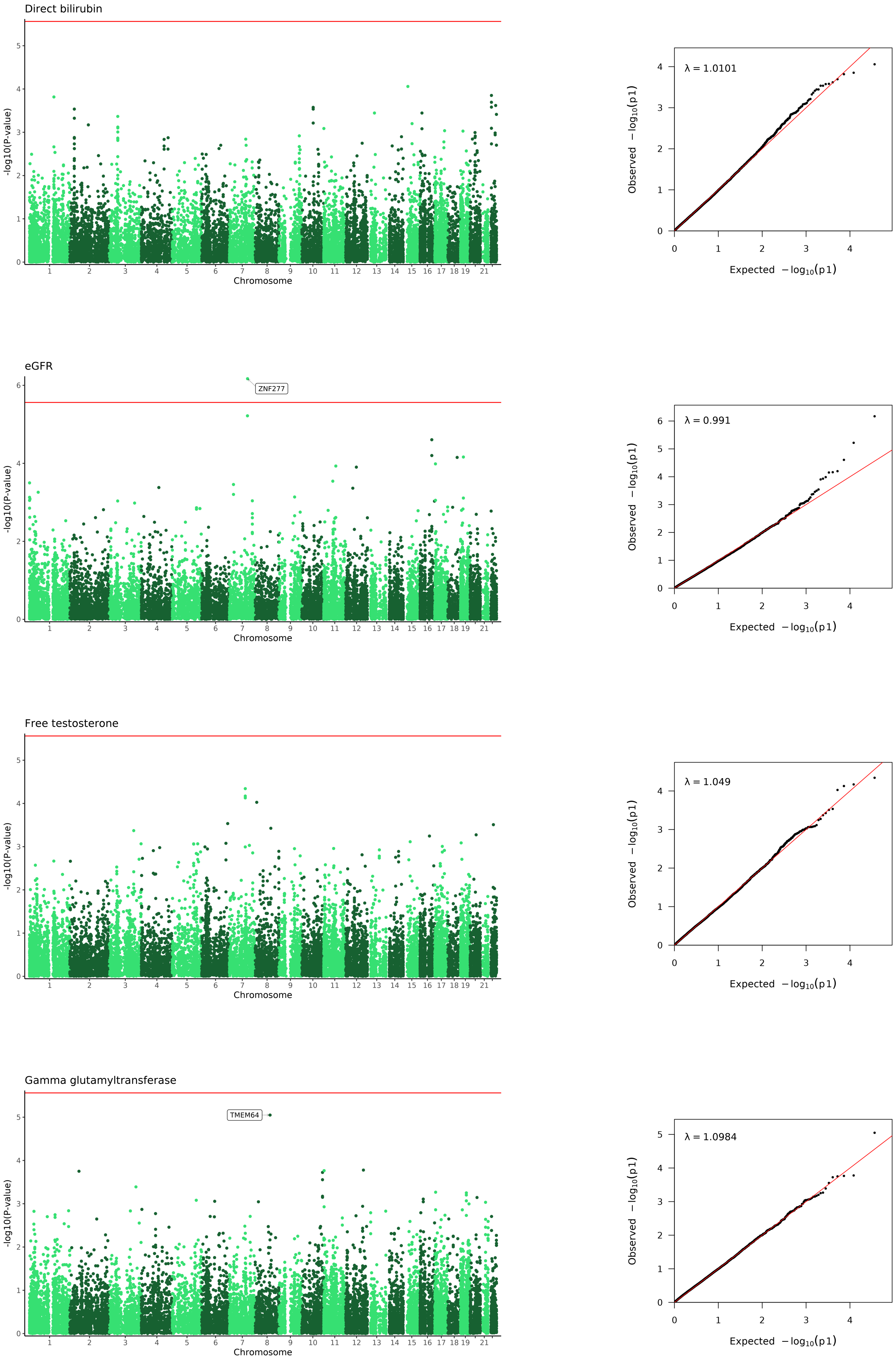
Direct bilirubin
λ = 1.0101
eGFR
ZNF277
λ = 0.991
Free testosterone
λ = 1.049
Gamma glutamyltransferase
TMEM64
λ = 1.0984
-log10(P-value)
Chromosome
Observed $-\log_{10}(p1)$
Expected $-\log_{10}(p1)$

S11a

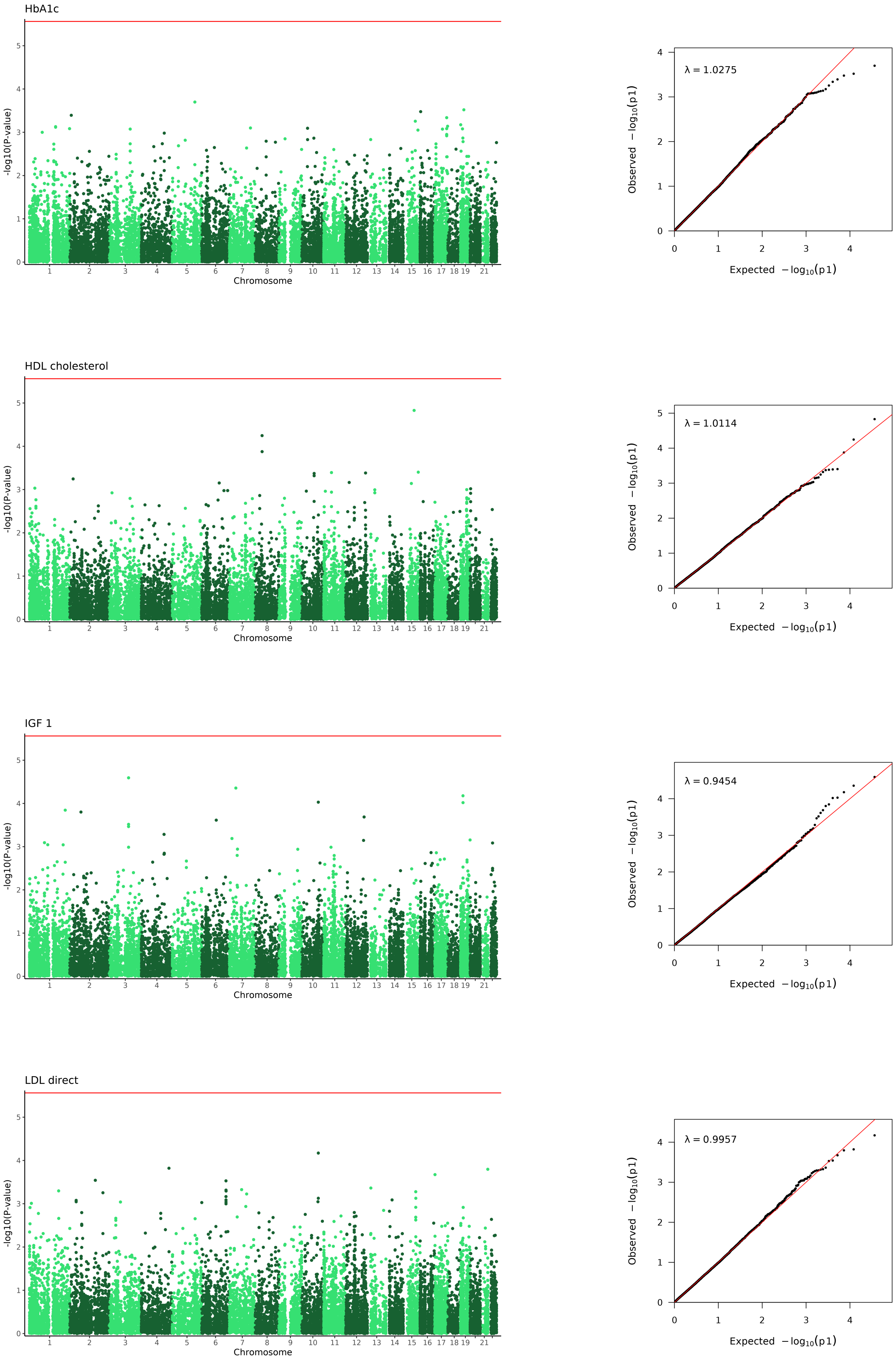
HbA1c
λ = 1.0275
HDL cholesterol
λ = 1.0114
IGF 1
λ = 0.9454
LDL direct
λ = 0.9957
-log10(P-value)
Chromosome
Observed $-\log_{10}(p1)$
Expected $-\log_{10}(p1)$

S11a

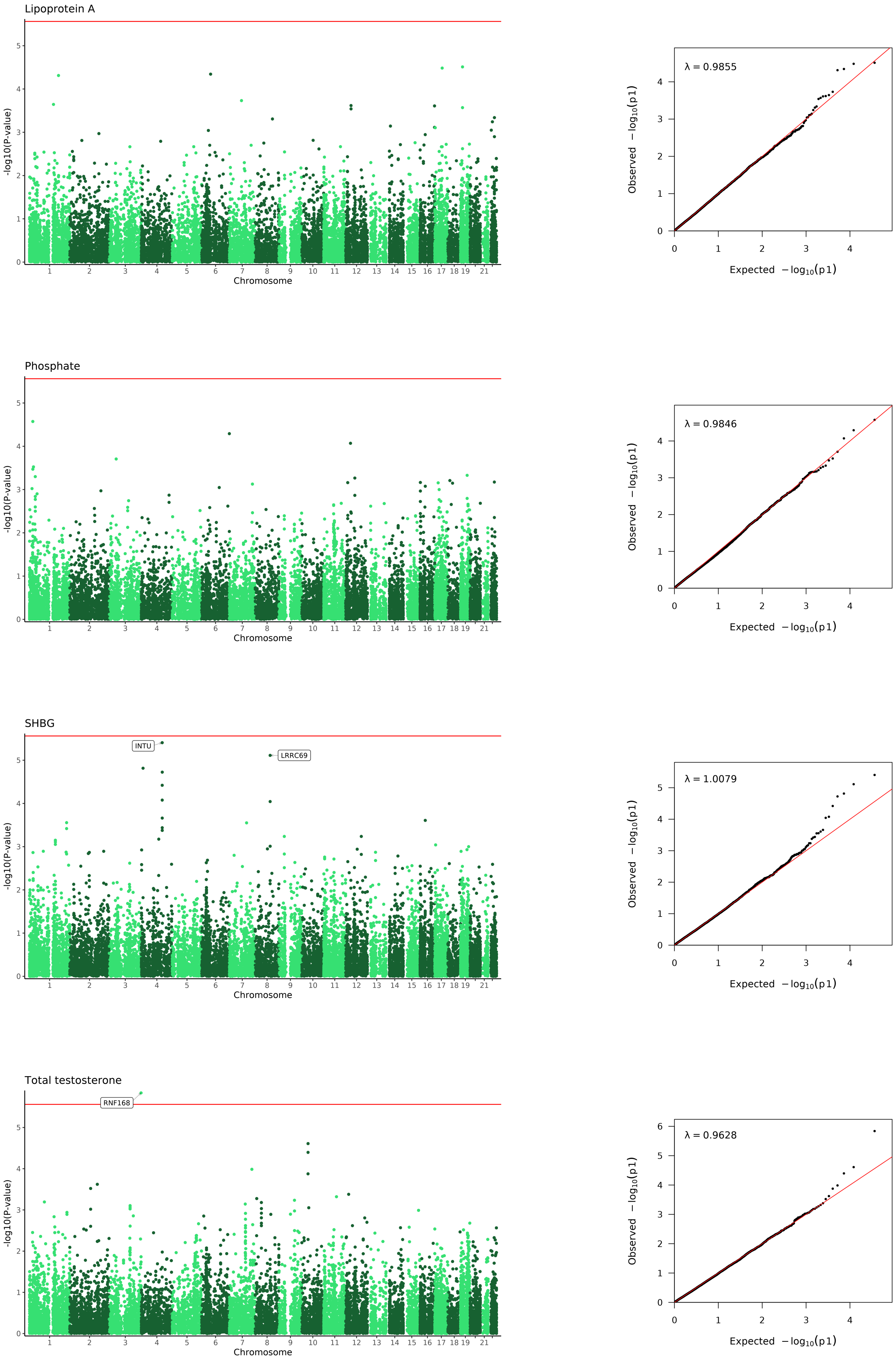
Lipoprotein A
λ = 0.9855
Phosphate
λ = 0.9846
SHBG
INTU
LRRC69
λ = 1.0079
Total testosterone
RNF168
λ = 0.9628
-log10(P-value)
Chromosome
Observed $-\log_{10}(p1)$
Expected $-\log_{10}(p1)$

S11a

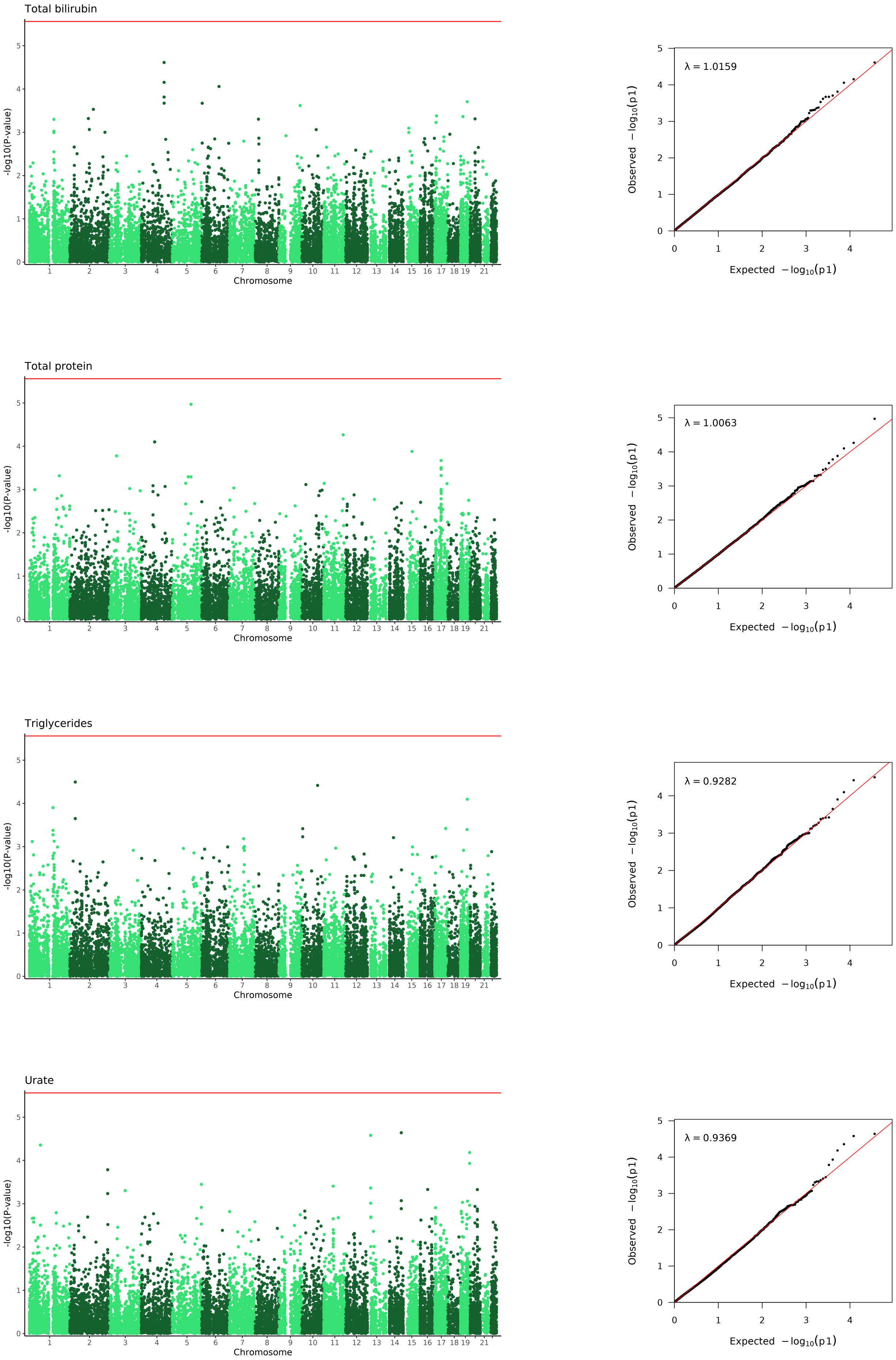
Total bilirubin
-log10(P-value)
Chromosome
λ = 1.0159
Observed $-\log_{10}(p1)$
Expected $-\log_{10}(p1)$
Total protein
-log10(P-value)
Chromosome
λ = 1.0063
Observed $-\log_{10}(p1)$
Expected $-\log_{10}(p1)$
Triglycerides
-log10(P-value)
Chromosome
λ = 0.9282
Observed $-\log_{10}(p1)$
Expected $-\log_{10}(p1)$
Urate
-log10(P-value)
Chromosome
λ = 0.9369
Observed $-\log_{10}(p1)$
Expected $-\log_{10}(p1)$

S11a

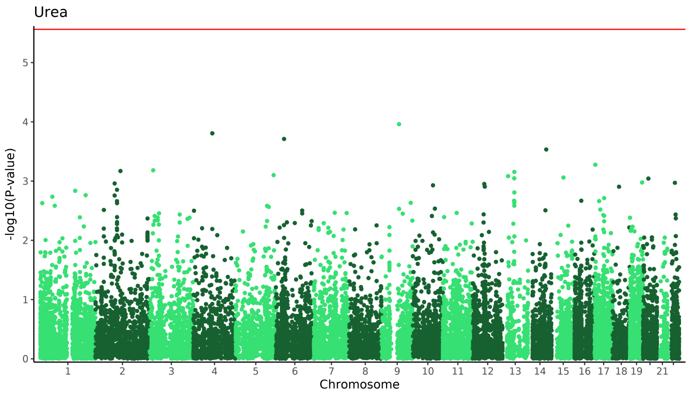
Urea
-log10(P-value)
Chromosome
1
2
3
4
5
6
7
8
9
10
11
12
13
14
15
16
17
18
19
21

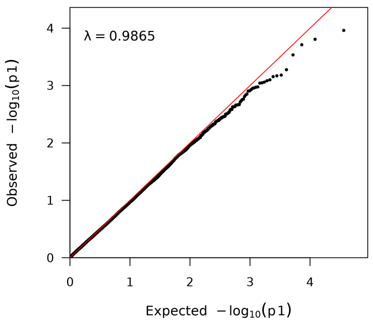
λ = 0.9865
Observed −log10(p1)
Expected −log10(p1)

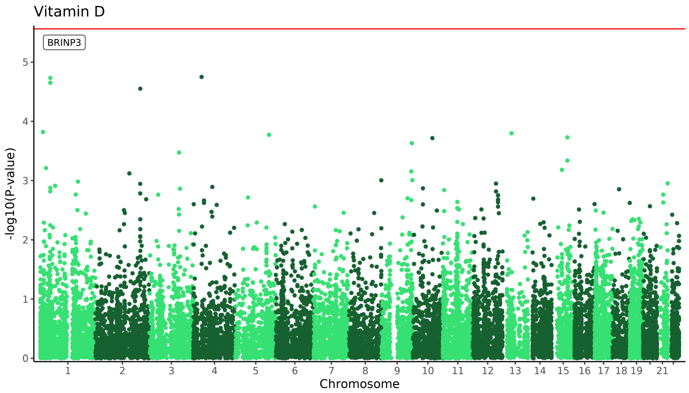
Vitamin D
BRINP3
-log10(P-value)
Chromosome

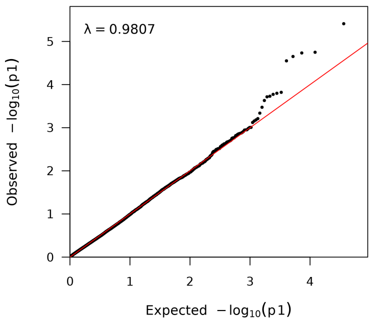
λ = 0.9807
Observed −log10(p1)
Expected −log10(p1)

S11b

Alanine aminotransferase (adj-BMI)

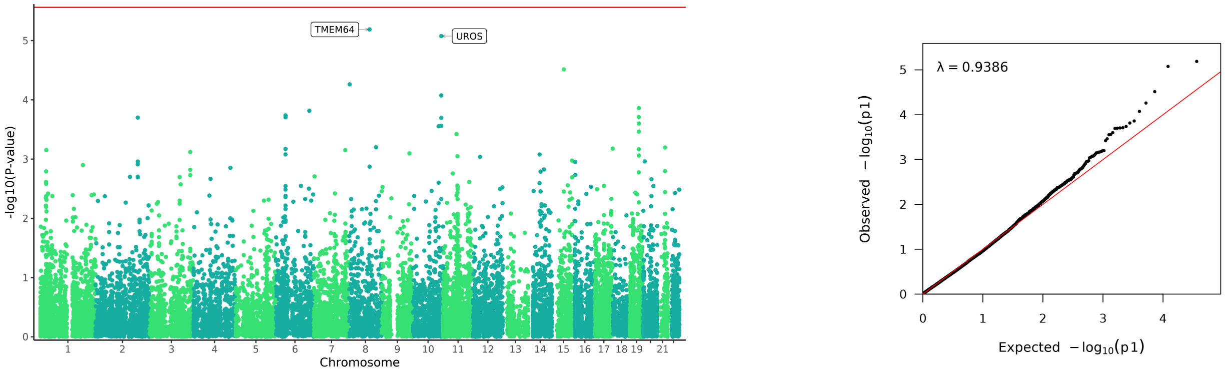

Albumin (adj-BMI)

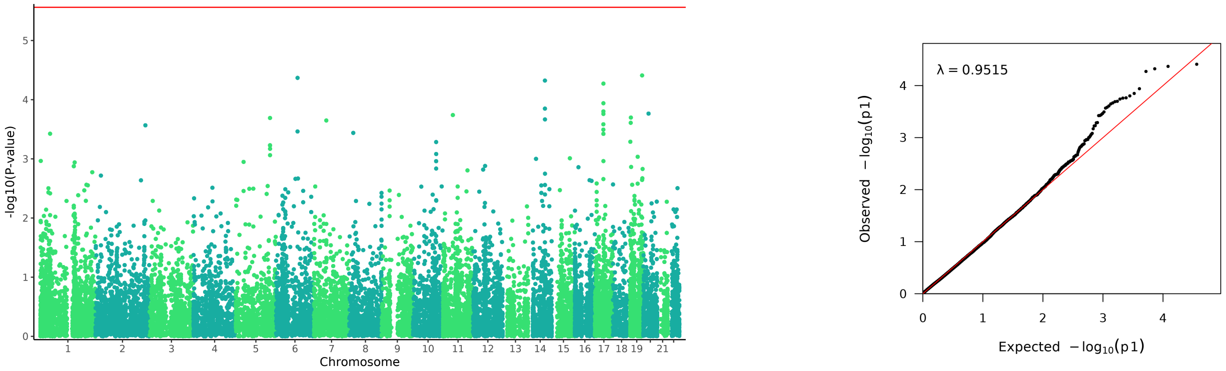

Alkaline phosphatase (adj-BMI)

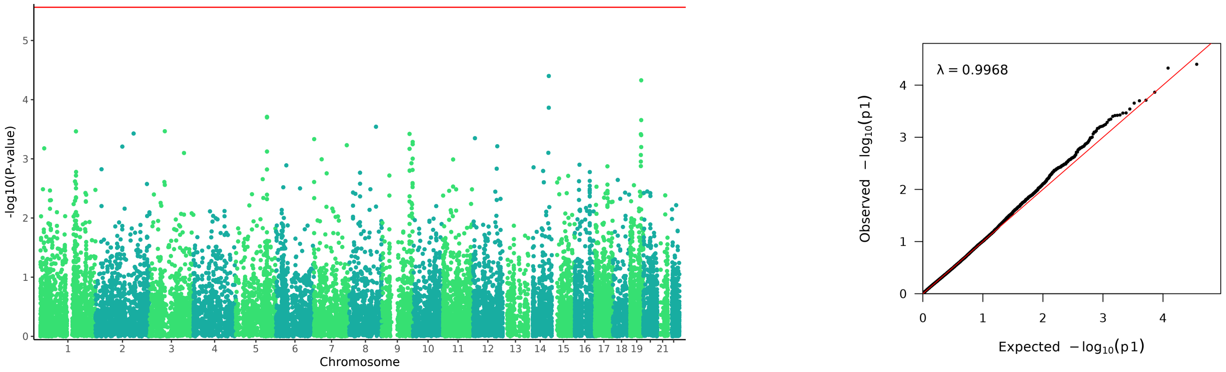

Apolipoprotein A (adj-BMI)

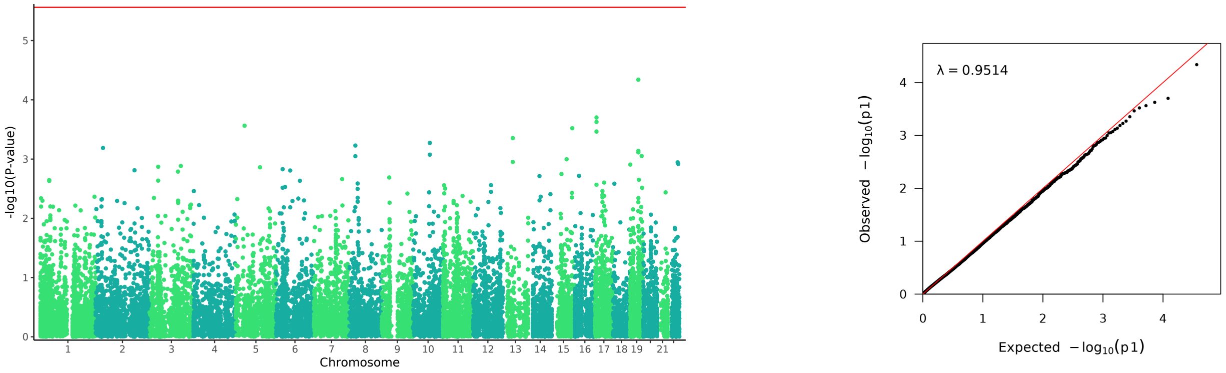

S11b

Apolipoprotein B (adj-BMI)

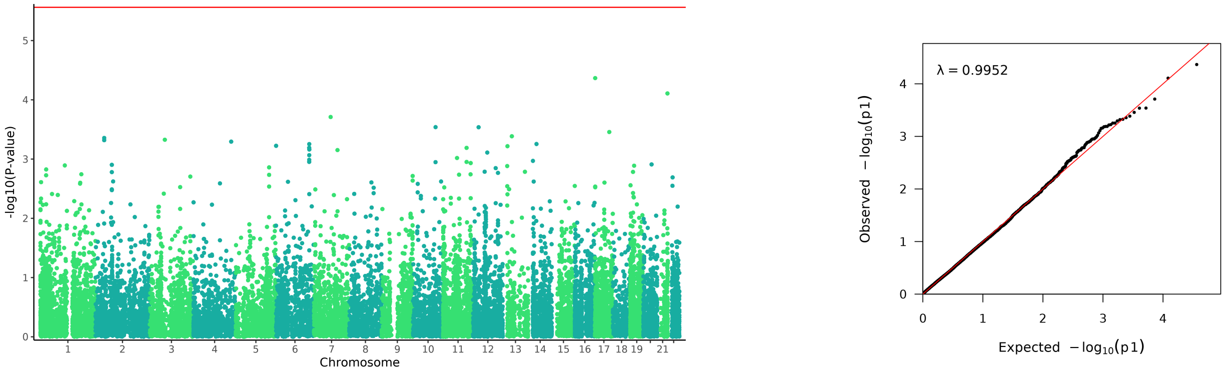

Aspartate aminotransferase (adj-BMI)

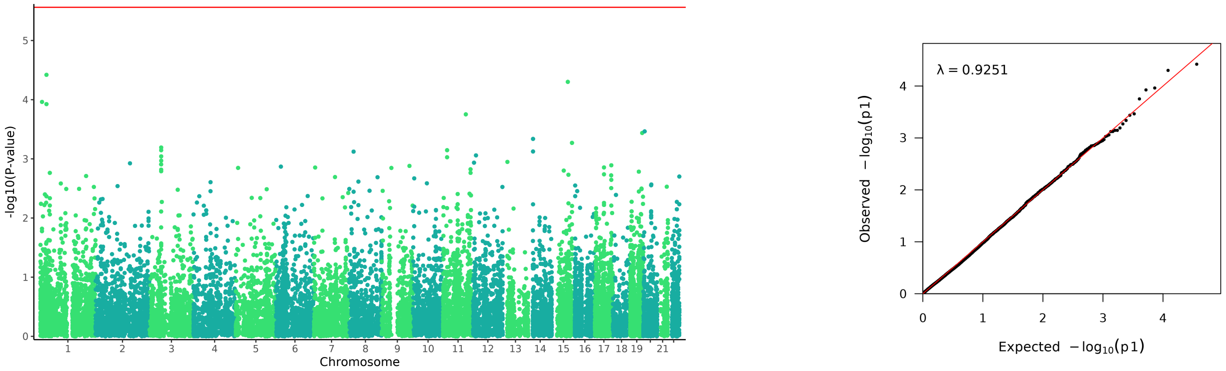

Bioavailable testosterone (adj-BMI)

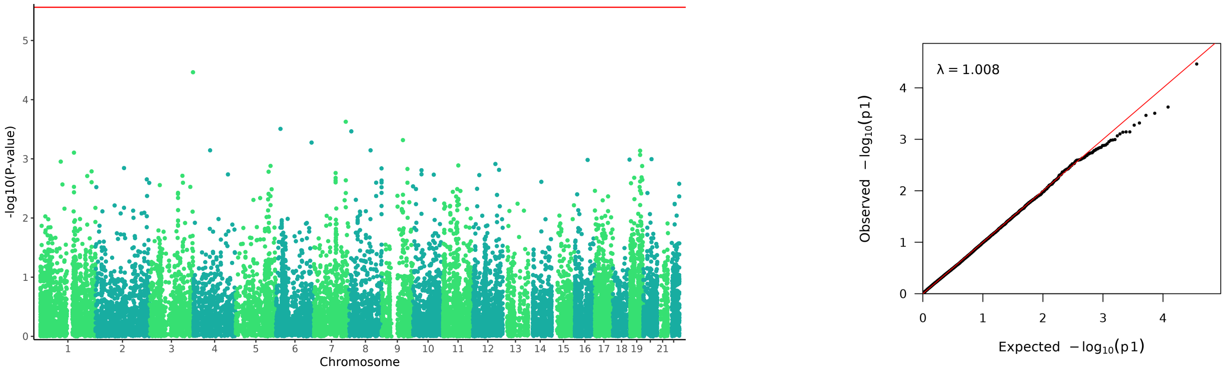

C-reactive protein (adj-BMI)

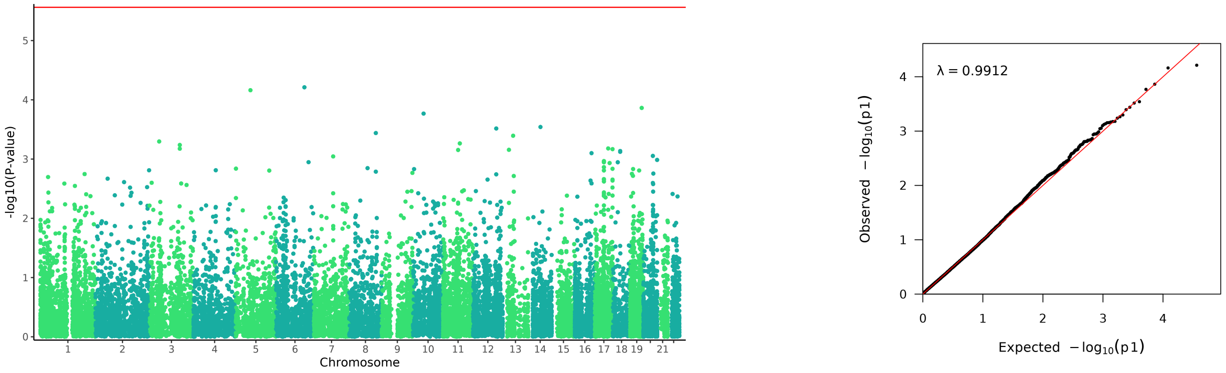

S11b

Calcium (adj-BMI)

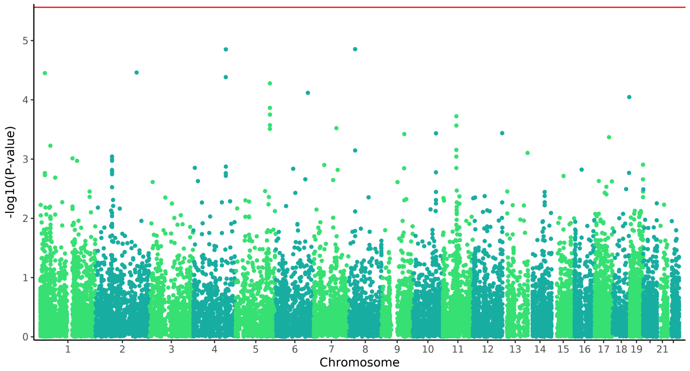

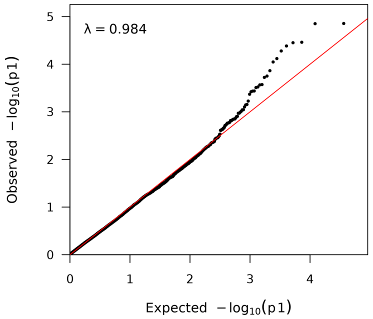

Cholesterol (adj-BMI)

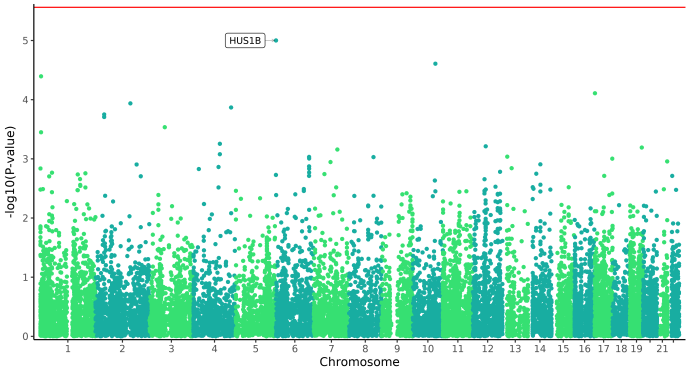

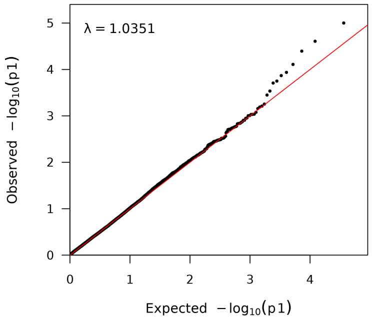

Creatinine (adj-BMI)

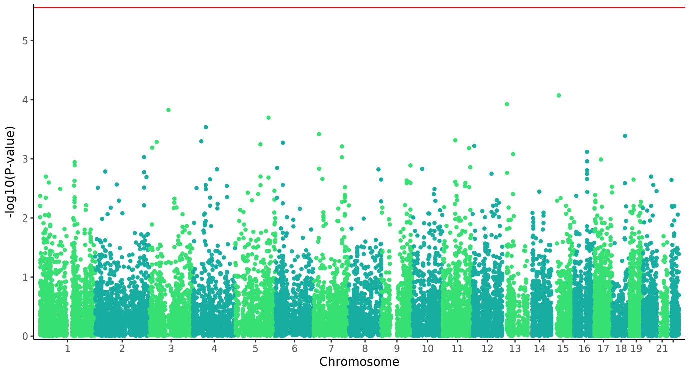

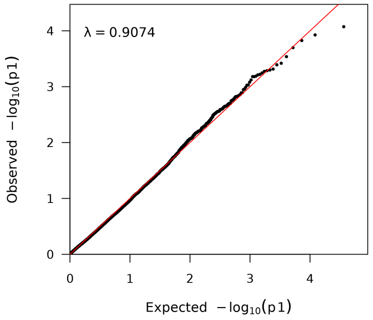

Cystatin C (adj-BMI)

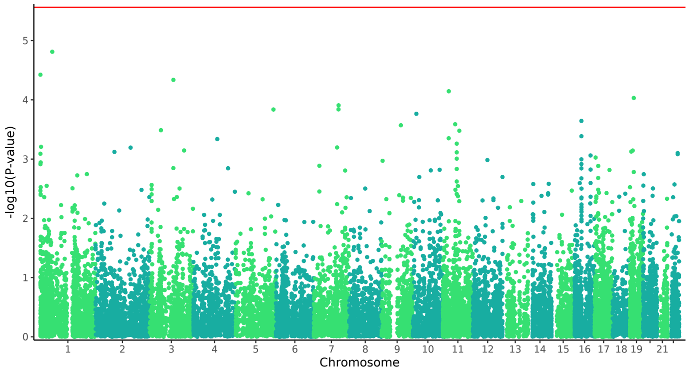

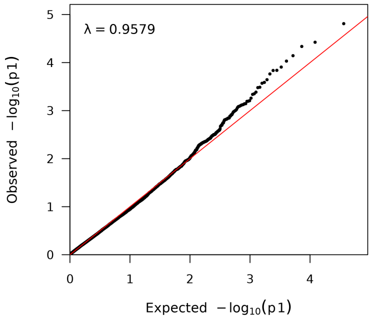

S11b

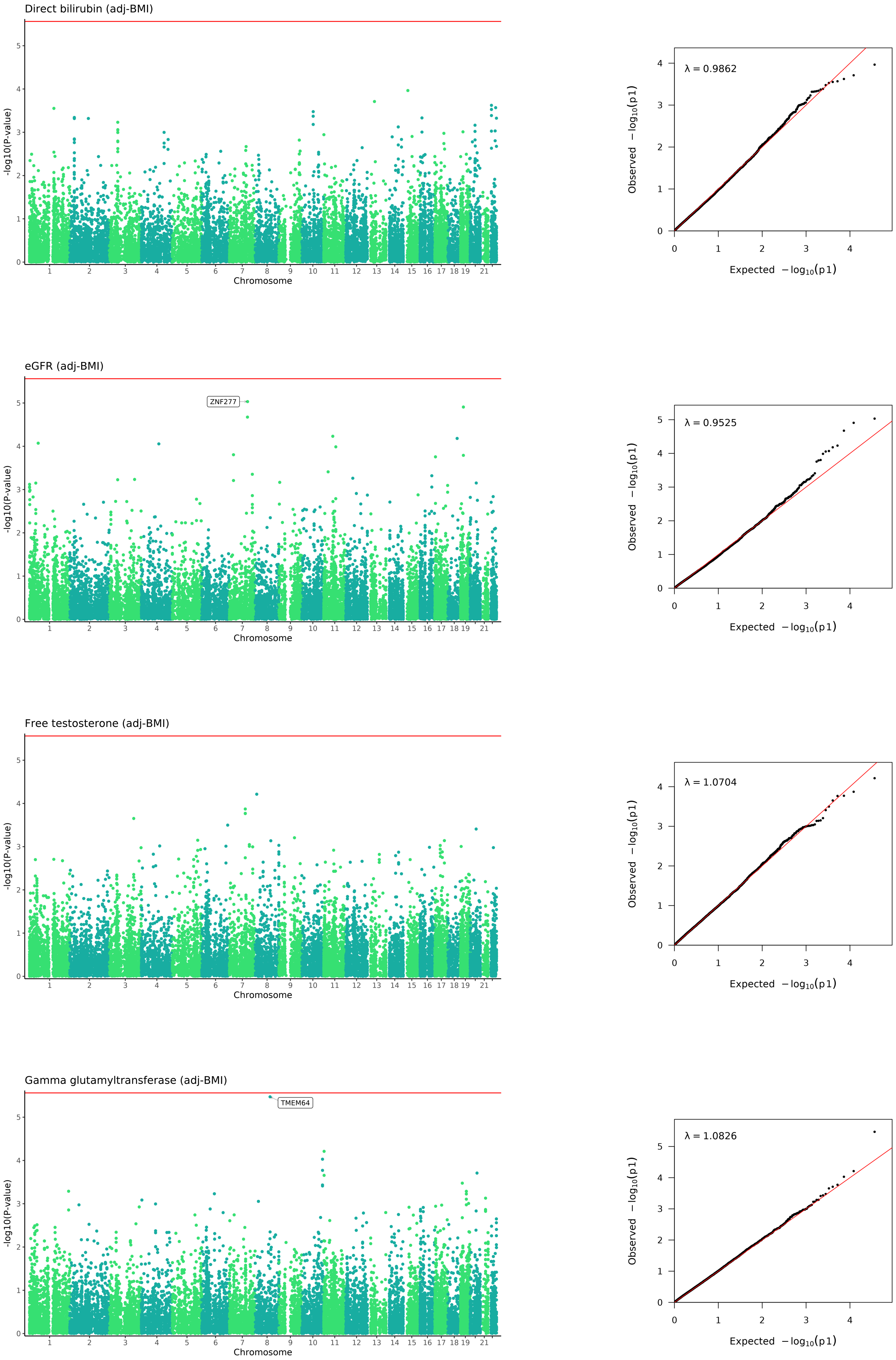
Direct bilirubin (adj-BMI)
λ = 0.9862
eGFR (adj-BMI)
ZNF277
λ = 0.9525
Free testosterone (adj-BMI)
λ = 1.0704
Gamma glutamyltransferase (adj-BMI)
TMEM64
λ = 1.0826
-log10(P-value)
Chromosome
Observed −log10(p1)
Expected −log10(p1)

S11b

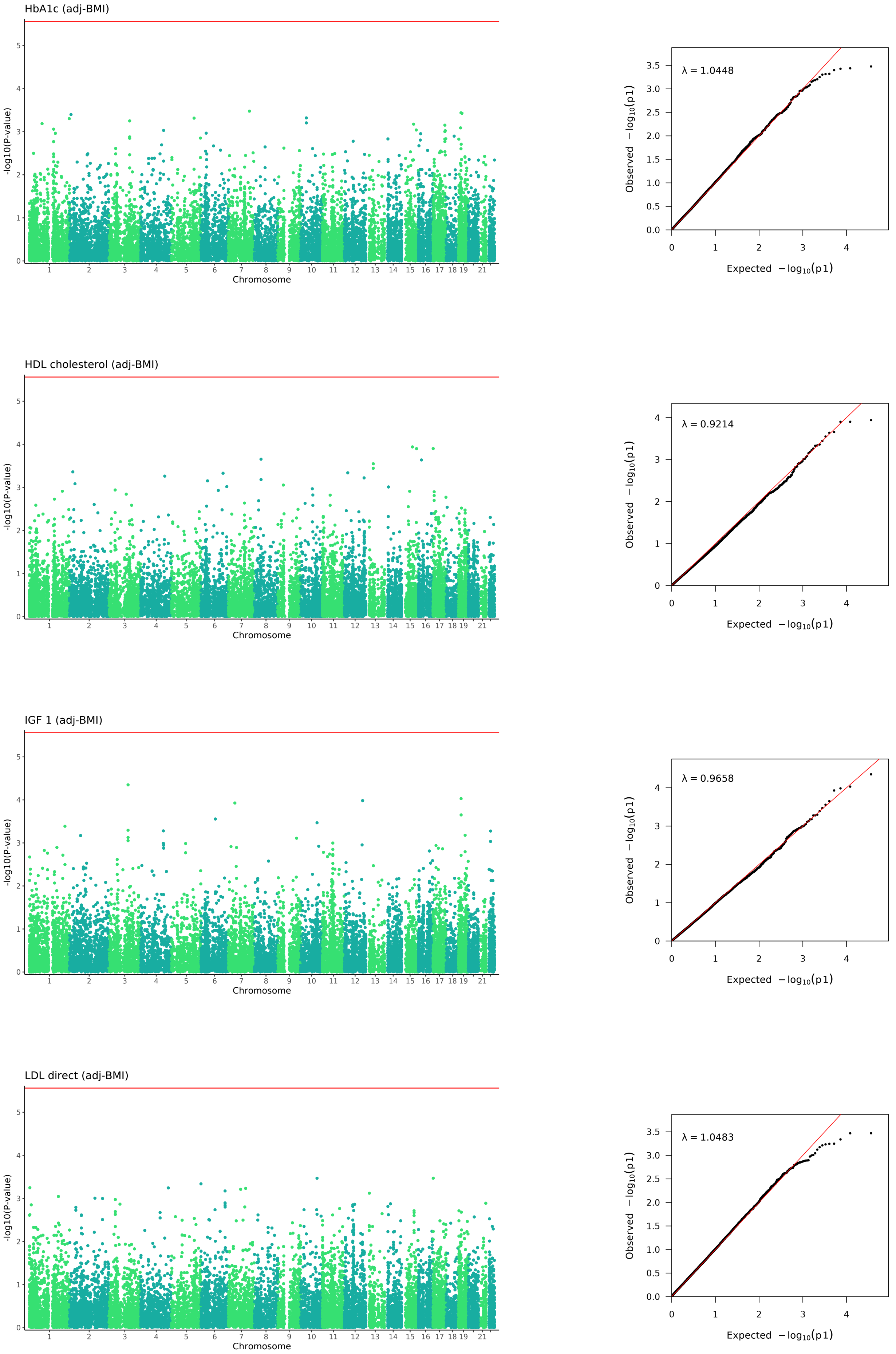

HbA1c (adj-BMI)
λ = 1.0448
HDL cholesterol (adj-BMI)
λ = 0.9214
IGF 1 (adj-BMI)
λ = 0.9658
LDL direct (adj-BMI)
λ = 1.0483
-log10(P-value)
Chromosome
Observed $-\log_{10}(p1)$
Expected $-\log_{10}(p1)$

S11b

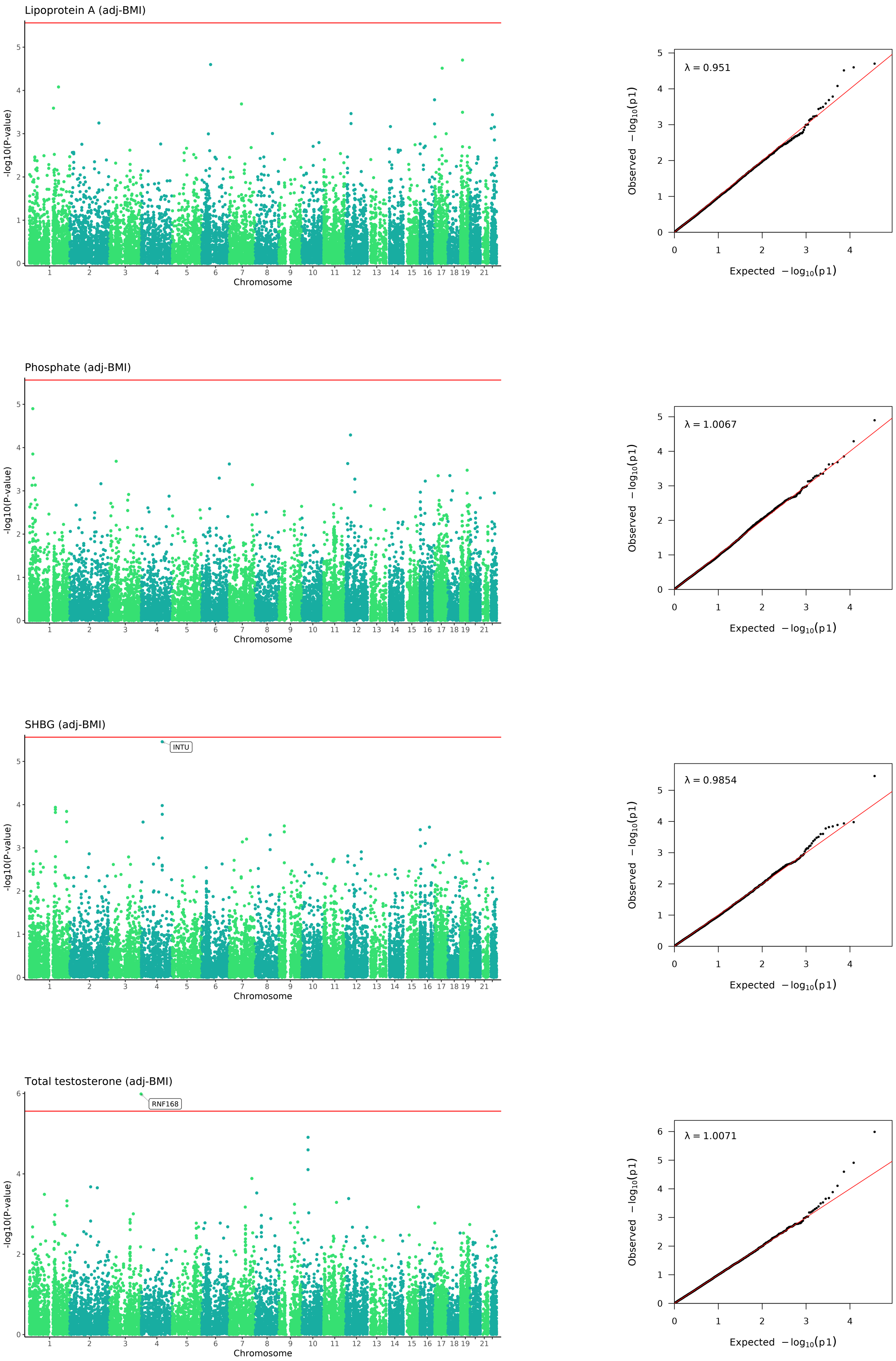

Lipoprotein A (adj-BMI)
-log10(P-value)
Chromosome
λ = 0.951
Observed $-\log_{10}(p1)$
Expected $-\log_{10}(p1)$
Phosphate (adj-BMI)
-log10(P-value)
Chromosome
λ = 1.0067
Observed $-\log_{10}(p1)$
Expected $-\log_{10}(p1)$
SHBG (adj-BMI)
INTU
-log10(P-value)
Chromosome
λ = 0.9854
Observed $-\log_{10}(p1)$
Expected $-\log_{10}(p1)$
Total testosterone (adj-BMI)
RNF168
-log10(P-value)
Chromosome
λ = 1.0071
Observed $-\log_{10}(p1)$
Expected $-\log_{10}(p1)$

S11b

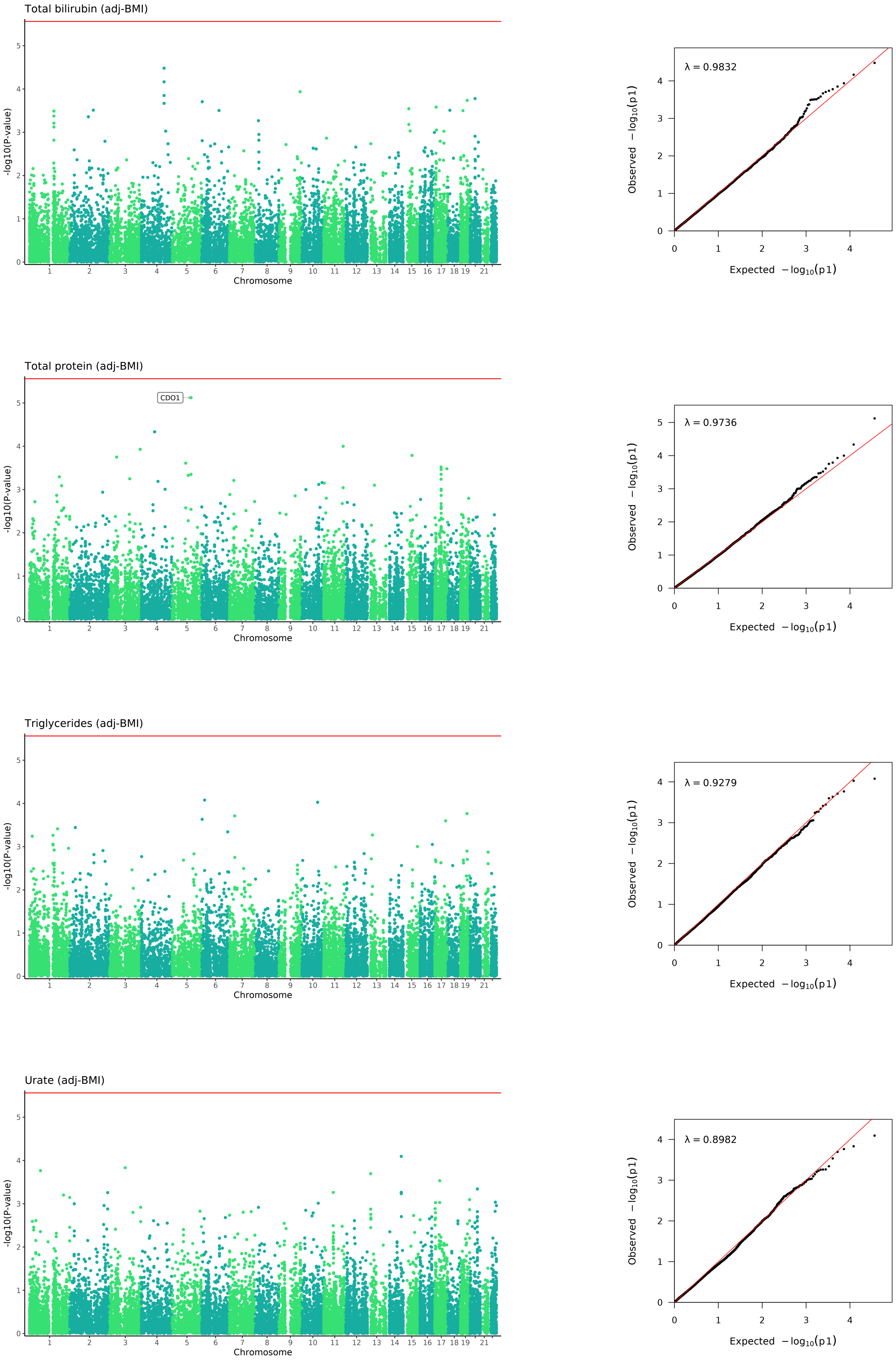
Total bilirubin (adj-BMI)
λ = 0.9832
Total protein (adj-BMI)
CDO1
λ = 0.9736
Triglycerides (adj-BMI)
λ = 0.9279
Urate (adj-BMI)
λ = 0.8982
-log10(P-value)
Chromosome
Observed −log10(p1)
Expected −log10(p1)

S11b

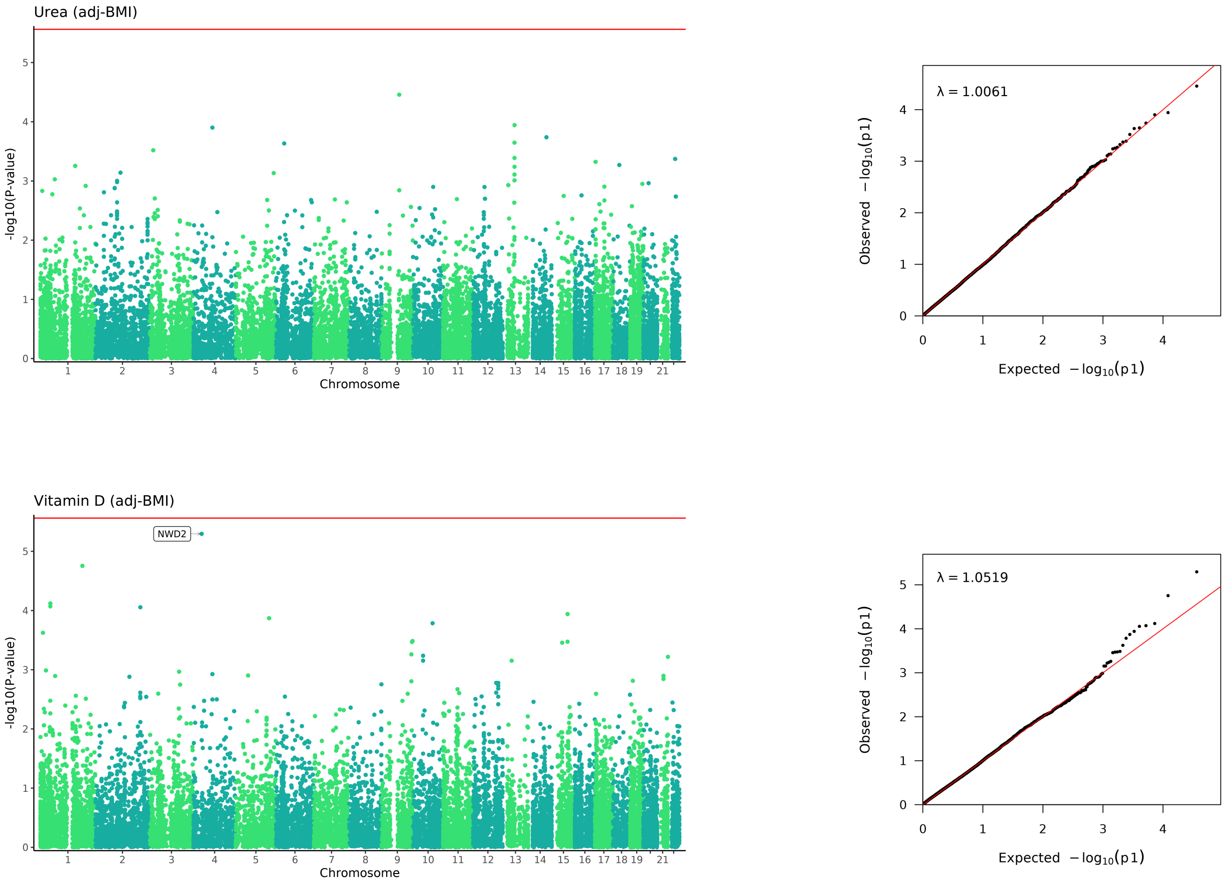

S11 Fig. Gene-level gene-vegetarianism interaction Manhattan plots. Manhattan plots and QQ plots showing the gene-level −log10(*P*) of genome-wide gene-vegetarianism interaction effects in thirty serum biomarker traits. The red line corresponds to the genome-wide significance threshold($P<2.75\times10^{-6}$; red line). In the standard interaction model **(a)** two traits, estimated glomerular filtration rate (eGFR) and testosterone, had a significant gene. **(b)** Testosterone had one significant gene in the BMI-adjusted model.
