# Supplementary material for: Gene-vegetarianism interactions in calcium, estimated glomerular filtration rate, and testosterone identified in genome-wide analysis across 30 biomarkers
Source: PLoS Genet. 2024 Jul 11;20(7):e1011288. doi: 10.1371/journal.pgen.1011288 (PMC11239071; doi:10.1371/journal.pgen.1011288)
Supplement: S12 Fig — Plots show effect of vegetarianism on testosterone in males (left) and females (right), for each genotype at the top associated variant in RNF168 in the testosterone gene-vegetarianism interaction analysis. (PDF) [file pgen.1011288.s022.pdf]

S12

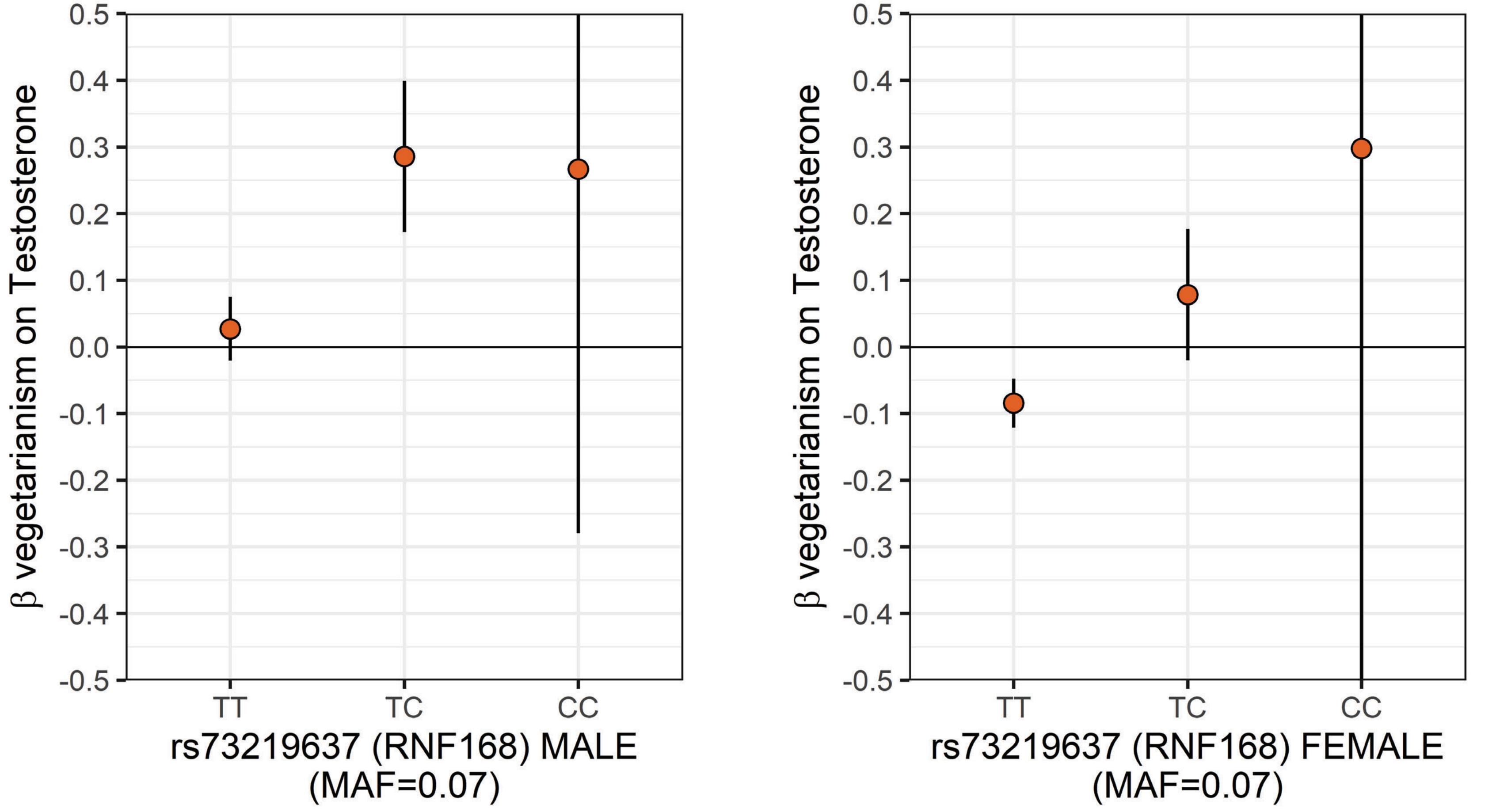

S12 Fig. Genotype-stratified sex-stratified regression models for rs73219637. Plots show effect of vegetarianism on testosterone in males (left) and females (right), for each genotype at the top associated variant in *RNF168* in the testosterone gene-vegetarianism interaction analysis.
