# Supplementary material for: Gene-vegetarianism interactions in calcium, estimated glomerular filtration rate, and testosterone identified in genome-wide analysis across 30 biomarkers
Source: PLoS Genet. 2024 Jul 11;20(7):e1011288. doi: 10.1371/journal.pgen.1011288 (PMC11239071; doi:10.1371/journal.pgen.1011288)
Supplement: S13 Fig — For each of the three lead variants in vegetarianism interactions, the strict vegetarianism selection we developed produced stronger interaction effects and more significant P-values than a single-criterion vegetarianism definition based only on one survey question. (PDF) [file pgen.1011288.s023.pdf]

S13

Veg. criteria	Biomarker	rsID	P-interaction	N vegetarians
Strict	Calcium	rs72952628	4.25e-08	2031
Single	Calcium	rs72952628	7.51e-04	4912
Strict	eGFR	rs17159341	2.58e-07	2215
Single	eGFR	rs17159341	2.57e-04	5373
Strict	Testosterone	rs73219637	7.13e-07	1924
Single	Testosterone	rs73219637	0.006	4626

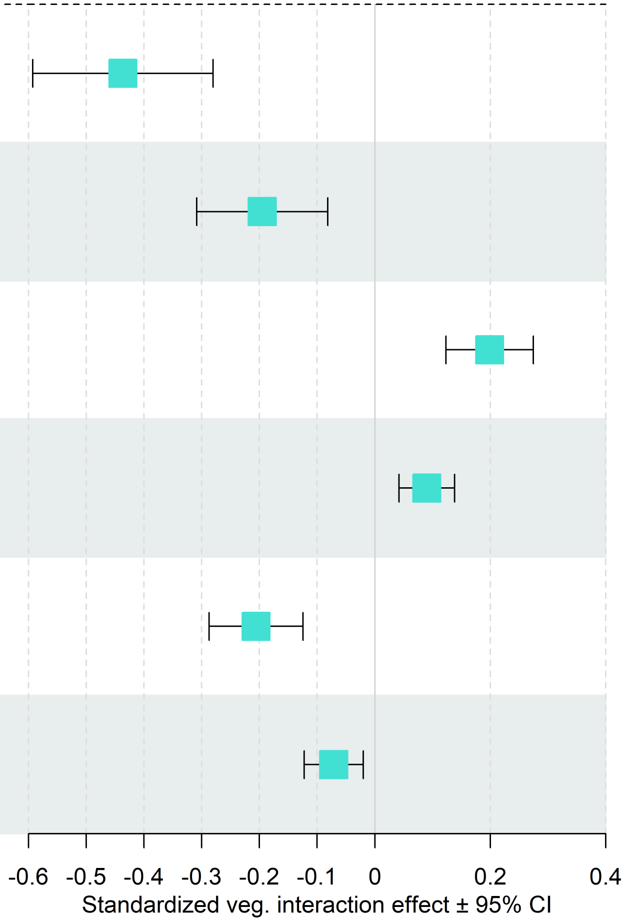

S13 Fig. Forest plot comparing strict versus single-criterion vegetarianism interactions. For each of the three lead variants in vegetarianism interactions, the "strict" vegetarianism selection we developed produced stronger interaction effects and more significant *P*-values than a single-criterion vegetarianism definition based only on one survey question.
