# Supplementary material for: Gene-vegetarianism interactions in calcium, estimated glomerular filtration rate, and testosterone identified in genome-wide analysis across 30 biomarkers
Source: PLoS Genet. 2024 Jul 11;20(7):e1011288. doi: 10.1371/journal.pgen.1011288 (PMC11239071; doi:10.1371/journal.pgen.1011288)
Supplement: S14 Fig — Bar plot shows non-oily-fish- and oily-fish-eating frequency, reported at the initial assessment, for those who reported on that same dietary survey that they had “never eaten meat in [their] lifetime” (N = 1,230). (PDF) [file pgen.1011288.s024.pdf]

S14

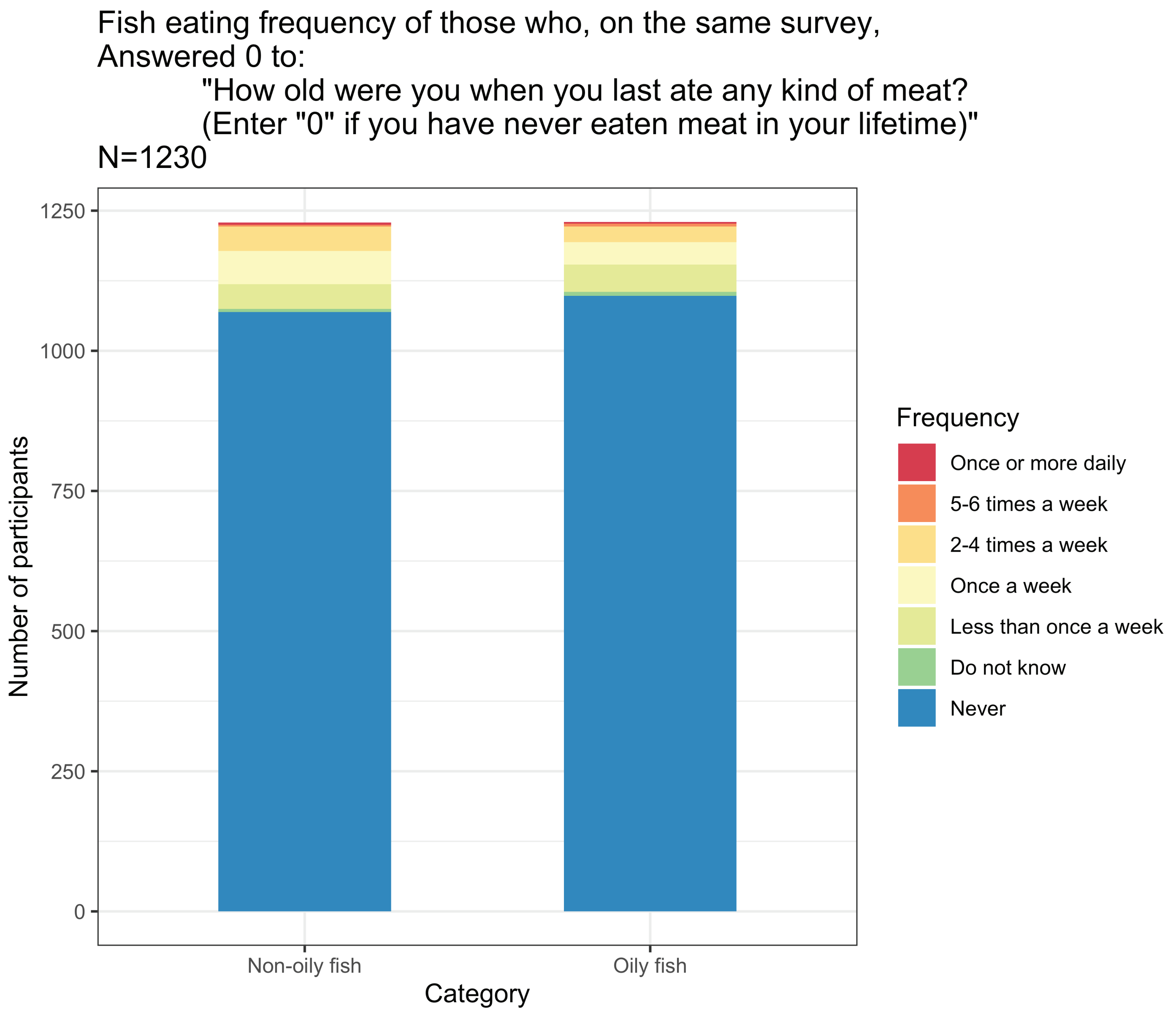

S14 Fig. Fish eating frequency of those who have "never eaten meat" in their lifetime. Bar plot shows non-oily-fish- and oily-fish-eating frequency, reported at the initial assessment, for those who reported on that same dietary survey that they had "never eaten meat in [their] lifetime" (N=1,230).
