# Supplementary material for: Gene-vegetarianism interactions in calcium, estimated glomerular filtration rate, and testosterone identified in genome-wide analysis across 30 biomarkers
Source: PLoS Genet. 2024 Jul 11;20(7):e1011288. doi: 10.1371/journal.pgen.1011288 (PMC11239071; doi:10.1371/journal.pgen.1011288)
Supplement: Attachment — Submitted filename: Response to Reviewers.docx [file pgen.1011288.s025.docx]

**Point-by-point response to reviewers: Gene-vegetarianism interactions in calcium, estimated glomerular filtration rate, and testosterone identified in genome-wide analysis across 30 biomarkers**

*Reviewers’ remarks are presented in italics*

>Our response in non-italics and colored blue.

>>> We thank the reviewers for their highly supportive comments and suggestions, which have been helpful in improving the quality of our work.

# Reviewer #1

*The authors present a GWAS on vegetarians vs non-vegetarians which they then use as a binary trait in a genome-wide interaction study for a 30 trait panel of clinical analytes relevant to human health. The paper is on the whole very well written, and was a pleasure to review. The group make excellent use of the UK BioBank dataset, including a very thoughtful and compelling use of the lifestyle questionnaire data which the leverage to curate a retrospective strict vegetarian cohort (in so far as the available data permit this). It is highly unusual for the cohort assembly and sample matching portion of any paper to be particularly compelling, but in this the rigorous whittling of the vegetarian cohort based on inconsistencies in the dietary questionnaire responses made for interesting reading.*

*Perhaps reflecting the diversity of reasons for the choice of vegetarianism as a lifestyle, and likely in part due to the rigor of the vegetarian cohort curation which resulted in a small cohort size, it is not surprising that no variants met the significance threshold for vegetarianism itself as a GWAS trait. Similarly, GWIS adjusted p-values were generally modest, approaching GWIS-adjusted statistical significance (passing genomewide-significance for individual traits but not when corrected for the 30 traits considered).*

*The authors highlight selected GWIS associations, but do not overstate their findings nor the implications. Interestingly, multiple GWIS variants map to the vitamin B12 pathway lending genetic support to the long-standing observation of B12 deficiency in strict vegetarians.

Interestingly, the a separate finding from the epidemiological component of this study calls into question the prevailing wisdom that vegetarianism is a universally beneficial lifestyle choice, with the authors noting higher plasma triglycerides and alkaline phosphatase and lower vitamin D, as well as replicating effects on urate and testosterone (the latter very modest).

While the implications of the study are somewhat limited (as the authors note), the study is very well designed, the analysis performed well, and the conclusions are appropriate to the data. This study is very appropriate for publication in PLOS Genetics. The authors should be commended for their inclusion of interactive web versions of their Miami plots.*

We agree with this assessment and thank the reviewer for the kind words. We are glad the reviewer enjoyed the paper and found it appropriate for publication in *PLOS Genetics*.

*Minor issues: The phrase "nearly significant" (p16/line 324) is not informative. It would be better if the sentence were reworded, and the non-significant adjusted p-val simply reported instead.*

We thank the reviewer for this comment. The *P*-value for C-reactive protein in females has been added to this sentence, which now reads:

ApoA was significant only in males, ALP and Lp (a) were significant only in females; C-reactive protein was nearly significant (*P*=0.002) in females (S4 Fig; S4 Table).

*The aesthetics in Fig 1 are odd, and distracting. Why is "first time taking" heavily outlined in Fig1b, and what is the point of the color gradient across the columns in Fig1c? Both are distracting and unnecessary.*

We thank the reviewer for this comment. Regarding Fig 1b, “first time taking” is heavily outlined because these were the 24-hour recall surveys considered as part of the criteria for defining vegetarians in this study; this is also indicated in the figure caption. We used these surveys because they were nearest to the biomarker collection time point. We agree that the color gradient in Fig 1c may be distracting and did not add meaningful information, and so have replaced it with a single color across bars.

*The gendered color differences (specifically Fig S2b, and also Fig S4) are unnecessarily subtle (to the point that I didn't initially realize that there was a different fill color for the box plots in Fig S2b). The authors are encouraged to increase the color saturation and/or opacity to make differences easier to distinguish. In fact, it would be more useful to group the data in S2b by sex rather than by cohort (i.e. rather than showing 3 cohorts each split by sex, show 2 sexes split by cohort - the male and female could then be displayed on axes ranged appropriate to the values by sex).*

We thank the reviewer for this comment. For S2b Fig, after looking at both arrangements, we prefer current stratification, however, we have increased the color saturation for females to increase distinction between these boxes. Regarding S4 Fig, we thank the reviewer for pointing this out; the sizes of the boxes on the forest plot have been increased by 50%.

*An expanded version of Table 2 would be a useful addition to the extensive supplemental data - perhaps highlighting the top ~5 variants for each trait and the adjusted p-val and beta for each in the cohort as a whole as well as stratified by sex.*

We thank the reviewer for this suggestion. We have added a new Supplementary Table S7 which lists all suggestively significant (*P*<1e-05) interactions from this study. Additionally, we have run sex-stratified interaction analyses on these suggestively significant variants, shown in the same table. We have updated the main manuscript text to include these results.

# Reviewer #2

*In this manuscript, Francis and colleagues have scrutinized UK Biobank participants, especially of European ethnicity that practice strict vegetarian diet for genome-wide gene-vegetarianism interaction across 30 metabolic biomarkers. Their gene-diet interaction analysis identified notable gene-vegetarianism interactions on calcium, estimated glomerular filtration rate and testosterone. As authors stated in the manuscript, genetic studies in the context of vegetarianism are limited. As the term "vegetarianism" refers to plant-based varying dietary habits, genetic associations especially with regard to health risk is delicate. It would be logical to explore the genetics of individuals that are strict vegetarians for several generations rather than those choose and practice as a lifestyle.*

We thank the reviewer for this comment. While we agree that this would be an interesting addition to our study, this information is not available for analysis. The only UK Biobank data field about length of dietary adherence is already included in our vegetarianism definition, which required two surveys indicating vegetarian adherence, as well as indicating there were no “major changes in diet over the past 5 years.” From our perspective, five years can be considered an adequate amount of time for biomarker levels to respond to dietary intake.

*A newly published GWAS by Yaseen et al. (2023) identified genetic loci linked to vegetarian diet among white caucasians participated in the UK Biobank study. As the authors have used the same dataset, I am wondering that they missed this GWAS study. It is surprising that two independent GWAS studies performed on the UK Biobank's European vegetarians didn't replicate. I suggest authors to compare their results from the third part (i.e., GWAS to search for variants that may explain vegetarianism behavior on a genetic level) with the significant hits from Yaseen et al.*

We thank the reviewer for this comment. The paper by Yaseen et al. was published for the first time (no pre-print) after we had submitted our manuscript for review to *PLOS Genetics*. It should be noted that the [“no scoop” policy of *PLOS Genetics*](https://journals.plos.org/plosgenetics/s/complementary-research)) is applicable, which precludes a study published during our peer review from “diminishing the novelty or significance” of our manuscript.

We have added reference to Yaseen et al. in our discussion to place our results in context with theirs:

We nominally replicated the single GWS variant found in a recent GWAS in UK Biobank Europeans (rs72884519) [1], and found a similar effect size (β=-0.09; *P*=0.002). We attribute the discrepancy in *P*-values between our study and theirs (*P*=4.997x10^−8^) to important differences in our respective vegetarianism definitions. Notably, their vegetarianism cohort included participants that completed only the IA and not the 24HR. There is no data field on the IA to self-identify directly as vegetarian, and we have described above how single time-point survey data can be unreliable [2-4]. Yaseen et al. [1] therefore included 2,566 vegetarians (~48% of their cases) that we did not classify as such. Furthermore, we cannot determine what is meant when their methods refer to “the diet by 24-hour recall questionnaire, which captured ~110K of the respondents,” [1] because the 24HR has spanned approximately 211,000 respondents since its release in September 2012 [5].

*1) In page 4 line 77, the following statement "...though a recent GWAS of vegetarianism found none" should be rephrased by citing Yaseen et al. 2023.*

*Yaseen NR, Barnes CLK, Sun L, Takeda A, Rice JP. Genetics of vegetarianism: A genome-wide association study. PLoS One. 2023 Oct 4;18(10):e0291305. doi: 10.1371/journal.pone.0291305. PMID: 37792698; PMCID: PMC10550162.*

This sentence in the introduction has been updated and now reads:

A recent GWAS in UK Biobank Europeans, which used a single survey instance to classify vegetarians, found one variant at genome-wide significance impacting vegetarianism status (rs72884519; *P*=4.997×10^-8^) [1], while a GWAS in a Japanese cohort found none [6].

*2) In page 5 line 84, correct the word "recommandations" to recommendations.*

We thank the reviewer for identifying this typo. It has been corrected in the text.

# Reviewer #3

*Reviewer #3: In this manuscript the authors describe a multistep study that probes the impact of vegetarianism and vegetarianism-gene interactions on clinical biomarkers in UK BioBank Participants. First, they used diverse convergent evidence to identify strict vegetarians in a manner that is more rigorous than many prior studies. Then they identified lower Vitamin D and cholesterol levels, but higher triglyceride levels in these strict vegetarians. In their first GWAS, no variants associated with vegetarianism (i.e,. no variants predicted the adoption of vegetarianism). In their second set of GWAS on the biomarkers, they identified a genome-wide significant variant-vegetarianism interaction with rs72952628 . Interestingly vegetarians with this variant had higher serum calcium levels. This variant is linked to changes in MMAA expression, a protein involved with B12 metabolism. Many vegetarians have a hard time getting enough B12 and some of its cellular transport is calcium dependent. On the gene level two significant interactions were identified: RNF168 with testosterone, and DOCK4 with estimated glomerular filtration rate (eGFR). Vegetarians with rs73219637 in RNF168 had higher testosterone, and the effect was present in both sexes. Vegetarians who were homozygous for rs17159341 in DOCK4 had lower eGFR. Overall, the manuscript is well written and if these results are corroborated in future studies, they may provide important early evidence for developing precision nutrition care.*

We agree with this summary of our study and thank the reviewer for the kind words.

*A few issues deserve consideration:

1) The authors should be applauded for using a multistep process to hone the definition and identification of strict vegetarians. The measurement of diet/environment is usually the most difficult and error prone part of exposure assessment in G-by-E analyses. However even with this robust approach, the vegetarians are not a homogenous group. Could the authors expand their discussion of this heterogeneity and possibly consider subgroup analyses on: 1) vegans and 2) vegetarians who eat fish?*

We thank the reviewer for this comment and agree that a section discussing vegans would improve this study. We applied our selection criteria to vegans, but found only 133 European vegans who did not otherwise indicate eating meat or had recent dietary changes. To avoid the risk of introducing unreliable results from this small sample, we prefer not to include analysis from this underpowered group of vegans. However, we have added a new table similar to Table 1, describing the dietary survey responses from the self-reported vegan sample, which can be found in the new S1 Table. We have also added details to the text in the first section of the results describing UK Biobank vegans.

In terms of those who self-reported as vegetarians and also reported eating fish, it is worth restating that our process in this experiment was to reliably categorize vegetarians in a case/control study design, guided by the common definition of vegetarianism (provided in the introduction) which does not include fish-eaters. It is therefore our recommendation that “vegetarians who eat fish” are not vegetarians, and we do not hypothesize that we would find vegetarianism effects in this group which is characterized by both vegetarianism signal dilution and small sample size (~10-14% of self-reported vegetarians as shown in Fig 1d). Additionally, interpreting these results would be difficult, as the survey data of these participants are not internally consistent enough to determine the specifics of their dietary intake, in contrast to our group of strict vegetarians. Finally, unlike with vegans, the stated concern of the reviewer with non-homogeneity among vegetarians is not an issue here, as the group of fish-eaters were categorized as non-vegetarian controls (this point is addressed further in the next comment and response).

*2) Along these lines, there were 964 people who only partially met vegetarian criteria and it appears that they were included as non-vegetarians in this analysis. It would be useful to see analyses with these participants excluded, rather than reclassified as nonvegetarians. This group may represent intermediate exposure to vegetarianism (or just a subpopulation for whom exposure misclassification is likely).*

We thank the reviewer for identifying this consideration. We have re-run our gene-vegetarianism interaction analysis for our three lead variants as a sensitivity analysis to determine whether the exclusion of the 964 participants, who did not report eating meat on the initial assessment but otherwise failed our vegetarianism criteria, would impact our results. As shown in the table below, the effects and *P*-values are nearly identical. We feel this is sufficient to demonstrate that the inclusion of these controls did not affect nor bias our results.

|  |  | **Original** | | **Remove 964** | |
| --- | --- | --- | --- | --- | --- |
| **Trait** | **SNP** | **BETA-int (SE)** | ***P*-int** | **BETA-int (SE)** | ***P*-int** |
| Calcium | rs72952628 | -0.437 (0.080) | 4.25×10^-8^ | -0.436 (0.080) | 4.38×10^-8^ |
| Testosterone | rs73219637 | -0.206 (0.042) | 7.13×10^-7^ | -0.206 (0.042) | 6.96×10^-7^ |
| eGFR | rs17159341 | 0.1989 (0.039) | 2.58×10^-7^ | 0.1987 (0.039) | 2.62×10^-7^ |

*3) Line 163 Exactly how were participants eliminated to remove relatives? Was there a pihat threshold? If so, was one of each pair removed in a random selection?*

We thank the reviewer for this comment. We have clarified the relevant text in the methods to now read as follows:

Additionally, we iteratively identified and removed each individual with the highest number of relationships, and removed the minimum number of participants to eliminate all related pairs.

*4) The authors should compare and contrast their work with that of Yaseen et al 2023* [*https://pubmed.ncbi.nlm.nih.gov/37792698/*](https://pubmed.ncbi.nlm.nih.gov/37792698/) *This study was less comprehensive than the current study, but its findings in a more heterogenous set of UK biobank vegetarians deserve discussion.*

This point was addressed at length in the response to Reviewer #2 above. Please consult our above response for details regarding our additions to the introduction and discussion for the purpose of fully considering and contextualizing this study.

*5) Brief additional discussion of the biomarkers themselves is warranted. For example: Creatinine is a component of meat and serum creatinine rises when you eat it. This lowers eGFR. In short, the exposure (not eating meat-creatinine) is a component of the outcome (serum creatinine based eGFR). This is an important issue for interpretation, as this way of elevating eGFR is almost tautological and it does not reflect kidney function. Thus this particular outcome may be a little problematic.*

We thank the reviewer for pointing out this potentially unclear aspect of our results. First, our eGFR formula considers both creatinine and cystatin C, which makes it less susceptible to bias than the creatinine-based eGFR (ref 62). Next, what makes this finding compelling to us is that in non-interactive traditional epidemiological analysis, we actually did not find a significant effect of vegetarianism on eGFR (Fig 2; *P*=0.17). Only when considering gene-vegetarianism interactions were we able to detect this significant difference in eGFR, which was dependent on the number of copies of the G allele at rs17159341 (Fig 4f).

*As for calcium and testosterone levels, it is unclear what might be good/desirable for any given person. Usually higher or lower is not a universal good and there may be sweet spots that differ between subgroups of participants. Mentioning these complexities should help the reader.*

We thank the reviewer for this consideration. We agree that for some biomarkers, such as calcium and testosterone, their health impacts are not linear, with both excessively low and high levels being detrimental. Our study did not aim to define the optimal levels for these biomarkers. Instead, we estimated the association effects of vegetarianism status on the biomarkers and identified genetic factors that modify these associations. Given a health objective for a specific biomarker, these two pieces of information will inform if the decision of adopting a vegetarian lifestyle is appropriate for an individual with a unique genetic background. Moreover, due to the different impacts of vegetarianism on various biomarkers, as revealed by our study, readers ought to consider these biomarkers comprehensively.

We added a line to the discussion to emphasize these complexities:

These results should be interpreted within the context of the specific nutritional needs of an individual.

**References**

1.	Yaseen NR, Barnes CLK, Sun L, Takeda A, Rice JP. Genetics of vegetarianism: A genome-wide association study. PLOS ONE. 2023;18(10):e0291305. doi: 10.1371/journal.pone.0291305.

2.	Burton PR, Hansell AL, Fortier I, Manolio TA, Khoury MJ, Little J, et al. Size matters: just how big is BIG?: Quantifying realistic sample size requirements for human genome epidemiology. International Journal of Epidemiology. 2009;38(1):263-73. doi: 10.1093/ije/dyn147.

3.	Grandits GA, Bartsch GE, Stamler J. Method issues in dietary data analyses in the Multiple Risk Factor Intervention Trial. The American journal of clinical nutrition. 1997;65(1):211S-27S.

4.	Francis M, Li C, Sun Y, Zhou J, Li X, Brenna JT, et al. Genome-wide association study of fish oil supplementation on lipid traits in 81,246 individuals reveals new gene-diet interaction loci. PLOS Genetics. 2021;17(3):e1009431. doi: 10.1371/journal.pgen.1009431.

5.	UK Biobank Data Showcase. Data-Field 20077. Available from: <https://biobank.ndph.ox.ac.uk/showcase/field.cgi?id=20077>.

6.	Nakamura Y, Narita A, Sutoh Y, Imaeda N, Goto C, Matsui K, et al. A genome-wide association study on meat consumption in a Japanese population: the Japan Multi-Institutional Collaborative Cohort study. Journal of Nutritional Science. 2021;10:e61. Epub 2021/10/11. doi: 10.1017/jns.2021.49.
